# Evolutionary Analysis of the *YABBY* Gene Family in Brassicaceae

**DOI:** 10.3390/plants10122700

**Published:** 2021-12-08

**Authors:** Yun-Hai Lu, Intikhab Alam, Yan-Qing Yang, Ya-Cen Yu, Wen-Chao Chi, Song-Biao Chen, Boulos Chalhoub, Li-Xi Jiang

**Affiliations:** 1 Institute of Crop Science, College of Agriculture and Biotechnology, Zhejiang University, Hangzhou 310058, China; yuyacen@zju.edu.cn (Y.-C.Y.); boulos.chalhoub@yahoo.com (B.C.); jianglx@zju.edu.cn (L.-X.J.); 2 Key Laboratory of Ministry of Education for Genetics, Breeding and Multiple Utilization of Crops, College of Crop Science, Fujian Agriculture and Forestry University, Fuzhou 350002, China; intikhabalam2013@gmail.com (I.A.); yanqing.yang2020@hotmail.com (Y.-Q.Y.); 3 Marine and Agricultural Biotechnology Laboratory, Institute of Oceanography, Minjiang University, Fuzhou 350108, China; chiwenchao@foxmail.com (W.-C.C.); songbiao_chen@hotmail.com (S.-B.C.)

**Keywords:** Brassicaceae, *YABBY* genes, gene duplication, evolution, gene expression

## Abstract

The *YABBY* gene family is one of the plant transcription factors present in all seed plants. The family members were extensively studied in various plants and shown to play important roles in plant growth and development, such as the polarity establishment in lateral organs, the formation and development of leaves and flowers, and the response to internal plant hormone and external environmental stress signals. In this study, a total of 364 *YABBY* genes were identified from 37 Brassicaceae genomes, of which 15 were incomplete due to sequence gaps, and nine were imperfect (missing C2C2 zinc-finger or YABBY domain) due to sequence mutations. Phylogenetic analyses resolved these *YABBY* genes into six compact clades except for a *YAB3*-like gene identified in *Aethionema arabicum*. Seventeen Brassicaceae species each contained a complete set of six basic *YABBY genes* (i.e., 1 *FIL*, 1 *YAB*2, 1 *YAB3*, 1 *YAB5*, 1 *INO* and 1 *CRC*), while 20 others each contained a variable number of *YABBY* genes (5–25) caused mainly by whole-genome duplication/triplication followed by gene losses, and occasionally by tandem duplications. The fate of duplicate *YABBY* genes changed considerably according to plant species, as well as to *YABBY* gene type. These *YABBY* genes were shown to be syntenically conserved across most of the Brassicaceae species, but their functions might be considerably diverged between species, as well as between paralogous copies, as demonstrated by the promoter and expression analysis of *YABBY* genes in two *Brassica* species (*B. rapa* and *B. oleracea*). Our study provides valuable insights for understanding the evolutionary story of *YABBY* genes in Brassicaceae and for further functional characterization of each *YABBY* gene across the Brassicaceae species.

## 1. Introduction

The *YABBY* gene family contains several transcription factor members present in all seed plants [1,2]. The family members have important functions in plant growth and development, such as the polarity establishment in lateral organs, the formation and development of leaves and flowers, and the response to internal plant hormone and external environmental stress signals [3,4]. The family was initially named after the Australian freshwater crayfish following the discovery of the first member, *CRABS CLAW*, of which the mutation (*crc-1*) can result in apically unfused carpels in *Arabidopsis thaliana* [5]. *YABBY* genes encode small proteins that all contain two conserved domains, an N-terminal C2C2 zinc-finger domain and a C-terminal helix-loop-helix domain (termed as *YABBY* domain) similar to a high-mobility group (HMG) box [6,7,8]. Six *YABBY* genes were identified in the model plant *A. thaliana* genome, including *FILAMENTOUS FLOWER* (*FIL*), *YAB2, YAB3*, *YAB5*, *INNER NO OUTER* (*INO*), and *CRABS CLAW (CRC)*, where *FIL* and *YAB3* represent the most recent gene duplication within the family [8]. *FIL*, *YAB2*, *YAB3*, and *YAB5* are called “vegetative” and shown to be preferentially expressed in leaves and leaf-derived organs (i.e., cotyledons, sepals, petals, stamens, and carpels), whereas *INO* and *CRC* are specifically expressed in developing floral organs (i.e., ovules and carpels respectively) that are evolutionarily derived from leaves [5,6,7,9,10,11,12]. These *YABBY* genes were initially and extensively studied in *Arabidopsis*, and they were shown to play important roles in the specification of abaxial cell fates in lateral organs produced by apical and flower meristems in both distinct and redundant manners [8]. *FIL* is required for the normal formation and development of inflorescence and floral meristems; its mutation (*fil*) generates clusters of both filamentous structures and flowers with floral organs of altered number and shape [7,9,13,14,15]. The *fil yab3* double mutant showed obvious changes in vegetative phenotypes (such as cotyledons and leaves with linear forms, abnormal vasculature and leaf surface with abaxial character, and ectopic shoot apical meristem structures) and displayed severely radialized floral organs [6]. The triple *fil yab3 yab5* mutants, as well as the quadruple *fil yab2 yab3 yab5* mutants, demonstrated more severe phenotype changes than the double *fil yab3* mutant; plants became diminutive and bushy, lost apical dominance and lamina expansion, and showed polarity defects in all lateral organs, whereas *yab2* and *yab5* mutants and the *yab2 yab5* double mutant exhibited a leaf morphology similar to wild-type [12,16]. *INO* is necessary for polarity determination in the central part of the ovule, ensuring the normal formation and asymmetric growth of the ovule outer integument; its mutation (*ino*) makes the outer integument fail to initiate and develop on the abaxial side of the ovule [10]. *CRC* is required for normal carpel development by suppressing radial growth of the developing gynoecium but promoting its longitudinal growth, in addition to being important for nectary development; its mutation (*crc*) generates shorter and wider gynoecia with the two carpels being unfused at the apex and flowers lacking nectaries [5,17].

Due to their potential important roles in plant growth and development revealed in *A. thaliana*, the *YABBY* genes have been extensively studied for their functions in various other plants such as rice [18,19,20,21,22,23,24,25,26,27], wheat [28], maize [29,30,31], sorghum [32], *Antirrhinum majus* [33], tomato [34], *California poppy* (*Eschscholzia californica*) [35], sugar apple (*Annona squamosa*) [36], grapevine [37,38], and *Brassica rapa* [39,40]. Through a genome-wide survey, eight *YABBY* genes were identified and characterized in the rice [41], nine in tomato [42], eight in common bean [43], 17 in soybean [44], 23 in upland cotton [45], seven in grapevine [37], nine in pineapple [46], six in pomegranate [47], 54 in eight orchid species [48], and 55 in seven magnoliid species [49]. Phylogenetic studies revealed that the angiosperm *YABBY* genes can be clustered into five subfamilies, named *FIL*/*YAB3*, *YAB2*, *YAB5*, *INO*, and *CRC*, and the last common ancestor of extant angiospermous plants should have at least five *YABBY* genes [1,50]. It was suggested that the last common ancestor of extant seed plants had only one or two *YABBY* genes already acting as polarity genes [2].

The Brassicaceae family comprises widely diverse morphotypes of plants and provides a most extensive and varied range of valuable products (such as oil, vegetables, dietary fiber, condiments, and vitamin C) for human use [51,52]. The family has a wide worldwide distribution; therefore, it has been and is still exposed to a large number of environmental parameters which might have contributed to its high genetic and morphological diversity on the earth. The family includes 3973 species in 341 genera and 52 tribes [53], which are further grouped into three major lineages (I, II, and III) [54,55,56] or six clades (A–F) [57,58]. The family contains both the most popular model plant *Arabidopsis* and the economically important *Brassica* crops. The diploid Brassica species *B*. *rapa* (AA, 2n = 20), *B*. *nigra* (BB, 2n = 16), and *B*. *oleracea* (CC, 2n = 18) formed the tetraploid species *B. juncea* (AABB, 2n = 36), *B. napus* (AACC, 2n = 38), and *B. carinata* (BBCC, 2n = 34), among which their cytogenetic relationships were demonstrated in the early 20th century by Asian cytogeneticists Morinaga and U in U’s triangle theory [59,60]. This study aimed to clarify the evolutionary distribution of the different *YABBY* family members among the Brassicaceae genomes, as well as gain insights into the possible roles of *YABBY* genes in the development and evolution of Brassicaceae species. We performed genome-wide identification, classification, and evolution analysis of *YABBY* genes among 37 Brassicaceae species (including the model species *A. thaliana*). We further analyzed the phylogenetic relationships between the *YABBY* genes of *Brassica* diploid and allotetraploid species and compared the expression patterns between two diploid *Brassica* species *B. rapa* and *B. oleracea*. Our study provides valuable information for better understanding the evolutionary history of this small gene family in Brassicaceae, as well as further functional characterization of *YABBY* genes among various Brassicaceae species (especially the *Brassica* species).

## 2. Results

### 2.1. Genome-Wide Identification of YABBY Genes in 37 Brassicaceae Species

A total of 364 YABBY homologous protein sequences were identified from the genomes of 37 Brassicaceae species. Their designed corresponding gene name, original gene ID, physical position on the corresponding chromosome/scaffold, and encoded putative protein size (aa) are summarized in Table S1. Their protein sequences are summarized in Figure S1. In 15 cases, we obtained only partial YABBY protein sequences due to incomplete genome sequencing data. In nine other cases, we obtained imperfect YABBY protein sequences (with imperfect C2C2 zinc-finger or YABBY domain) due to sequence changes/mutations at the DNA level. Concretely, for *BjuCRCc*, the first exon of the original *YABBY* gene was absent due to sequence deletion in the genome; for *CamINOd*, the initial gene was truncated by a stop codon; for *CamINOe*, the fourth exon of was initial gene was absent due to internal sequence deletion; for *EveFILb*, the first exon of the original gene was partial due to sequence deletion; for *ItiYAB3a*, the first three exons of the original gene were absent due to sequence deletion; for *LanCRCa* and *LanCRCb*, the last exons of the original genes were absent due to sequence deletion; for *SpaYAB*2, the first exon of the original gene was translocated from the chromosome Sp1 to Sp7; for *SpiYAB3*, the YABBY domain was imperfect due to sequence deletion in the original gene. 

The identified 364 Brassicaceae *YABBY* genes could be classified into six types according to their similarity to the six *A. thaliana YABBY* genes: 77 *FIL*, 77 *YAB2*, 49 *YAB3*, 43 *YAB5*, 65 *INO*, and 53 *CRC*. The distributions of each type of *YABBY* genes identified in the 37 Brassicaceae species are summarized in Table 1. We can observe that the number of *YABBY* genes identified per species varied from five to 25 according to the complexity of genome: 0–3 for *YAB3* and *YAB5*, 1–3 for *CRC*, 0–5 for *INO*, and 0–7 for *FIL* and *YAB2.* Only five *YABBY* genes (one *YAB3*, two *YAB5*, and two *CRC*) were identified in the genome of *Stanleya pinnata*, while as many as *25 YABBY* genes (seven *FIL*, seven *YAB*2, two *YAB3*, two *YAB5*, four *INO*, and three *CRC*) were identified in the allotetraploid *B. juncea*. Seventeen species (including *A. thaliana*) each had the six basic *YABBY* genes, i.e., one *FIL,* one *YAB2,* one *YAB3,* one *YAB5,* one *INO,* and one *CRC.* These six basic *YABBY* genes were perfectly duplicated by two in *Caulanthus amplexicaulis* (with two *FIL*, two *YAB2*, two *YAB3*, two *YAB5*, two *INO*, and two *CRC*) and triplicated by three in *Camelina* sativa (with three *FIL*, three *YAB2*, three *YAB3*, three *YAB5*, three *INO*, and three *CRC)*. Remarkably, no *FIL*, *YAB2* and *INO* homologs were found in *Stanleya pinnata*, no *YAB3* homolog was found in *Leavenworthia alabamica*, and no *YAB5* homolog was found in *Lepidium sativum*. From chromosomal location data, we identified three pairs of tandem duplicated *YABBY* genes: two in *B. junceae* (*BjuFILa*–*BjuFILg*, *BjuYAB2b*–*BjuYAB2g*) and one in *Sisymbrium irio* (*SirYAB2a*–*SirYAB2a*). 

### 2.2. Phylogenetic Analysis of YABBY Genes among Brassicaceae Species

**Figure 1:**
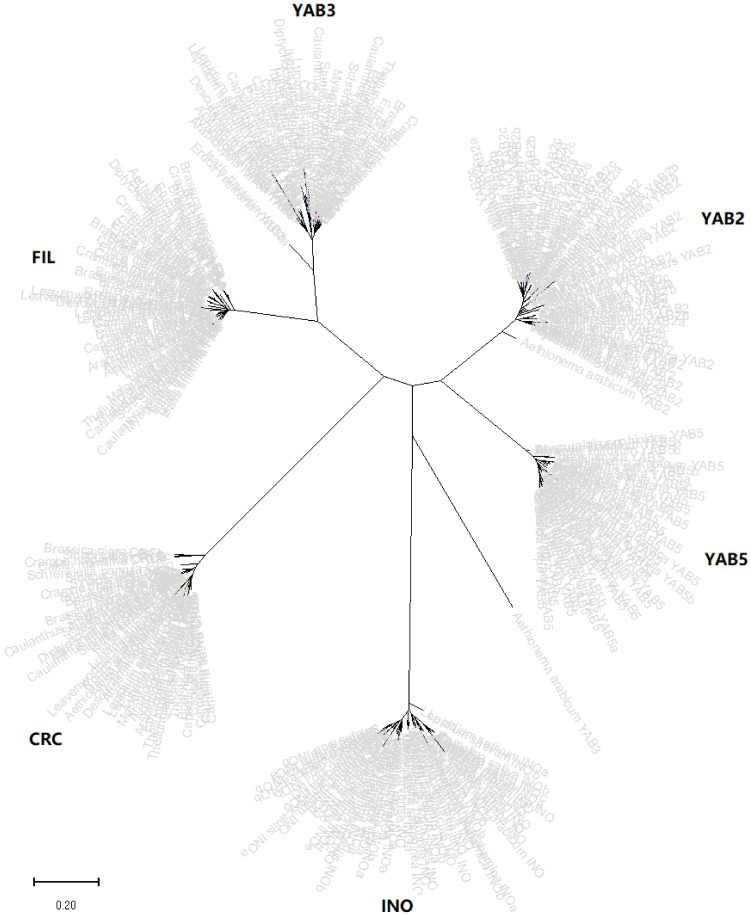
Phylogenetic tree of 301 YABBY protein sequences identified from 34 Brassicaceae species. Brassica napus, Brassica juncea, and Brassica carinata were here excluded as their ancestor diploid species Brassica rapa, Brassica oleracea, and Brassica nigra were already included in the analysis. The tree was generated through MEGA7 using the maximum likelihood method and Jones–Taylor–Thornton (JTT) matrix-based model.

A phylogenetic tree was firstly constructed for 301 YABBY genes identified from 34 Brassicaceae species (*B. napus*, *B. juncea*, and *B. carinata* were here excluded as their ancestor species *B. rapa, B. oleracea*, and *B. nigra* were already included in the analysis) on the basis of their deduced encoding protein sequences (Figure 1). The result showed that all these *YABBY* genes could be clustered into six compact clades, named *FIL*, *YAB2*, *YAB3*, *YAB5*, *INO*, and *CRC*, except for *Aethionema arabicum YAB3* (*AarYAB3*), which was clearly distinguished from other Brassicaceae *YABBY* genes. 

**Figure 2:**
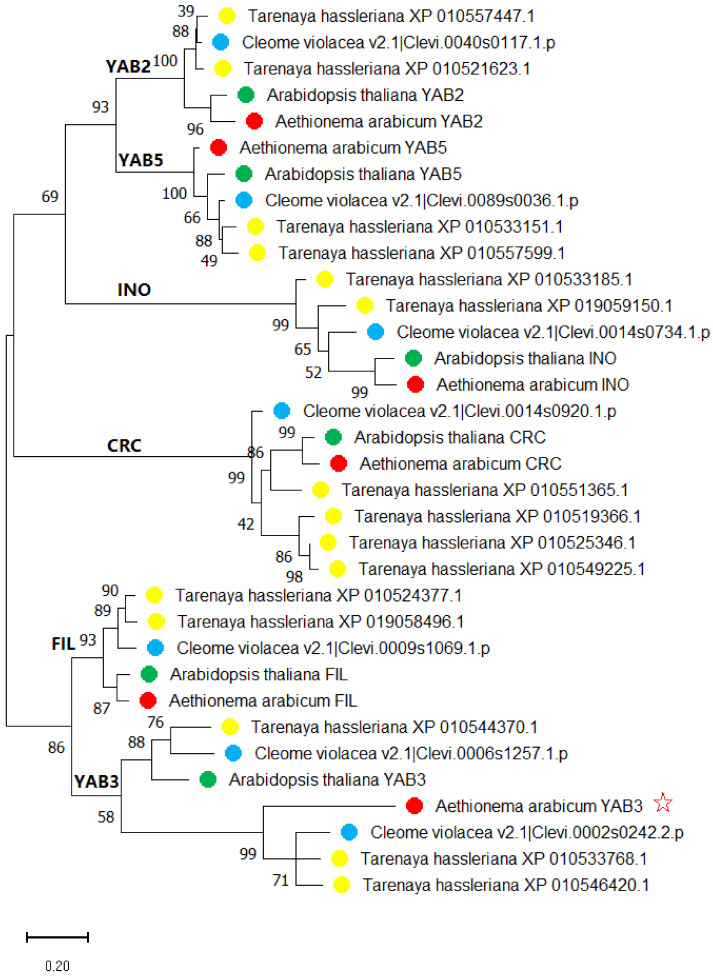
Phylogenetic tree based on six Aethionema arabicum, six Arabidopsis thaliana, seven Cleome violacea, and 15 Tarenaya hassleriana YABBY protein sequences. The tree was generated through MEGA7 using the maximum likelihood method and Jones–Taylor–Thornton (JTT) matrix-based model with 1000 bootstrap replicates. The red star indicates the YAB3-like protein sequence identified from the Aethionema arabicum genome.

To understand the origin of *AarYAB3*, we performed BLAST searches at NCBI using AarYAB3 as a query sequence. We found two closely related YABBY sequences, i.e., XP_010533768.1 (220 aa) and XP_010546420.1 (219 aa), in *Tarenaya hassleriana*, which belongs to *Cleomaceae*, a sister family to Brassicaceae [61]. We also checked the genome sequence data of another Cleomaceae species *Cleome violacea* at Phytosome 13 and identified seven homologous *YABBY* genes, of which one (Clevi.0002s0242) was closely related to *AarYAB3*. A phylogenetic tree was then generated on the basis of six *Aethionema arabicum,* six *A. thaliana*, seven *Cleome violacea*, and 15 *Tarenaya hassleriana* YABBY protein sequences (Figure 2). The result showed that AarYAB3 was closely clustered together with two *Tarenaya hassleriana* and one *Cleome violacea* YABBY sequences, and they formed a distinct subgroup compared to its sister subgroup containing the *A. thaliana YAB3* plus one *Tarenaya hassleriana* (XP010544370.1) and one *Cleome violacea* (Clevi.0006s1257.1.p) YABBY sequences. Interestingly, all five other *Aethionema arabicum* YABBY sequences, i.e., AarFIL, AarYAB2, AarYAB5, AarINO, and AarCRC, were tightly clustered together with their Arabidopsis counterparts, except for AarYAB3.

To gain a better view of the phylogenetic relationships between the *YABBY* genes in each of the six YABBY clades (or subfamilies), we further generated six individual phylogenetic trees on the basis of the protein sequences of 60 FIL, 60 YAB2, 43 YAB3, 38 YAB5, 53 INO, and 47 CRC genes, identified from the 34 Brassicaceae species cited above (Figure S2A–F). From these trees, we can observe that the orthologous *YABBY* genes from closely related Brassicaceae species or the recent duplicated paralogous *YABBY* members from a single species tended to be clustered together with high bootstrap support, whereas the paralogous *YABBY* gene members, caused by a whole-genome triplication (WGT) event occurring ~15.9 million years ago (Mya) and shared by all the species of the tribe Brassiceae [62,63] were resolved into different subgroups. 

### 2.3. Phylogenetic Relationships among YABBY Genes of U’s Triangle Brassica Diploid and Allotetraploid Species

**Figure 3:**
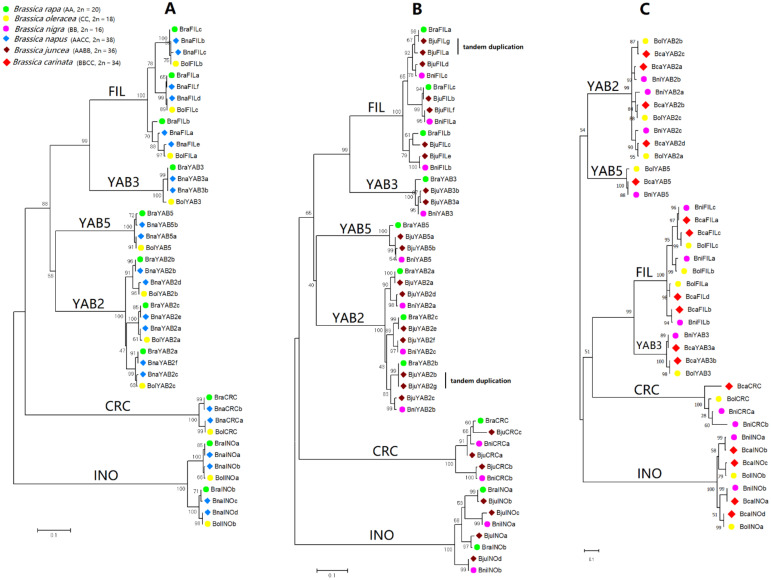
Phylogenetic relationships among *YABBY* genes of U’s triangle *Brassica* diploid and allotetraploid species. Three phylogenetic trees were generated on the basis of YABBY protein sequences: one based on the YABBY sequences identified from *B. rapa* (AA), B. oleracea (CC), and B. napus (AACC) (**A**), one based on the YABBY sequences from B. rapa (AA), B. nigra (BB), and B. juncea (AABB) (**B**), and one based on the YABBY sequences from B. nigra (BB), B. oleracea (CC), and B. carinata (BBCC) (**C**). The trees were generated through MEGA7 using the maximum likelihood method and Jones–Taylor–Thornton (JTT) matrix-based model with 1000 bootstrap replicates.

To illustrate the phylogenetic relationships among *YABBY* genes of U’s triangle *Brassica* diploid and allotetraploid species, we generated three phylogenetic trees: one based on YABBY sequences of *B. rapa* (AA), *B. oleracea* (CC), and *B. napus* (AACC) (Figure 3A), one based on YABBY sequences of *B. rapa* (AA), *B. nigra* (BB), and *B. juncea* (AABB) (Figure 3B), and one based on YABBY sequences of *B. nigra* (BB), *B. oleracea* (CC), and *B. carinata* (BBCC) (Figure 3C). In each tree, the orthologous *YABBY* genes from three related species were tightly clustered together, and each *YABBY* gene of allotetraploid species was clustered side by side with its corresponding ortholog in diploid species. We can see clearly that the *B. napus* genome contained 22 YABBY genes, of which 11 originated from *B. rapa*, and the other 11 originated from *B. oleracea*; the *B. juncea* genome contained *25 YABBY genes* (instead of 12 + 11 = 23 as expected), of which 11 originated from *B. rapa*, 12 originated from *B. nigra*, and two originated from intragenomic gene duplications by tandem (*BjuFILa*–*BjuFILg*, *BjuYAB2b*–*BjuYAB2g*); the *B. carinata* genome contained 16 *YABBY* genes (instead of 12 + 11 = 23 as expected), of which nine originated from *B. nigra* and seven originated from *B. oleracea* (Table S1). In the *B. carinata* genome, seven expected *YABBY* genes (two *FIL*, two *YAB2*, two *CRC*, and one *YAB5*) were not found from the actual version of the genome sequence database. However, a DNA fragment containing exon 1 of *YAB5* was identified on chromosome C01 (named *Bcayab5* in Table S1, not included for the *YABBY* gene number count in this study).

### 2.4. Syntenic Relationships among YABBY Genes of Different Brassicaceae Species

Syntenic genes are those located in homologous genomic fragments where the order of flanking genes is conserved across different species that originated from an identical ancestor; thus, they are orthologous and often share similar biological functions [64]. To gain an idea about the degree of syntenic conservation of these *YABBY* genes among different Brassicaceae species, we retrieved the syntenic data from BRAD database available for 173 *YABBY* genes of 18 Brassicaceae species, and the data are summarized in Table S2. The result showed that *YAB2* was the most conserved on syntenic genomic fragment “tPCK1—Block A” (with a ratio of 36/37) across the genomes of 18 Brassicaceae species, followed by *FIL* on “tPCK3—Block J” (30/37), *INO* on “tPCK1—Block B” (29/37), *CRC* on “tPCK6—Block E” (23/37), *YAB3* on “tPCK5—Block O” (18/37), and *YAB5* on “tPCK3—Block I” (16/37).

### 2.5. Putative cis-Regulatory Element Analysis of B. rapa and B. oleracea YABBY Genes

**Figure 4:**
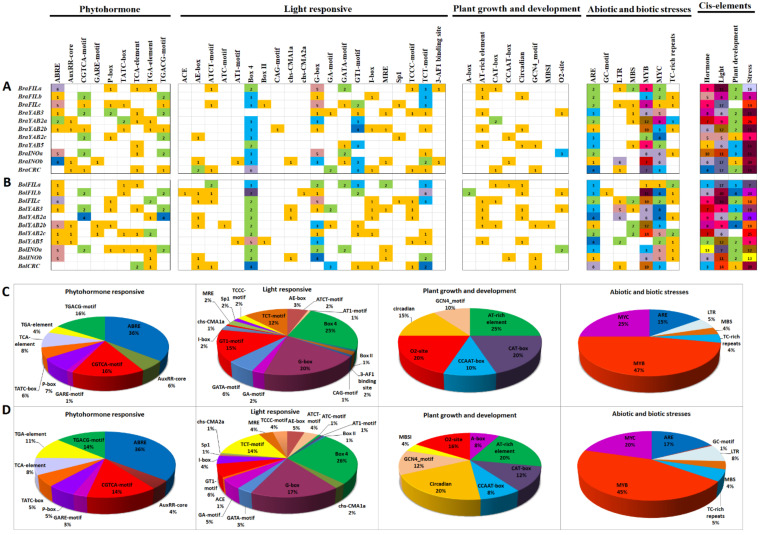
Analysis of putative *cis*-regulatory elements in the promoter regions (2 kb) of *Brassica rapa* (**A**,**D**) and *Brassica oleracea* (**B**,**C**) *YABBY* genes. The number of different putative *cis*-regulatory elements predicted in the 2 kb promoter region of each *B. rapa* (**A**) and *B. oleracea* (**B**) *YABBY* gene is given at the corresponding position and highlighted by different grid colors. The sum of the *cis*-regulatory elements in each group for each *YABBY* gene promoter is displayed with different colors on the upper right side of the figure. The percentage values of different *cis*-regulatory elements in each group are illustrated by pie charts for *B. rapa* (**C**) and *B. oleracea* (**D**). ABRE, ABA-responsiveelement; AuxRR-core, core of the auxin response region; GARE-motif, GA-responsive motif; ACE, light responsiveness; AE-box, part of a module for light response; MRE, Myb-recognition element; MBSI, MYB binding site I; ARE, anaerobic-responsive element; LTR, low temperature responsiveness; MBS, MYB binding site; MYB, MYB binding site; MYC, MYC binding site.

cis-Regulatory elements play important roles in the process of downstream gene expression and regulation through interaction with transcription factors. To get an idea about the types and distributions of cis-regulatory elements in the promoter region of *YABBY* genes, we analyzed the 2 kb genomic DNA sequences upstream of the ATG start site of each *B. rapa* and *B. oleracea YABBY* gene. A total of 371 and 384 putative cis-regulatory elements were predicted from the promoter sequences of 11 *B. rapa* and 11 *B. oleracea YABBY* genes, respectively. According to their involvement in different biological processes, these elements were classified into four groups: phytohormone-related (nine), light-related (20), growth and development-related (eight), and stress-related (seven) (Figure 4A,B). In both species, ABRE, CGTCA, and TGACG in phytohormone-related, G-box, GT1, and Box 4 in light-related, AT-rich, O_2_-site, and circadian in growth and development-related, and ARE, MYB, and MYC in stress-related groups were detected with a relatively high frequency (Figure 4C,D). However, the total number of detected cis-regulatory elements in the growth and development-related group was significantly lower than that in the other three groups. Variations in the composition of cis-regulatory elements of the promoter region were found among the duplicate *YABBY* genes (such as among *FILa*, *FILb*, *FILc*, etc.) in both species.

### 2.6. Expression Analysis of YABBY Genes in B. rapa and B. oleracea

**Figure 5:**
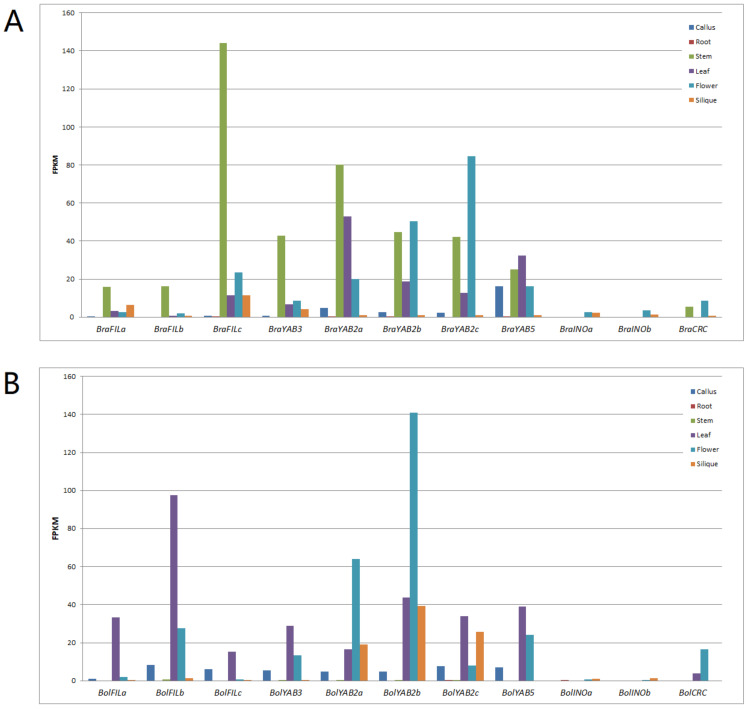
Expression pattern analysis of *YABBY* genes in *Brassica rapa* (**A**) and *Brassica oleracea* (**B**). The expression levels of 11 *B. rapa* (**A**) and 11 *B. oleracea* (**B**) *YABBY* genes in six different tissues, namely, callus, root, stem, flower, leaf, and silique, were calculated from RNA-seq data obtained from the GEO database at NCBI (GSE43245 and GSE42891) and displayed by histograms with different colors. FPKM, numbers of fragments per kilobase of transcript per million mapped reads.

To gain information about the expression pattern of the *B. rapa* and *B. oleracea YABBY* genes, we analyzed their RNA-seq data available from the GEO database at NCBI. Figure 5 shows the expression patterns of the 11 *B. rapa* and 11 *B. oleracea YABBY* genes in six different tissues, namely, callus, root, stem, leaf, flower, and silique. Globally, all *YABBY* genes were expressed in at least one of the six tested tissues, and the “vegetative” *YABBY* genes were more highly expressed than the “floral” *YABBY* genes in both *B. rapa* and *B. oleracea*. In both two species, these “vegetative” *YABBY* genes tended to be highly expressed in leaf and flower but were not or very lowly expressed in root and callus. The orthologous *YABBY* genes tended to conserve their expression patterns to some degree between the two species. For example, *BraYAB2b* and *BraYAB2c* were highly expressed in flower in *B. rapa*, while their corresponding orthologs *BolYAB2b* and *BolYAB2a* were also highly expressed in flower in *B. oleracea*; *BraYAB2a* was highly expressed in leaf in *B. rapa*, while its ortholog *BolYAB2c* was also highly expressed in leaf in *B. oleracea*. On the other hand, some spectacular differences were also observed between the two species. For example, all except two *YABBY* genes (*BraINOa* and *BraINOb*) were highly expressed in stem in *B. rapa*, while their orthologs were not or only lowly expressed in stem in *B. oleracea*; *BraFILa*, *BraFILb*, *BraFILc*, and *BraYAB3* were preferentially expressed in stem in *B. rapa*, while their orthologs *BolFILc*, *BolFILa*, *BolFILb*, and *BolYAB3* were highly expressed in leaf in *B. oleracea*; *BolYAB2a*, *BolYAB2b*, and *BolYAB2c* were highly expressed in silique in *B. oleracea*, while their orthologs *BraYAB2c*, *BraYAB2b*, and *BraYAB2a* were only lowly expressed in silique in *B. rapa*.

According to the involvement of possible stress-responsive cis-regulatory elements in the promoter region, we investigated the expression of two “vegetative” *YABBY* genes (*YAB3* and *YAB5*) in response to salt (200 mM NaCl) and drought (10% (*w*/*v*) PEG6000) stresses in both *B. rapa* and *B. olerace.* The qPCR results showed that these two *YABYY* genes were responsive to the two stresses in both species with roughly similar expression patterns (Figure S3).

## 3. Discussion

In this study, we carefully examined the publicly available genomic databases of 37 Brassicaceae species (including *A. thaliana*) and identified a total of 364 *YABBY* genes (Figure S1, Table S1). A phylogenetic analysis resolved these identified YABBY sequences into six compact clades (FIL, YAB2, YAB3, YAB5, INO, and CRC) (Figure 1), in accordance with previous studies in eudicot plants [1,50]. Among the 37 Brassicaceae species, 17 (including *A. thaliana*) each contained a set of six basic *YABBY* genes, i.e., one *FIL*, one *YAB2*, one *YAB3*, one *YAB5*, one *INO*, and one *CRC*, whereas one (named *Sisymbrium irio*) contained a set of six basic YABBY genes plus an additional *YAB2* member originated by duplication by tandem in the genome. This set of six basic *YABBY* genes was perfectly duplicated in *Caulanthus amplexicaulis* and triplicated in *Camelina sativa*, caused obviously by recent whole-genome duplication (WGD) and triplication (WGT), respectively, which occurred during the evolutionary history of their genomes [65,66]. The remaining 19 species each contained a variable number of *YABBY* genes with a variable composition, which is a consequence of WGD or WGT that occurred in these species followed by the extensive loss of duplicated or triplicated genes in the process of rediploidization [67]. Our result indicated that the common ancestor of extant Brassicaceae species also contained a set of six basic *YABBY* genes like the model species *A. thaliana*. Each basic *YABBY* gene was conserved across most of the Brassicaceae species not only at the protein sequence level but also at the chromosomal location level (syntenically) (Table S2), indicating that the basic functions of these *YABBY* genes should also be conserved across these Brassicaceae species.

Interestingly, the basal Brassicaceae species *Aethionema arabicum* also contained a set of six *YABBY* genes, but its *YAB3* representeds a distinct type that was not found in all other 36 Brassicaceae species but found in *Tarenaya hassleriana* and *Cleome violacea*, both belonging to Cleomaceae, a sister family to Brassicaceae (Figure 2). This indicated that there already existed two forms of *YAB3* (resulting from an early duplication event) in the common ancestor of Brassicaceae species (as is the case in *Tarenaya hassleriana* and *Cleome violacea*, where both forms of *YAB3* coexist, see Figure 2), and only one of the two forms, essentially the form of *A. thaliana YAB3*, was maintained in the most extant Brassicaceae species. This reflects the intermediate phylogenetic position of *Aethionema arabicum* between Brassicaceae and its sister family Cleomaceae, and it provides new insight into the phylogeny and early diversification of Brassicaceae.

Our phylogenetic trees (Figure S2A–F) based on FIL, YAB2, YAB3, YAB5, INO, or CRC protein sequences were globally in accordance with the actual classification of Brassicaceae species [54,55,56,57,58]. These trees allowed identifying the closely related orthologous *YABBY* genes from different Brassicaceae species, as well as the recent duplicated paralogous *YABBY* genes from a single specific species. For example, we can deduce from the trees that the five *FIL* and six *YAB2* from *Eruca vesicaria* were the result of a WGT event followed by another more recent WGD event (Figure S2A,B), the 18 *YABBY* genes from *Camelina sativa* were the result of a very recent WGT event, and the 12 *YABBY* genes from *Caulanthus amplexicaulis* were the result of a more ancient WGD event. It was estimated that the WGT event (by hybridization of the three sub-genomes in quick succession) in *Camelina sativa* occurred very recently, probably emerging during the rapid expansion of agricultural practices ~5–10,000 ya [65], while the WGD event in *Caulanthus amplexicaulis* occurred ~10 Mya [66]. Interestingly, no duplicate *YABBY* gene was lost in *Caulanthus amplexicaulis* despite the ~10 million years of evolution, indicating that the YABBY genes of *Caulanthus amplexicaulis* underwent a relaxed selection during evolution.

The retention or loss of duplicate or triplicate genes may be affected by the internal functional needs of a plant species [68,69]. Our result showed that the duplicates or triplicates of *FIL* and *YAB2* were preferentially retained in some Brassicaceae species, especially in Brassica species, as well as in *Cakile maritima*, *Crambe hispanica*, and *Eruca vesicaria*, while those of *YAB5*, *YAB3*, and *CRC* displayed a tendency to be lost following the WGD or WGT events (Table 1). As extreme cases, *YAB5* was absent (while five other members were all duplicated) in *Lepidium sativum*, *YAB3* was absent in *Leavenworthia alabamica*, and all three *YABBY* genes, i.e., *FIL*, *YAB2*, and *INO*, were simultaneously absent in *Stanleya pinnata*, indicating that the species might have undergone particular natural constraints during its evolution, a hypothesis supported by the fact that *Stanleya pinnata* can develop normally in a wide range of hard edaphic environments such as soils with high sodium, boron, or selenium content or serpentine soils [70,71,72]. Interestingly, the WGD event in *Stanleya pinnata* was estimated to have occurred ~10.65 Mya [67], similar to that (~10 Mya) of *Caulanthus amplexicaulis* [65], but the fates of duplicate *YABBY* genes were very different between the two species, both of which belong to the Thelypodiae tribe. These variations in the number and composition of different types of *YABBY* genes observed in certain Brassicaceae species may have partially contributed to the high morphological disparity that was observed among Brassicaceae species [73]. For example, the losses of *FIL*, *YAB2*, and *INO* in *Stanleya pinnata* may have partially contributed to the formation of distinct morphotypes such as pinnatifid leaves, unusual floral structures with spirally coiled anthers, and long-stalked seedpods.

Our comparative analysis between *Brassica* allopolyploid and diploid species revealed that *B. napus* retained all the *YABBY* genes of its diploid progenitors *B. rapa* (11) and *B. oleracea* (11); *B. juncea* also retained all the *YABBY* genes from its diploid progenitors *B. rapa* (11) and *B. nigra* (12), and gained two additional *YABBY* genes by tandem duplications; however, *B. carinata* only retained nine of 12 *YABBY* genes from *B. nigra* and seven of 11 *YABBY* genes from *B. oleracea* (Table S1), i.e., seven *YABBY* genes (two *FIL*, two *YAB2*, one *YAB5*, and two *CRC*) were lost or degenerated following the polyploidization event. The birth time of *B. carinata* was estimated as ∼0.047 Mya, which is only slightly earlier than that of *B. napus* (estimated as ∼0.043 Mya) but significantly later than that of *B. juncea* (estimated as ∼0.076 Mya) [74]. This implies that the *YABBY* genes underwent different selection pressures in the three allopolyploid *Brassica* species. The discovery of a partial segment (exon 1) of *YAB5* on chromosome C01 of *B. carinata (Bcayab5* in Table S1) indicated an ongoing process of degeneration of duplicate *YABBY* genes from the genome.

In this study, we also identified seven “imperfect” *YABBY* genes, i.e., with imperfect C2C2 zinc-finger or *YABBY* domain, in several Brassicaceae species (Table S1). As both domains are essential to ensure the basic functions of a *YABBY* gene [7,75], these ‘imperfect’ *YABBY* genes should have lost their functions in the related species. Some of these mutations, especially the simultaneous mutations of both two *CRC* genes in *Lunaria annua* (*LanCRCa* and *LanCRCb*), the mutation of unique *YAB2* in *Schrenkiella parvula* (*SpaYAB2*), and the mutation of unique *YAB3* in *Stanleya pinnata* (*SpiYAB3*), could have affected the growth and development of plants and, thus, might have contributed to the evolution of the morphological traits of the species. According to this logic, the loss of functions of both *CRC* genes in *Lunaria annua* might be related to its fragrant round flat seedpods.

Our cis-regulatory element analysis revealed an important number of phytohormone-responsive, light-responsive, and stress-related elements in the promoter sequences of both *B. rapa* and *B. oleracea YABBY* genes (Figure 4), suggesting that expression of these *YABBY* genes can be regulated by internal hormones and environmental signals. This result is consistent with previous studies in common bean [43], soybean [44], cotton [45], and pineapple [46], where the *YABBY* gene members were shown to be responsive to abiotic stresses. On the other hand, these cis-elements were not conserved among the duplicate paralogous *YABBY* genes and even less among the orthologous *YABBY* genes of *B. rapa* and *B. oleracea*, implying that these paralogous or orthologous *YABBY* genes might be differently regulated and, thus, have different spatial and temporal expression patterns during the growth and development of *B. rapa* and *B. oleracea*.

In Arabidopsis, *FIL*, *YAB2*, *YAB3*, and *YAB5* are called ‘vegetative’, and they are expressed in both leaves and floral organ primordia, whereas *CRC* and *INO* are specifically expressed in developing carpels and ovules, respectively [1,5,6,10]. Our RNA-seq data analysis revealed that both *B. rapa* and *B. oleracea YABBY* genes conserved more or less similar expression patterns to their *Arabidopsis* orthologous genes, i.e., the “vegetative” *YABBY* genes were generally expressed in leaf and flower, while the “reproductive” *YABBY* genes were mainly expressed in flower (Figure 5). This implies that their basic biological functions should be maintained across the different species. However, spectacular differences in RNA-seq expression patterns were observed between *B. rapa* and *B. oleracea YABBY* genes, e.g., nine out of 11 *B. rapa YABBY* genes (including *BraCRC*) were highly expressed in stem while all the *B. oleracea YABBY* genes were not or very lowly expressed in the same tissue, and the three *B. oleracea YAB2* were highly expressed in silique while their counterparts were very lowly expressed. In addition, obvious differences were also observed between paralogous *YABBY* genes when comparing the expression patterns in both *B. rapa* or *B. oleracea*, e.g., differences in both expression level and expression pattern could be observed among the duplicate *FIL* and *YAB2* of *B. rapa* or *B. oleracea*. This result is consistent with our analysis of cis-regulatory elements and indicates that the functions of duplicate *YABBY* genes might have diverged during the growth and development of both *B. rapa* and *B. oleracea*. We can deduce that the duplicate *YABBY* genes in other Brassicaceae species might have also functionally diverged similarly to those of *B. rapa* or *B. oleracea*, as recently demonstrated in *B. napus* [76].

## 4. Materials and Methods

### 4.1. Identification of YABBY Protein Genes

Six *Arabidopsis* YABBY protein sequences were first downloaded from the *Arabidopsis* database TAIR *(*http://www.arabidopsis.org/, accessed on 15 January 2021) and then used as query sequences for BLASTp and tBLASTn searches against the sequence databases of Phytozome v13 *(*https://phytozome-next.jgi.doe.gov/, accessed on 15 January 2021), BRAD (http://brassicadb.cn/, accessed on 15 January 2021), and NCBI (https://www.ncbi.nlm.nih.gov/, accessed on 15 January 2021). At BRAD, we searched the genomes of 18 species, namely, *Aethionema arabicum, Arabidopsis halleri*, *Arabidopsis lyrata*, *Arabidopsis thaliana*, *Boechera stricta*, *Brassica rapa*, *Brassica nigra*, *Brassica oleracea*, *Brassica juncea*, *Brassica napus*, *Camelina sativa*, *Capsella grandiflora*, *Capsella rubella*, *Leavenworthia alabamica*, *Schrenkiella parvula*, *Sisymbrium irio*, *Thellungiella halophile*, *and Thellungiella salsuginea*. At Phytozome v13, we searched the genomes of other 18 species, namely, *Alyssum linifolium*, *Cakile maritima*, *Caulanthus amplexicaulis*, *Crambe hispanica*, *Descurainia sophioides*, *Diptychocarpus strictus*, *Eruca vesicaria*, *Euclidium syriacum*, *Iberis amara*, *Isatis tinctoria*, *Lepidium sativum*, *Lunaria annua*, *Malcolmia maritima*, *Myagrum perfoliatum*, *Rorippa islandica*, *Sinapis alba*, *Stanleya pinnata*, *and Thlaspi arvense*. At NCBI, we searched the genome of one species, *Brassica carinata*. The identified YABBY homologous sequences were then checked visually and/or by SMART (http://smart.embl-heidelberg.de/, accessed on 15 January 2021) for the presence of both C2C2 zinc-finger and YABBY domains. For some identified YABBY homologs that necessitated further verifications because of the presence of unusual sequence, we checked their local genomic sequence on the genome to see if there existed sequence gaps; we reannotated these genes using FGENESH (http://www.softberry.com/, accessed on 15 January 2021) and verified their gene structures by Artemis [77]. These identified YABBY sequences were then compared with the six known A. thaliana YABBY proteins (FIL, YAB2, YAB3, YAB5, INO, and CRC) by MEGA X [78] and classified according to those of A. thaliana. A specific YABBY gene name was then designed to each of the identified YABBY protein sequence in different species (see Table S1).

### 4.2. Phylogenetic Analysis

Phylogenetic analyses were conducted in MEGA X [78]. YABBY protein sequences were aligned using Clustal W in MEGA X with default parameters and manually edited by BioEdit software (http://en.bio-soft.net/format/BioEdit.html, accessed on 15 January 2021). The phylogenetic tree was generated using the maximum likelihood method and Jones–Taylor–Thornton (JTT) matrix-based model [79] with 1000 bootstrap replicates. 

### 4.3. Syntenic Relationships

Syntenic relationships were established among the *YABBY* genes identified from 18 Brassicaceae species for which the whole-genome sequencing data were available at BRAD database. For each of the six *A. thaliana YABBY* genes, we identified its syntenic *YABBY* genes in other Brassicaceae species using the ‘Search Syntenic Gene’ function provided by the BRAD database. We obtained then the information concerning the syntenic gene name (s) and their localization on Translocation Proto-Calepineae Karyotype (tPCK) chromosomes and ancestral chromosome blocks, as well as on least fractioned (LF), medium fractionated (MF1), and most fractionated (MF2) subgenomes (if existing) [61,62,80].

### 4.4. Promoter Region Analysis

*B. rapa* and *B. oleracea* are two diploid progenitor species of the tetraploid species *B. napus*. All three species are cultivated widely around the world as edible vegetables and for their oils. We chose *B. rapa* and *B. oleracea* as demonstrative species to perform the analysis of cis-regulatory elements in promoter regions, as well as the analysis of expression patterns (see the next paragraph) of different *YABBY* family members. The promoter regions (upstream 2-kb genomic DNA sequences from the start codon ATG) of all *B. rapa* and *B. oleracea YABBY* genes were obtained from BRAD. The putative generic sequence files were then subjected to Plant-CARE (http://bioinformatics.psb.ugent.be/webtools/plantcare/html/, accessed on 15 January 2021) for online analysis about the presence of cis-acting regulatory elements in each promoter sequence.

### 4.5. Expression Analysis of YABBY Genes in B. rapa and B. oleracea

The RNA-seq data of six tissues (i.e., callus, root, stem, leaf, flower, and silique) of *B. rapa* (accession Chiifu-401–42) and *B. oleracea* (sp. capitata homozygous line 02–12) were obtained from the GEO database at NCBI (http://www.ncbi.nlm.nih.gov/geo/, accessed on 15 January 2021) with accession numbers GSE43245 and GSE42891, respectively [81,82]. The expression levels (by fragments per kilobase of exon model per million mapped, FPKM) of each *B. rapa* and *B. oleracea YABBY* gene in the six tissues were extracted from the two RNA-seq datasets and submitted to statistical analysis. The expression analysis of two “vegetative” *YABBY* genes by qPCR in *B. rapa* and *B. oleracea* is described in the legend of Figure S3.

## 5. Conclusions

In conclusion, we identified a total of 364 *YABBY* genes in 37 Brassicaceae genomes using the publicly available whole-genome sequence databases. These *YABBY* genes were further characterized by their protein size, functional domains, chromosomal location, phylogeny classification, and syntenic relationships. We analyzed the variation in the number and types of *YABBY* genes in different Brassicaceae species. We also analyzed the phylogenetic relationships among *YABBY* genes of U’s triangle *Brassica* diploid and allotetraploid species, the promoter regions of both *B. rapa* and *B. oleracea YABBY* genes for cis-regulatory elements, and the expression patterns of *B. rapa* and *B. oleracea YABBY* genes. Our study provides valuable insights for understanding the evolutionary story of *YABBY* genes in Brassicaceae and for further functional characterization of each *YABBY* gene across the Brassicaceae species.

## Figures and Tables

**Table 1:** Distribution of *YABBY* family members in 37 Brassicaceae genomes.

Species	Ligneage	Clade	*FIL*	*YAB2*	*YAB3*	*YAB5*	*INO*	*CRC*	Total
*Aethionema arabicum*	-	F	1	1	1 ^a^	1	1	1	6
*Alyssum linifolium*	I	D	1	2	1	2	2	2	10
*Arabidopsis halleri*	I	A	1	1	1	1	1	1	6
*Arabidopsis lyrata*	I	A	1	1	1	1	1	1	6
*Boechera stricta*	I	A	1	1	1	1	1	1	6
*Arabidopsis thaliana*	I	A	1	1	1	1	1	1	6
*Brassica rapa*	II	B	3	3	1	1	2	1	11
*Brassica nigra*	II	B	3	3	1	1	2	2	12
*Brassica oleracea*	II	B	3	3	1	1	2	1	11
*Brassica juncea*	II	B	7	7	2	2	4	3	25
*Brassica napus*	II	B	6	6	2	2	4	2	22
*Brassica carinata*	II	B	4	4	2	1	4	1	16
*Cakile maritima*	II	B	3	3	2	1	5	2	16
*Camelina sativa*	I	A	3	3	3	3	3	3	18
*Capsella grandiflora*	I	A	1	1	1	1	1	1	6
*Capsella rubella*	I	A	1	1	1	1	1	1	6
*Caulanthus amplexicaulis*	II	B	2	2	2	2	2	2	12
*Crambe hispanica*	II	B	3	3	1	1	2	2	12
*Descurainia sophioides*	I	A	1	1	1	1	1	1	6
*Diptychocarpus strictus*	III	E	1	1	1	1	1	1	6
*Eruca vesicaria*	II	B	5	6	2	1	2	1	17
*Euclidium syriacum*	III	E	1	1	1	1	1	1	6
*Iberis amara*	II	C	2	3	2	1	2	1	11
*Isatis tinctoria*	II	B	4	2	3	1	3	2	15
*Leavenworthia alabamica*	I	A	2	1	0	1	2	1	7
*Lepidium sativum*	I	A	2	2	2	0	2	2	10
*Lunaria annua*	II	C	3	2	2	1	2	2	12
*Malcolmia maritima*	I	A	1	1	1	1	1	1	6
*Myagrum perfoliatum*	II	B	1	1	1	1	1	1	6
*Rorippa islandica*	I	A	1	1	1	1	1	1	6
*Schrenkiella parvula*	II	B	1	1	1	1	1	1	6
*Sinapis alba*	II	B	3	3	1	1	2	3	13
*Sisymbrium irio*	II	B	1	2 ^b^	1	1	1	1	7
*Stanleya pinnata*	II	B	0	0	1	2	0	2	5
*Thellungiella halophila*	II	B	1	1	1	1	1	1	6
*Thellungiella salsuginea*	II	B	1	1	1	1	1	1	6
*Thlaspi arvense*	II	B	1	1	1	1	1	1	6
Total			77	77	49	43	65	53	364

^a^ Atypical *YAB3*; ^b^ the two copies were tandemly duplicated.

## Data Availability

The genomic sequences of *YABBY* genes of the following 18 species: *Aethionema arabicum*, *Arabidopsis halleri*, *Arabidopsis lyrata*, *Arabidopsis thaliana*, *Boechera stricta*, *Brassica rapa*, *Brassica nigra*, *Brassica oleracea*, *Brassica juncea*, *Brassica napus*, *Camelina sativa*, *Capsella grandiflora*, *Capsella rubella*, *Leavenworthia alabamica*, *Schrenkiella parvula*, *Sisymbrium irio*, *Thellungiella halophile*, and *Thellungiella salsuginea* are available in the BRAD database (http://brassicadb.cn/, accessed on 15 June 2021). The genomic sequences of *YABBY* genes of the following 18 species: *Alyssum linifolium*, *Cakile maritima*, *Caulanthus amplexicaulis*, *Crambe hispanica*, *Descurainia sophioides*, *Diptychocarpus strictus*, *Eruca vesicaria*, *Euclidium syriacum*, *Iberis amara*, *Isatis tinctoria*, *Lepidium sativum*, *Lunaria annua*, *Malcolmia maritima*, *Myagrum perfoliatum*, *Rorippa islandica*, *Sinapis alba*, *Stanleya pinnata*, and *Thlaspi arvense* are available in the Phytozome v13 database (https://phytozome-next.jgi.doe.gov/, accessed on 15 June 2021). The genomic sequences of *YABBY* genes of *Brassica carinata* are available in the GenBank database of NCBI (https://www.ncbi.nlm.nih.gov/, accessed on 15 June 2021). The RNA-seq data of gene expression of six tissues (callus, root, stem, leaf, flower, and silique) of *B. rapa* (accession Chiifu-401–42) and *B. oleracea* (sp. *capitata* homozygous line 02–12) are available in the GEO database of NCBI (http://www.ncbi.nlm.nih.gov/geo/, accessed on 15 June 2021) under accession numbers GSE43245 and GSE42891, respectively. All other datasets supporting the results of this article are included within the article and its Supplementary Tables.

## Supplementary Materials

The following are available online at https://www.mdpi.com/article/10.3390/plants10122700/s1, Figure S1: List of 364 YABBY homologous protein sequences (fasta format) identified from the 37 Brassicaceae genomes, Figure S2: Phylogenetic trees based on the protein sequences of 60 FIL (A), 60 YAB2 (B), 43 YAB3 (C), 38 YAB5 (D), 53 INO (E) and 47 CRC (F) genes, respectively, identified from 34 Brassicaceae genomes, Figure S3: Expression analysis of two “vegetative” YABBY genes in *B. rapa* and *B. oleracea* under salt (A,B) and drought (C,D) stresses, Table S1: List of the *YABBY* family members identified in 37 Brassicaceae species, Table S2: Syntenic relationships between *YABBY* genes of different Brassicaceae species.

## Abbreviations

*FIL*: *FILAMENTOUS FLOWER*; *INO*: *INNER NO OUTER*; *CRC*: *CRABS CLAW*; HMG: high-mobility group; *TOB*: *TONGARI-BOUSHI*; GA: gibberellin acid; *DL*: *DROOPING LEAF*; *drl*: *drooping leaf*; BRAD: Brassica Database; SMART: Simple Modular Architecture Research Tool; JTT: Jones–Taylor–Thornton; tPCK: Translocation Proto-Calepineae Karyotype; LF: least fractioned; MF1: medium fractionated; MF2: most fractionated; GEO: Gene Expression Omnibus; RNA-Seq: RNA sequencing; FPKM: fragments per kilobase of exon model per million mapped; WGD: whole-genome duplication; WGT: whole-genome triplication; Mya: million years ago.
