# Supplementary material for: Evolutionary Analysis of the YABBY Gene Family in Brassicaceae
Source: Plants (Basel). 2021 Dec 8;10(12):2700. doi: 10.3390/plants10122700 (PMC8704796; doi:10.3390/plants10122700)
Supplement: Supplementary file 1 [file plants-10-02700-s001.zip › Figure S3.pdf]

Figure S3 Expression analysis of two “vegetative” *YABBY* genes in *B. rapa* and *B. oleracea* under salt (A,B) and drought (C,D) stresses. The detail analysis method was provided hereafter following the figure.

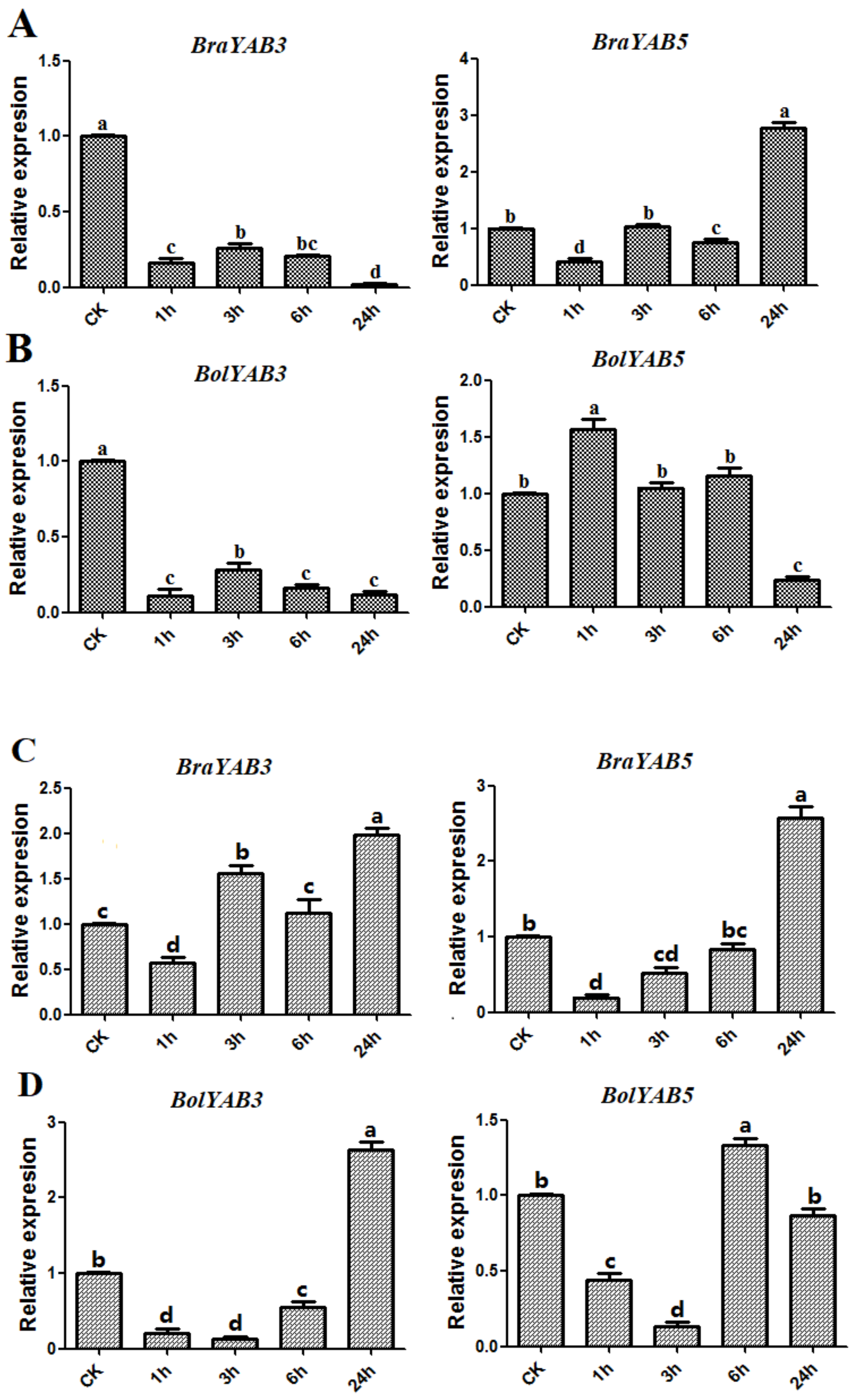

Plant materials and stress treatments

B. rapa L. ssp. *pekinensis* (Zhongbai 81) and *B. oleracea* L. ssp. *Capitata* (Zhonggan 11) seeds were sown in a Petri dish with moisture-absorbent filter papers and incubated at 25°C. The germinated seeding was then transferred into plastic pots containing a mixture of soil and vermiculite (3:1) grown in artificial growth chamber at 22 °C with a photoperiod of 16/8 h for light/dark and humidity at 65–70%. Three weeks old seedlings were used for two abiotic stress treatments. For salinity and PEG treatments, the seedlings were irrigated with 200 mM NaCl and 10% (w/v) polyethylene glycol (PEG 6000). Then, leaves from control and Stress treated plants were harvested after 0, 1, 3, 6, and 24 h of treatments and immediately immersed in liquid nitrogen, and stored at -80 °C until RNA extraction use. For each treatment, three biological replicates were prepared to decrease the error rate.

RNA extraction, cDNA synthesis and qPCR analysis

Total RNA was isolated from the frozen leaves of each sample of *B. rapa* and *B. oleracea* using a Plant RNA extraction Kit, following the manufacturer's instructions (OMEGA, China). The quality of RNA was checked by agarose gel electrophoresis, and also by using the NanoDrop 2000 Spectrophotometer (Thermo Fisher Scientific, Inc., Waltham, MA, USA). First-strand cDNA was synthesized using 1μg of total RNA per sample with the cDNA Synthesis Kit from TaKaRa Bio Inc. (Dalian, China). The reverse transcription products were diluted 20-fold and stored at −20 °C prior to analysis. Gene-specific primers for the selected *YABBY* genes for *B. rapa* and *B. oleracea* were designed using Primer3Plus software (http://www.primer3plus.com/). Specific primers for *YABBY* genes were used in qRT-PCR and Actin primers for *B. rapa* and *B. oleracea* were used as a control (Additional file x: Table x). The qRT-PCR analysis was performed on an ABI 7500 Fast Real-time PCR amplification system (Applied Biosystems, Foster, CA, USA). The analysis was carried out in a total volume of 20 μL containing 2 μL template of cDNA, 0.8 μL of the forward and reverse primers (10 μM), 10μL of SYBR Green PCR Master (ROX) (Roche, Shanghai, China), 6.4 μL of sterile distilled water. The PCR amplification parameters were as following: 95 °C for 1 minute, followed by 40 cycles of 95 °C for 15 s, and 60 °C for 70 s. For each sample, three replicates were run to compute the average Ct values. The data were analyzed using the $2^{-\Delta\Delta Ct}$ methods (Schmittgen et al. 2008). The relative expression levels of each sample were normalized by housekeeping genes (BraActin-2 and BolActin-2). The significance of differences was calculated by one way ANOVA followed by Tukey HSD test by using IBM SPSS version 22 and different letters indicate significant difference ($p < 0.05$).

Primers used for qPCR analysis

Genes		Primer sequences (5'-3')
BraYAB3	Forward	TGCCTGATCATCCTCCTACC
	Reverse	GGTCACACCAACATTTGCAG
BraYAB5	Forward	GCAGCAGTCTGTTCGACATC
	Reverse	CTGGAAATTAGGGCGTGAAA
BolYAB5	Forward	TGCACCAATCTGTGGTCTGT
	Reverse	AATTTTGGTGTGGCCTCTTG
BolYAB3	Forward	AGCTTTTGCGACACTGTCCT
	Reverse	ACGGAGGGAAGAAGAAGAGC
BraActin2	Forward	TCAGATGCCCAGAAGTCTTGTTCC
	Reverse	TCCTCCACCTGCCTCATCATACTC
BolActin2	Forward	CGTGACCTTACTGACTACC
	Reverse	CTCCATCTCCTGCTCGTA
