# Supplementary material for: Evolutionary Analysis of the YABBY Gene Family in Brassicaceae
Source: Plants (Basel). 2021 Dec 8;10(12):2700. doi: 10.3390/plants10122700 (PMC8704796; doi:10.3390/plants10122700)
Supplement: Supplementary file 1 [file plants-10-02700-s001.zip › Figure S2.pdf]

Figure S2 Phylogenetic trees based on the protein sequences of 60 *FIL* (A), 60 *YAB2* (B), 43 *YAB3* (C), 38 *YAB5* (D), 53 *INO* (E) and 47 *CRC* (F) genes, respectively, identified from 34 Brassicaceae genomes.

A

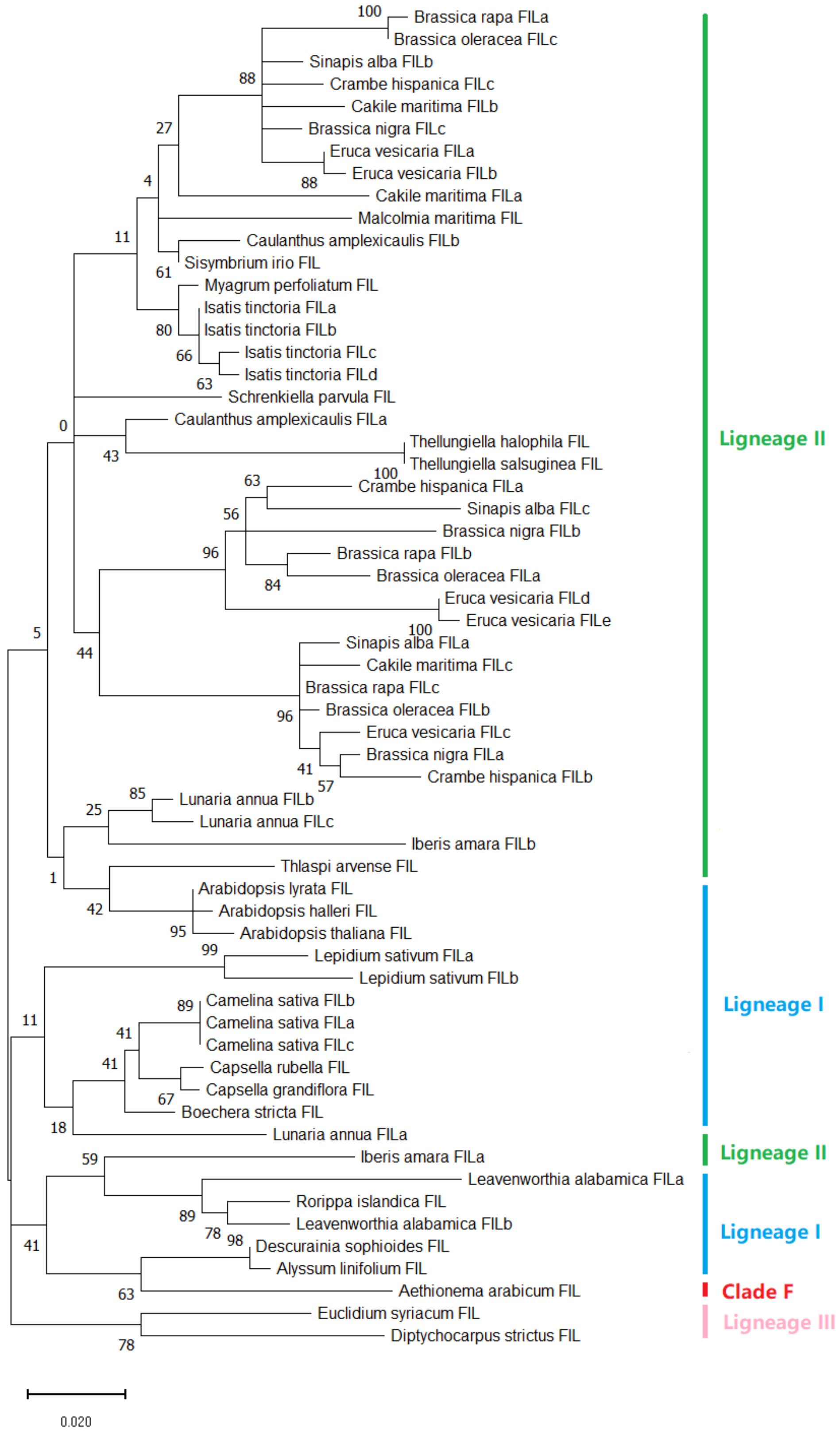

B

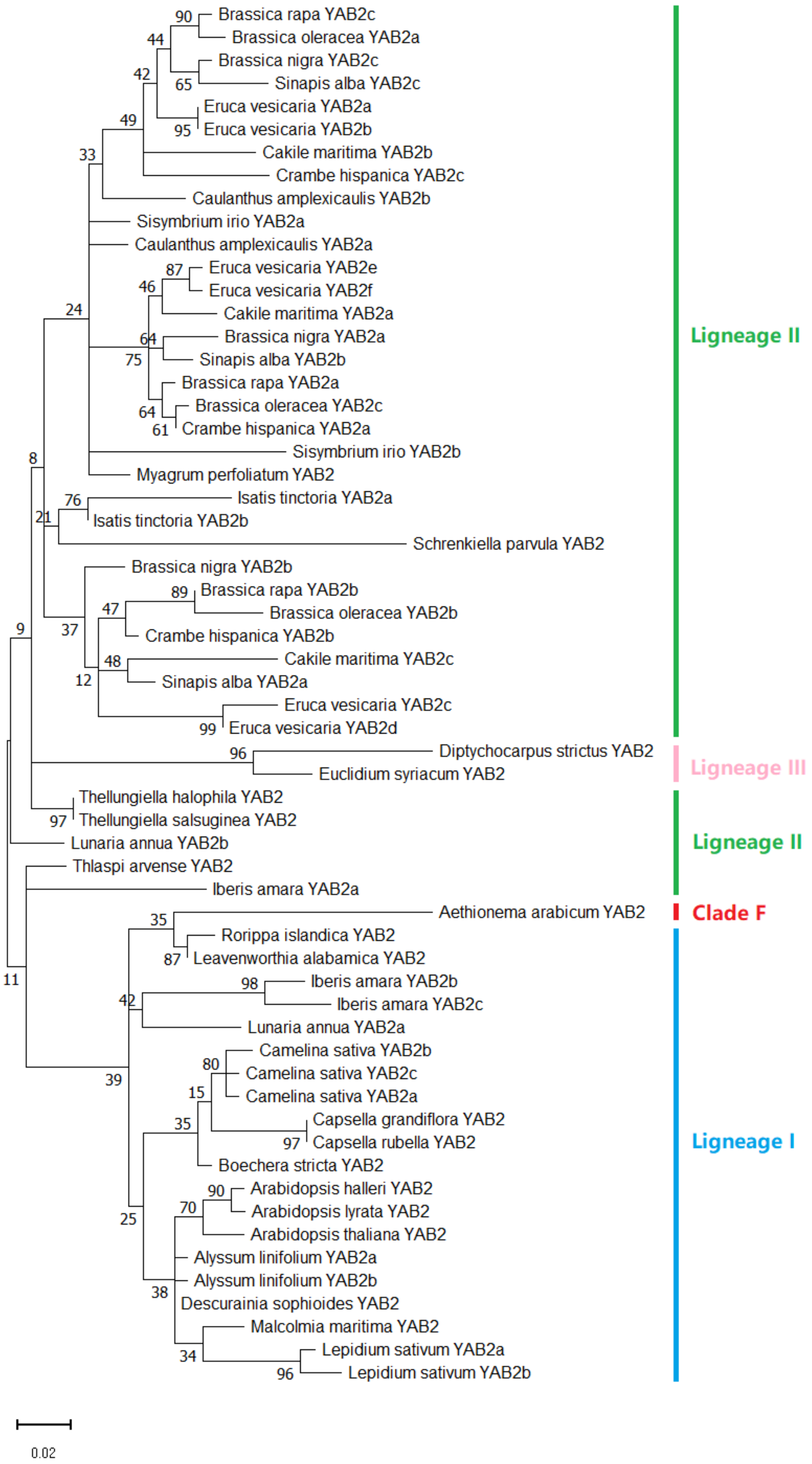
Brassica rapa YAB2c
Brassica oleracea YAB2a
Brassica nigra YAB2c
Sinapis alba YAB2c
Eruca vesicaria YAB2a
Eruca vesicaria YAB2b
Cakile maritima YAB2b
Crambe hispanica YAB2c
Caulanthus amplexicaulis YAB2b
Sisymbrium irio YAB2a
Caulanthus amplexicaulis YAB2a
Eruca vesicaria YAB2e
Eruca vesicaria YAB2f
Cakile maritima YAB2a
Brassica nigra YAB2a
Sinapis alba YAB2b
Brassica rapa YAB2a
Brassica oleracea YAB2c
Crambe hispanica YAB2a
Sisymbrium irio YAB2b
Myagrum perfoliatum YAB2
Isatis tinctoria YAB2a
Isatis tinctoria YAB2b
Schrenkiella parvula YAB2
Brassica nigra YAB2b
Brassica rapa YAB2b
Brassica oleracea YAB2b
Crambe hispanica YAB2b
Cakile maritima YAB2c
Sinapis alba YAB2a
Eruca vesicaria YAB2c
Eruca vesicaria YAB2d
Diptychocarpus strictus YAB2
Euclidium syriacum YAB2
Thellungiella halophila YAB2
Thellungiella salsuginea YAB2
Lunaria annua YAB2b
Thlaspi arvense YAB2
Iberis amara YAB2a
Aethionema arabicum YAB2
Rorippa islandica YAB2
Leavenworthia alabamica YAB2
Iberis amara YAB2b
Iberis amara YAB2c
Lunaria annua YAB2a
Camelina sativa YAB2b
Camelina sativa YAB2c
Camelina sativa YAB2a
Capsella grandiflora YAB2
Capsella rubella YAB2
Boechera stricta YAB2
Arabidopsis halleri YAB2
Arabidopsis lyrata YAB2
Arabidopsis thaliana YAB2
Alyssum linifolium YAB2a
Alyssum linifolium YAB2b
Descurainia sophioides YAB2
Malcolmia maritima YAB2
Lepidium sativum YAB2a
Lepidium sativum YAB2b
Ligneage II
Ligneage III
Ligneage II
Clade F
Ligneage I
0.02

C

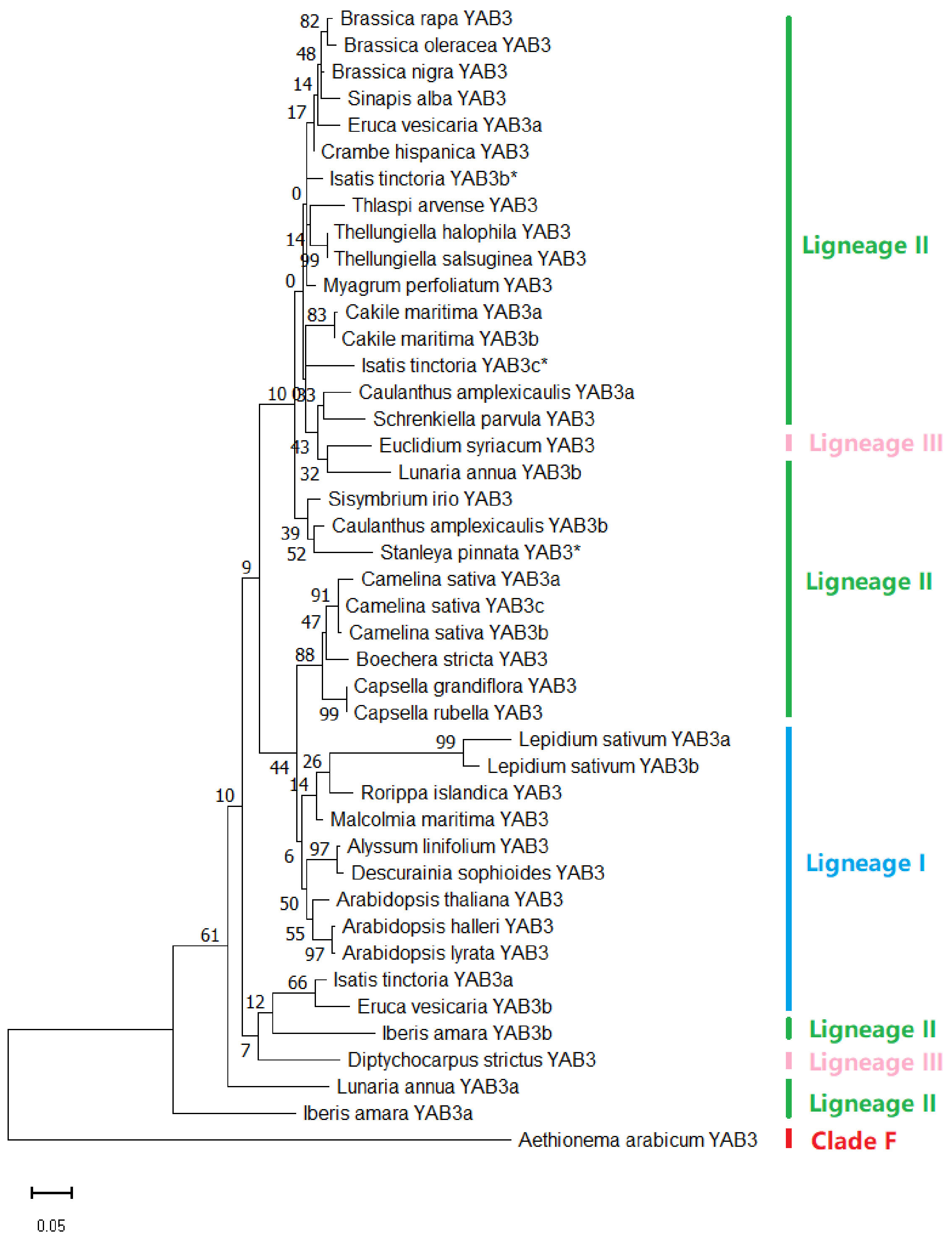

Brassica rapa YAB3
Brassica oleracea YAB3
Brassica nigra YAB3
Sinapis alba YAB3
Eruca vesicaria YAB3a
Crambe hispanica YAB3
Isatis tinctoria YAB3b*
Thlaspi arvense YAB3
Thellungiella halophila YAB3
Thellungiella salsuginea YAB3
Myagrum perfoliatum YAB3
Cakile maritima YAB3a
Cakile maritima YAB3b
Isatis tinctoria YAB3c*
Caulanthus amplexicaulis YAB3a
Schrenkiella parvula YAB3
Euclidium syriacum YAB3
Lunaria annua YAB3b
Sisymbrium irio YAB3
Caulanthus amplexicaulis YAB3b
Stanleya pinnata YAB3*
Camelina sativa YAB3a
Camelina sativa YAB3c
Camelina sativa YAB3b
Boechera stricta YAB3
Capsella grandiflora YAB3
Capsella rubella YAB3
Lepidium sativum YAB3a
Lepidium sativum YAB3b
Rorippa islandica YAB3
Malcolmia maritima YAB3
Alyssum linifolium YAB3
Descurainia sophioides YAB3
Arabidopsis thaliana YAB3
Arabidopsis halleri YAB3
Arabidopsis lyrata YAB3
Isatis tinctoria YAB3a
Eruca vesicaria YAB3b
Iberis amara YAB3b
Diptychocarpus strictus YAB3
Lunaria annua YAB3a
Iberis amara YAB3a
Aethionema arabicum YAB3
Ligneage II
Ligneage III
Ligneage II
Ligneage I
Ligneage II
Ligneage III
Ligneage II
Clade F
0.05

D

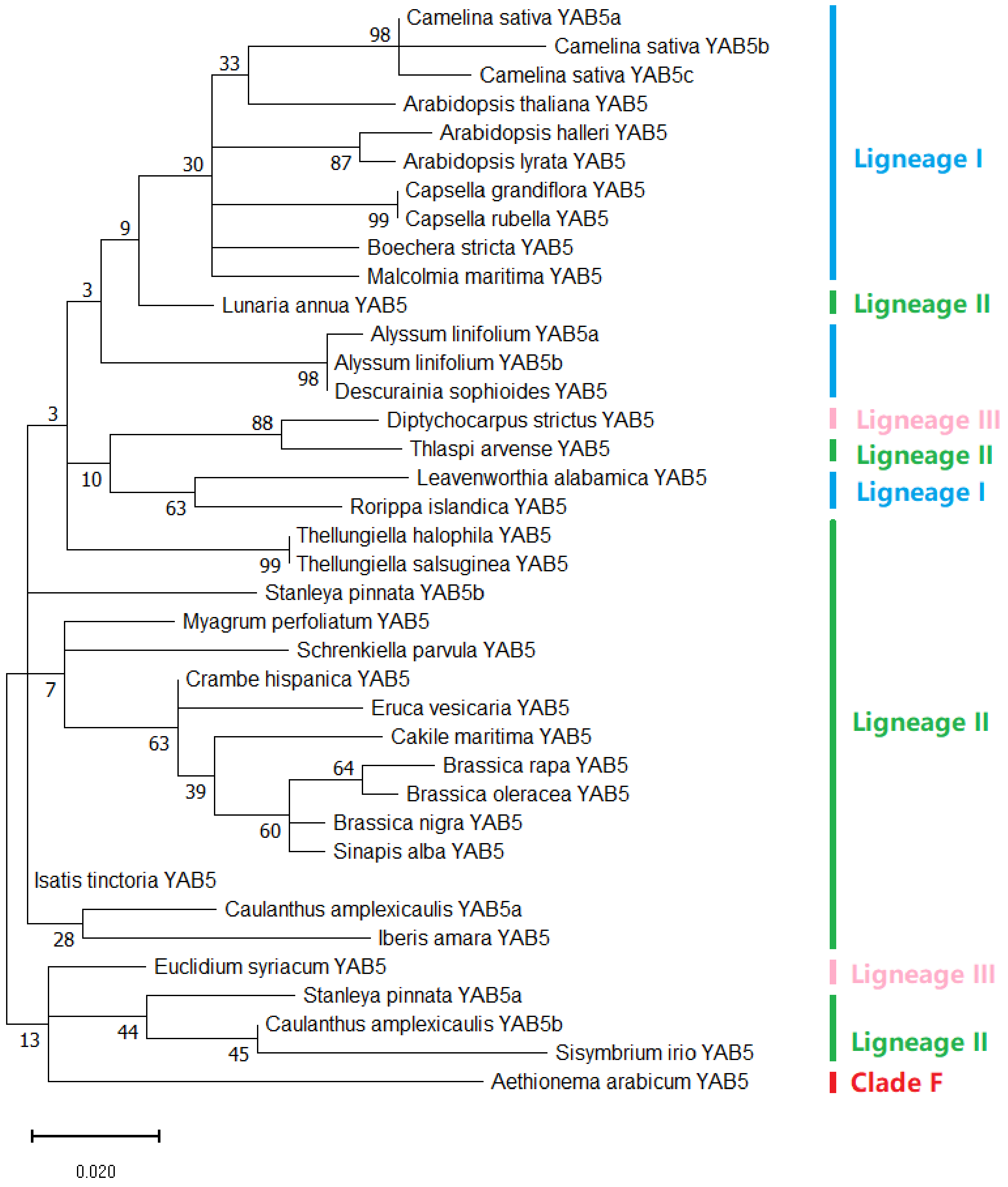
Camelina sativa YAB5a
Camelina sativa YAB5b
Camelina sativa YAB5c
Arabidopsis thaliana YAB5
Arabidopsis halleri YAB5
Arabidopsis lyrata YAB5
Capsella grandiflora YAB5
Capsella rubella YAB5
Boechera stricta YAB5
Malcolmia maritima YAB5
Lunaria annua YAB5
Alyssum linifolium YAB5a
Alyssum linifolium YAB5b
Descurainia sophioides YAB5
Diptychocarpus strictus YAB5
Thlaspi arvense YAB5
Leavenworthia alabamica YAB5
Rorippa islandica YAB5
Thellungiella halophila YAB5
Thellungiella salsuginea YAB5
Stanleya pinnata YAB5b
Myagrum perfoliatum YAB5
Schrenkiella parvula YAB5
Crambe hispanica YAB5
Eruca vesicaria YAB5
Cakile maritima YAB5
Brassica rapa YAB5
Brassica oleracea YAB5
Brassica nigra YAB5
Sinapis alba YAB5
Isatis tinctoria YAB5
Caulanthus amplexicaulis YAB5a
Iberis amara YAB5
Euclidium syriacum YAB5
Stanleya pinnata YAB5a
Caulanthus amplexicaulis YAB5b
Sisymbrium irio YAB5
Aethionema arabicum YAB5
Ligneage I
Ligneage II
Ligneage III
Ligneage II
Ligneage I
Ligneage II
Ligneage III
Ligneage II
Clade F
0.020

E

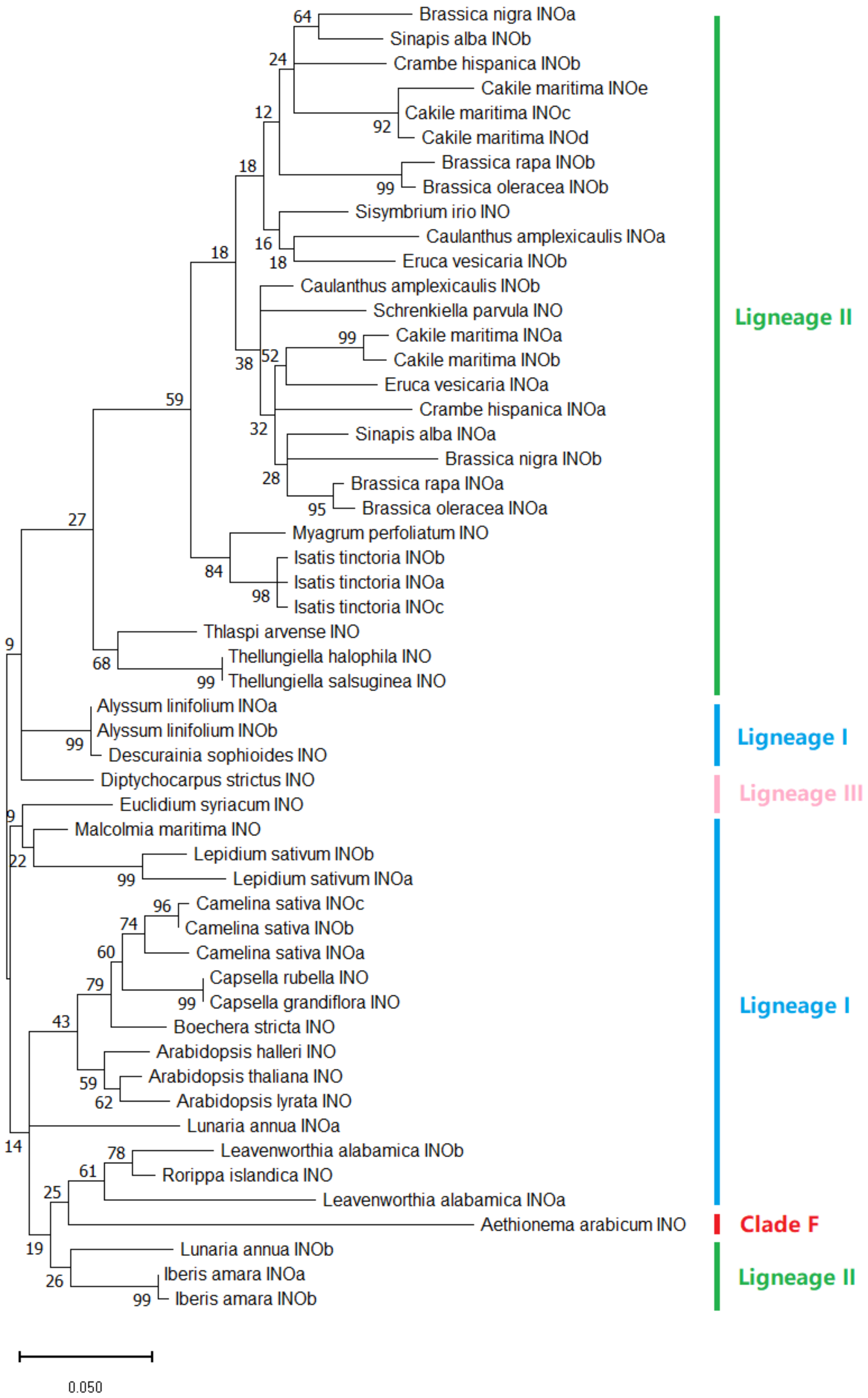
Brassica nigra INOa
Sinapis alba INOb
Crambe hispanica INOb
Cakile maritima INOe
Cakile maritima INOc
Cakile maritima INOd
Brassica rapa INOb
Brassica oleracea INOb
Sisymbrium irio INO
Caulanthus amplexicaulis INOa
Eruca vesicaria INOb
Caulanthus amplexicaulis INOb
Schrenkiella parvula INO
Cakile maritima INOa
Cakile maritima INOb
Eruca vesicaria INOa
Crambe hispanica INOa
Sinapis alba INOa
Brassica nigra INOb
Brassica rapa INOa
Brassica oleracea INOa
Myagrum perfoliatum INO
Isatis tinctoria INOb
Isatis tinctoria INOa
Isatis tinctoria INOc
Thlaspi arvense INO
Thellungiella halophila INO
Thellungiella salsuginea INO
Alyssum linifolium INOa
Alyssum linifolium INOb
Descurainia sophioides INO
Diptychocarpus strictus INO
Euclidium syriacum INO
Malcolmia maritima INO
Lepidium sativum INOb
Lepidium sativum INOa
Camelina sativa INOc
Camelina sativa INOb
Camelina sativa INOa
Capsella rubella INO
Capsella grandiflora INO
Boechera stricta INO
Arabidopsis halleri INO
Arabidopsis thaliana INO
Arabidopsis lyrata INO
Lunaria annua INOa
Leavenworthia alabamica INOb
Rorippa islandica INO
Leavenworthia alabamica INOa
Aethionema arabicum INO
Lunaria annua INOb
Iberis amara INOa
Iberis amara INOb
Ligneage II
Ligneage I
Ligneage III
Ligneage I
Clade F
Ligneage II
0.050

F

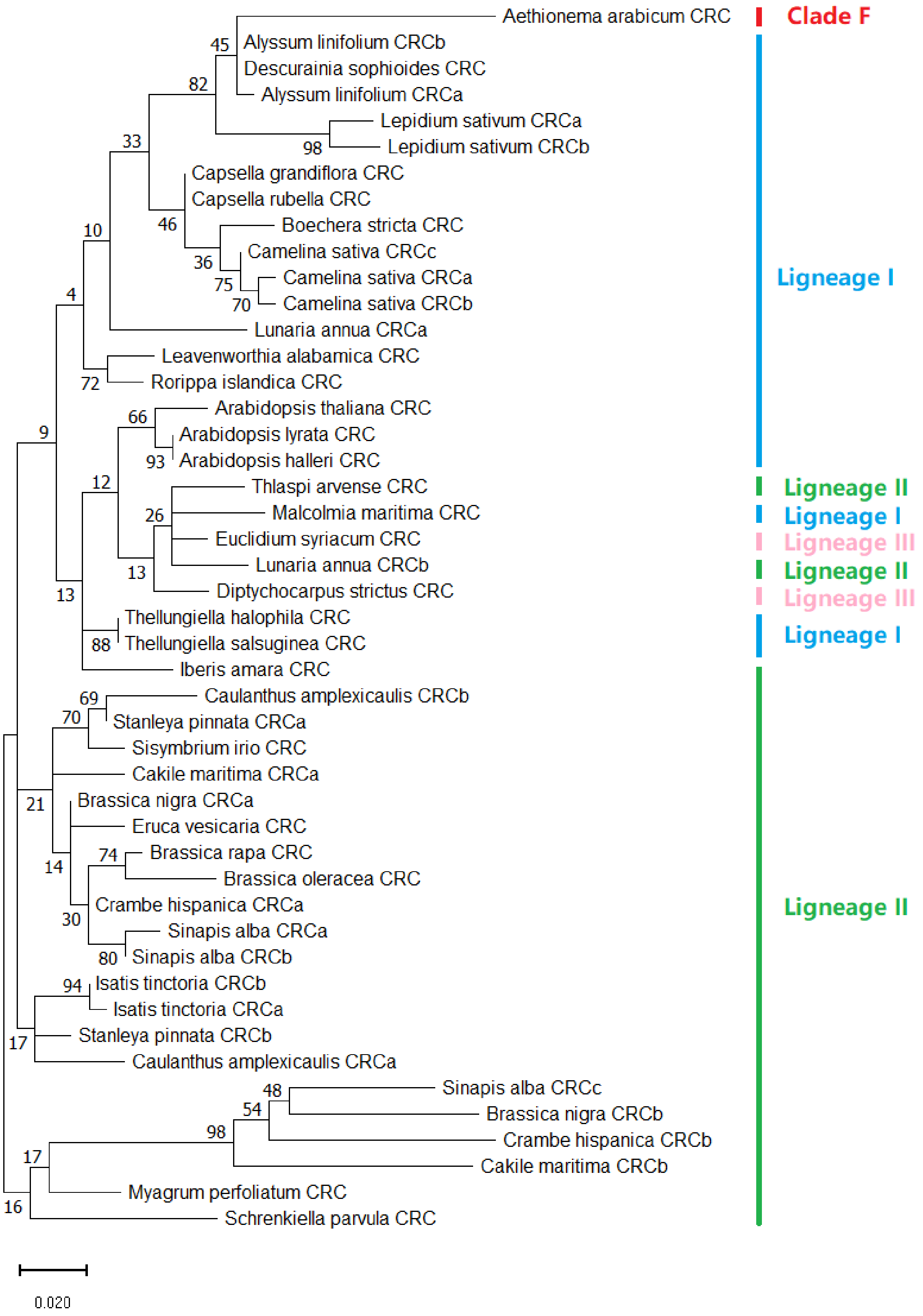
Aethionema arabicum CRC
Alyssum linifolium CRCb
Descurainia sophioides CRC
Alyssum linifolium CRCa
Lepidium sativum CRCa
Lepidium sativum CRCb
Capsella grandiflora CRC
Capsella rubella CRC
Boechera stricta CRC
Camelina sativa CRCc
Camelina sativa CRCa
Camelina sativa CRCb
Lunaria annua CRCa
Leavenworthia alabamica CRC
Rorippa islandica CRC
Arabidopsis thaliana CRC
Arabidopsis lyrata CRC
Arabidopsis halleri CRC
Thlaspi arvense CRC
Malcolmia maritima CRC
Euclidium syriacum CRC
Lunaria annua CRCb
Diptychocarpus strictus CRC
Thellungiella halophila CRC
Thellungiella salsuginea CRC
Iberis amara CRC
Caulanthus amplexicaulis CRCb
Stanleya pinnata CRCa
Sisymbrium irio CRC
Cakile maritima CRCa
Brassica nigra CRCa
Eruca vesicaria CRC
Brassica rapa CRC
Brassica oleracea CRC
Crambe hispanica CRCa
Sinapis alba CRCa
Sinapis alba CRCb
Isatis tinctoria CRCb
Isatis tinctoria CRCa
Stanleya pinnata CRCb
Caulanthus amplexicaulis CRCa
Sinapis alba CRCc
Brassica nigra CRCb
Crambe hispanica CRCb
Cakile maritima CRCb
Myagrum perfoliatum CRC
Schrenkiella parvula CRC
Clade F
Ligneage I
Ligneage II
Ligneage I
Ligneage III
Ligneage II
Ligneage III
Ligneage I
Ligneage II
0.020
