# Supplementary material for: Evolutionary Analysis of the YABBY Gene Family in Brassicaceae
Source: Plants (Basel). 2021 Dec 8;10(12):2700. doi: 10.3390/plants10122700 (PMC8704796; doi:10.3390/plants10122700)
Supplement: Supplementary file 1 [file plants-10-02700-s001.zip › Table S1.pdf]

Table S1 List of the *YABBY* family members identified in 37 Brassicaceae species

Species (No. of haploid chromosomes)	name	Gene ID	Chromosome/scaffold	Start	Stop	Strand	Amino acid length (aa)
Aethionema arabicum (n=11)	*AarFIL*	AA_scaffold4845_51	AA_scaffold4845	228,065	230,503	+	229
	AarYAB2	AA_scaffold2999_2	AA_scaffold2999	6,267	8,327	+	161[a]
	AarYAB3	AA_scaffold1960_115	AA_scaffold1960	471,741	473,828	+	197
	AarYAB5	AA_scaffold3487_39	AA_scaffold3487	189,445	191,441	-	158
	AarINO	AA_scaffold1441_215	AA_scaffold1441	1,012,431	1,014,261	-	238
	AarCRC	AA_scaffold874_23	AA_scaffold874	160,490	161,724	+	181
Alyssum linifolium (n=8)	*AliFIL*	Alyli.0055s0230	Alys_scaffold55	854,111	856,527	-	223
	AliYAB2a	Alyli.0011s0017	Alys_scaffold11	61,661	66,520	-	185
	AliYAB2b	Alyli.0109s0177	Alys_scaffold109	650,948	655,689	+	185
	AliYAB3	Alyli.0058s0163	Alys_scaffold58	612,358	615,540	-	233
	AliYAB5a	Alyli.0166s0028	Alys_scaffold166	349,707	356,286	-	164
	AliYAB5b	Alyli.0390s0001	Alys_scaffold390	53,177	59,606	-	164
	AliINOa	Alyli.0113s0167	Alys_scaffold113	624,729	626,663	-	232
	AliINOb	Alyli.0137s0034	Alys_scaffold137	108,965	110,885	-	232
	AliCRCa	Alyli.0111s0125	Alys_scaffold111	574,288	576,044	+	179
	AliCRCb	Alyli.0272s0002	Alys_scaffold272	984	2,560	-	179
Arabidopsis halleri (n=8)	*AhaFIL*	Araha.8830s0006.1	Scaffold8830	33,705	36,109	+	229
	AhaYAB2	Araha.0425s0007.1	Scaffold425	20,684	24,434	-	185
	AhaYAB3	Araha.27810s0005.1	Scaffold27810	10,905	13,806	+	238
	AhaYAB5	Araha.17793s0001.1	Scaffold17793	2,999	5,258	+	164[a]
	AhaINO	Araha.0889s0003.1	Scaffold889	15,567	17,191	+	231
	AhaCRC	Araha.18871s0006.1	Scaffold18871	22,757	24,113	+	181
Arabidopsis lyrata (n=8)	*AlyFIL*	AL_scaffold_403460.1	scaffold_4	22,017,932	22,020,047	-	229
	AlyYAB2	AL_fgenesh2_kg.1__861__AT1G08465.1	scaffold_1	3,194,335	3,197,807	+	184
	AlyYAB3	AL_fgenesh2_kg.6__3684__AT4G00180.1	scaffold_6	25,007,681	25,010,148	+	240
	AlyYAB5	AL_fgenesh2_kg.4__584__AT2G26580.2	scaffold_4	9,723,125	9,733,336	+	164
	AlyINO	AL_fgenesh1_pg.C_scaffold_1002152	scaffold_1	10,399,364	10,401,011	+	236
	AlyCRC	AL_fgenesh2_kg.2__1172__AT1G69180.1	scaffold_2	13,627,310	13,628,419	-	181
Arabidopsis thaliana (n=5)	*AthFIL*	AT2G45190	Chr2	18,628,265	18,630,712	-	229
	AthYAB2	AT1G08465	Chr1	2,675,813	2,679,781	+	184
	AthYAB3	AT4G00180	Chr4	72,552	75,384	-	240
	AthYAB5	AT2G26580	Chr2	11,303,590	11,306,953	-	164
	AthINO	AT1G23420	Chr1	8,317,396	8,319,483	+	231
	AthCRC	AT1G69180	Chr1	26,007,465	26,009,059	-	181
Boechera Stricta (n=7)	*BstFIL*	Bostr.25993s0255	Scaffold25993	1,263,399	1,265,780	+	224
	BstYAB2	Bostr.25219s0098	Scaffold25219	470,214	474,130	-	188
	BstYAB3	Bostr.14547s0027	Scaffold14547	126,573	129,334	-	235
	BstYAB5	Bostr.22545s0001	Scaffold22545	121,789	127,161	+	164
	BstINO	Bostr.12659s0203	Scaffold12659	1,141,769	1,143,696	+	232
	BstCRC	Bostr.10273s0455	Scaffold10273	2,691,847	2,693,513	+	179
Brassica Rapa (AA, n=10)	*BraFILa*	Bra000378	A03	10,842,756	10,844,864	-	225
	BraFILb	Bra003309	A07	15,810,983	15,813,136	+	210
	BraFILc	Bra040322	A04	18,606,921	18,609,195	-	233
	BraYAB2a	Bra018624	A06	2,930,267	2,934,266	+	170
	BraYAB2b	Bra030728	A08	19,854,846	19,858,624	-	157
	BraYAB2c	Bra031629	A09	37,255,425	37,259,202	-	187
	BraYAB3	Bra037320	A09	54,270	57,129	-	238
	BraYAB5	Bra000538	A03	11,663,078	11,665,462	+	164
	BraINOa	Bra012373	A07	10,359,397	10,361,028	-	235
	BraINOb	Bra024599	A09	25,345,503	25,347,195	-	227
	BraCRC	Bra004364	A07	21,524,923	21,526,172	-	179

Brassica Nigra (BB, n=8)	*BniFILa*	BniB013686	B5	38,918,975	38,921,153	-	233
	BniFILb	BniB019437	B4	25,414,636	25,416,994	-	228
	BniFILc	BniB048967	B3	26,744,492	26,746,669	+	228
	BniYAB2a	BniB034256	B6	16,423,192	16,426,910	+	187
	BniYAB2b	BniB044742	B7	41,092,639	41,096,250	-	188
	BniYAB2c	BniB046889	B4	18,998,659	19,002,500	-	187
	BniYAB3	BniB017304	scaffold_215.1	221,911	224,866	-	234
	BniYAB5	BniB018812	B3	25,463,248	25,465,739	+	163
	BniINOa	BniB004092	B2	7,318,437	7,320,044	+	226
	BniINOb	BniB039665	B7	26,883,978	26,889,162	+	230
	BniCRCa	BniB031980	B7	17,072,800	17,073,978	+	179
	BniCRCb	BniB033605	scaffold_47.1	207,528	208,756	+	173
Brassica Oleracea (CC, n=9)	*BolFILa*	Bol007056	C07	28,857,387	28,859,727	-	228
	BolFILb	Bol021714	C04	40,279,659	40,282,064	-	233
	BolFILc	Bol029536	C03	13,166,787	13,169,537	-	226
	BolYAB2a	Bol006553	C08	39,960,378	39,965,609	-	187
	BolYAB2b	Bol021086	C08	16,624,174	16,627,887	+	188
	BolYAB2c	Bol041214	C05	3,080,240	3,084,020	+	188
	BolYAB3	Bol000755	Scaffold000503	68,157	71,329	-	242
	BolYAB5	Bol032882	C06	6,109,448	6,111,848	+	164
	BolINOa	Bol012288	C06	23,382,715	23,384,323	-	232
	BolINOb	Bol023478	Scaffold000099_P1	165,394	167,322	-	234
	BolCRC	Bol023957	C07	7,655,811	7,657,081	+	179
Brassica Juncea (AABB, n=18)	*BjuFILa*	BjuA010720	A03	13,415,229	13,417,635	-	228
	BjuFILb	BjuA017479	A04	24,768,342	24,770,649	-	233
	BjuFILc	BjuA026645	A07	21,853,808	21,856,118	+	218
	BjuFILd	BjuB016844	B08	26,330,551	26,332,716	-	228
	BjuFILe	BjuB022358	B06	24,804,331	24,806,706	-	228
	BjuFILf	BjuB041886	B01	344,584	346,769	+	233
	BjuFILg	BjuO008433	Contig6259	2,689	5,042	+	225
	BjuYAB2a	BjuA021585	A06	3,250,216	3,253,951	+	189
	BjuYAB2b	BjuA031221	A08	24,945,122	24,948,796	-	188
	BjuYAB2c	BjuB033429	B03	1,077,197	1,080,842	+	188
	BjuYAB2d	-	B04	19,233,085	19,236,826	-	187
	BjuYAB2e	-	A09	54,184,395	54,188,141	-	187
	BjuYAB2f	-	B06	17,344,965	17,348,806	-	187
	BjuYAB2g	-	A08	25,006,581	25,010,329	-	187
	BjuYAB3a	BjuA031379	A09	70,316	73,014	-	238
	BjuYAB3b	-	B08	24,203,618	24,206,380	-	238
	BjuYAB5a	BjuA010870	A03	14,379,092	14,381,498	+	164
	BjuYAB5b	BjuB041316	B08	29,908,953	29,911,448	-	164
	BjuINOa	BjuA014857	A09	39,397,854	39,399,529	-	216
	BjuINOb	BjuA025981	A07	15,733,737	15,735,365	-	232
	BjuINOc	BjuB017830	B03	18,748,159	18,749,783	+	232
	BjuINOd	BjuB031333	B03	20,147,377	20,148,997	+	230
	BjuCRCa	BjuB035930	B03	32,099,588	32,100,602	-	172
	BjuCRCb	BjuO001705	Contig137_156605_433750	75,945	77,146	-	174
	BjuCRCc	-	A07	29,930,268	29,931,243	-	139[b]
Brassica Napus (AACC, n=19)	*BnaFILa*	GSBRNA2T00018014001	chrA07_random	1,409,138	1,411,609	-	218
	BnaFILb	GSBRNA2T00025227001	chrAnn_random	45,762,354	45,764,160	+	233[c]
	BnaFILc	GSBRNA2T00037339001	chrC04	47,923,766	47,926,564	-	233
	BnaFILd	GSBRNA2T00101516001	chrC03	14,139,010	14,141,834	-	226
	BnaFILe	GSBRNA2T00105785001	chrC06	19,201,254	19,203,739	+	228
	BnaFILf	GSBRNA2T00138963001	chrA03	9,994,230	9,996,415	-	225
	BnaYAB2a	GSBRNA2T00019352001	chrC08	37,006,148	37,011,264	-	188
	BnaYAB2b	GSBRNA2T00047230001	chrA08	18,091,254	18,092,650	-	188[d]
	BnaYAB2c	GSBRNA2T00100579001	chrC05	3,018,924	3,023,868	+	188
	BnaYAB2d	GSBRNA2T00104691001	chrC08	18,669,192	18,673,532	+	189
	BnaYAB2e	GSBRNA2T00122244001	chrA09	32,644,170	32,649,965	-	188
	BnaYAB2f	GSBRNA2T00156238001	chrA06	2,824,327	2,828,557	+	190
	BnaYAB3a	GSBRNA2T00037885001	chrAnn_random	19,824,148	19,829,307	+	239[e]

	BnaYAB3b	GSBRNA2T00037886001	chrAnn_random	19,829,376	19,833,331	+	238
	BnaYAB5a	GSBRNA2T00066168001	chrC03	15,258,021	15,261,107	+	164
	BnaYAB5b	GSBRNA2T00138788001	chrA03	10,777,785	10,780,718	+	164
	BnaINOa	GSBRNA2T00033187001	chrA07	9,576,163	9,578,141	-	235
	BnaINOb	GSBRNA2T00061772001	chrC07	18,603,703	18,605,366	-	235
	BnaINOc	GSBRNA2T00079608001	chrA09	23,077,021	23,078,779	-	233
	BnaINOd	GSBRNA2T00119349001	chrC05	12,428,883	12,430,852	+	234
	BnaCRCa	GSBRNA2T00082021001	chrCnn_random	61,294,766	61,295,114	-	106[f]
	BnaCRCb	GSBRNA2T00101975001	chrA07	20,144,971	20,146,565	-	180
Brassica carinata (BBCC, n=17)	*BcaFILa*	Bca52824-003283	chrB01	52439352	52441530	+	228
	BcaFILb	Bca52824-007563	chrB02	8744537	8746889	-	228
	BcaFILc	Bca52824-042620	chrC01	58261909	58264671	+	226
	BcaFILd	Bca52824-082352	chrC08	28864562	28866980	+	228
	BcaYAB2a	Bca52824-013330	chrB03	9556733	9560414	-	189
	BcaYAB2b	Bca52824-034089	chrB07	52461177	52468577	+	188
	BcaYAB2c	Bca52824-075862	chrC07	24555840	24559735	+	189
	BcaYAB2d	Bca52824-079412	chrC07	50796350	50825424	-	174
	BcaYAB3a	Bca52824-002223	chrB01	40991712	40994465	-	238
	BcaYAB3b	Bca52824-061666	chrC04	65349335	65352146	+	234
	BnaINOa	Bca52824-014148	chrB03	15439793	15441411	-	230
	BnaINOb	Bca52824-032339	chrB07	18284786	18286392		226
	BnaINOc	Bca52824-063730	chrC05	15456208	15457893	+	234
	BnaINOd	Bca52824-072505	chrC06	34271881	34273515	+	232
	BcaYAB5	Bca52824-003026	chrB01	49761875	49764369	-	164
	Bcayab5	Bca52824_	chrC01	56073494	56074056	-	25**
	BcaCRC	Bca52824-015877	chrB03	29592512	29593873	-	204
Cakile maritima (n=9)	*CmaFILa*	Camar.1371s0002	Caki_scaffold1371	16,582	18,848	+	227
	CmaFILb	Camar.5487s0001	Caki_scaffold5487	9,107	12,523	-	228
	CmaFILc	Camar.0753s0010	Caki_scaffold753	67,579	70,391	+	234
	CmaYAB2a	Camar.0034s0121	Caki_scaffold34	500,077	504,638	-	189
	CmaYAB2b	Camar.4745s0001	Caki_scaffold4745	121	4,325	+	187
	CmaYAB2c	Camar.0004s0074	Caki_scaffold4	296,954	301,173	-	188
	CmaYAB3a	Camar.0666s0033	Caki_scaffold666	147,237	150,131	-	237
	CmaYAB3b	Camar.8579s0002	Caki_scaffold8579	9,025	10,847	+	190[a]
	CmaYAB5	Camar.2950s0001	Caki_scaffold2950	686	13,208	-	143[a]
	CamINOa	Camar.0096s0018	Caki_scaffold96	77,251	78,923	+	233
	CamINOb	Camar.12454s0001	Caki_scaffold12454	82	1774	+	227
	CamINOc	Camar.1627s0006	Caki_scaffold1627	27,515	29,316	-	252
	CamINOd	Camar.1627s0007	Caki_scaffold1627	32,107	32,812	-	149[g]
	CamINOe	Camar.9939s0002	Caki_scaffold9939	5,541	6,134	+	141[h]
	CamCRCa	Camar.3001s0004	Caki_scaffold3001	34,470	36,067	-	179
	CamCRCb	Camar.0776s0009	Caki_scaffold776	46,637	48,045	-	178
Camelina Sativa (n=18)	*CsaFILa*	Csa04g063090	Chr4	28,568,895	28,570,959	-	227
	CsaFILb	Csa05g004070	Chr5	1,475,883	1,478,377	+	228
	CsaFILc	Csa06g051580	Chr6	24,884,693	24,886,757	-	227
	CsaYAB2a	Csa03g011910	Chr3	3,545,638	3,549,605	+	188
	CsaYAB2b	Csa14g009930	Chr14	3,614,475	3,618,880	+	188
	CsaYAB2c	Csa17g011910	Chr17	3,414,978	3,425,469	+	174
	CsaYAB3a	Csa02g001230	Chr2	138,356	140,935	-	203[a]
	CsaYAB3b	Csa08g001240	Chr8	120,699	123,015	-	236
	CsaYAB3c	Csa13g057240	Chr13	23,939,495	23,942,633	+	235
	CsaYAB5a	Csa05g044630	Chr5	16,973,038	16,976,320	+	164
	CsaYAB5b	Csa07g060160	Chr7	30,430,064	30,432,998	-	164
	CsaYAB5c	Csa16g050800	Chr16	26,033,745	26,036,436	-	164
	CsaINOa	Csa03g027340	Chr3	10,834,379	10,835,999	+	232
	CsaINOb	Csa14g030690	Chr14	11,740,213	11,741,751	+	232
	CsaINOc	Csa17g030590	Chr17	11,457,055	11,458,636	+	232
	CsaCRCa	Csa05g085860	Chr5	30,871,976	30,873,206	-	179
	CsaCRCb	Csa07g035840	Chr7	19,298,617	19,299,821	-	179
	CsaCRCc	Csa16g030480	Chr16	16,142,532	16,143,777	-	180
Capsella	*CgrFIL*	Cagra.0239s0072.1	Scaffold239	255,792	258,174	-	227

Grandiflora (n=8)	*CgrYAB2*	Cagra.4395s0036.1	Scaffold4395	144,793	148,330	+	188
	CgrYAB3	Cagra.1261s0010.1	Scaffold1261	30,818	33,537	-	242
	CgrYAB5	Cagra.11302s0003.1	Scaffold11302	14,670	18,381	-	164
	CgrINO	Cagra.0605s0067.1	Scaffold605	201,780	203,379	-	235
	CgrCRC	Cagra.0537s0004.1	Scaffold537	17,627	18,837	-	179
Capsella Rubella (n=8)	*CruFIL*	Carubv10024011m	Sca4	13,855,759	13,858,066	-	224
	CruYAB2	Carubv10010384m	Sca1	2,754,758	2,758,838	+	188
	CruYAB3	Carubv10001833m	Sca6	16,531,658	16,535,150	+	242
	CruYAB5	Carubv10024208m	Sca4	4,367,459	4,371,209	+	164
	CruINO	Carubv10012024m	Sca1	8,295,346	8,296,939	+	235
	CruCRC	Carubv10021641m	Sca2	9,410,902	9,412,169	-	179
Caulanthus amplexicaulis (n=14)	*CamFILa*	Caamp.1031s0684	Caul_scaffold1031	3,010,381	3,013,049	-	226
	CamFILb	Caamp.0018s0100	Caul_scaffold18	406,105	408,570	-	228
	CamYAB2a	Caamp.0052s0070	Caul_scaffold52	276,237	280,760	-	189
	CamYAB2b	Caamp.0188s0033	Caul_scaffold188	118,553	122,760	+	187
	CamYAB3a	Caamp.1037s0019	Caul_scaffold1037	78,355	81,067	-	236
	CamYAB3b	Caamp.1040s0023	Caul_scaffold1040	89,923	92,976	-	218
	CamYAB5a	Caamp.0039s0028	Caul_scaffold39	536,790	540,585	-	164
	CamYAB5b	Caamp.0017s0045	Caul_scaffold17	931,309	936,578	-	164
	CamINOa	Caamp.1033s0170	Caul_scaffold1033	804,594	806,273	+	235
	CamINOb	Caamp.0037s0454	Caul_scaffold37	1,938,909	1,940,584	+	233
	CamCRCa	Caamp.0303s0993	Caul_scaffold303	4,427,891	4,429,152	+	179
	CamCRCb	Caamp.1036s0510	Caul_scaffold1036	2,279,392	2,280,963	+	179
Crambe hispanica (n=15)	*ChiFILa*	Crahi.0058s0065	Cram_scaffold58	725,877	728,424	-	224
	ChiFILb	Crahi.0004s0131	Cram_scaffold4	624,267	626850	+	233
	ChiFILc	Crahi.0984s0022	Cram_scaffold984	139,594	142,037	-	228
	ChiYAB2a	Crahi.0189s0071	Cram_scaffold189	270,257	274,524	-	188
	ChiYAB2b	Crahi.0007s0219	Cram_scaffold7	825,840	830,026	+	187
	ChiYAB2c	Crahi.0003s0216	Cram_scaffold3	958,838	963,063	-	187
	ChiYAB3	Crahi.0025s0147	Cram_scaffold25	844,091	847,196	+	237
	ChiYAB5	Crahi.1116s0001	Cram_scaffold1116	12,115	15,838	-	164
	ChiINOa	Crahi.0507s0019	Cram_scaffold507	155,192	156,833	+	232
	ChiINOb	Crahi.1053s0004	Cram_scaffold1053	52,377	52,377	+	228
	ChiCRCa	Crahi.0577s0011	Cram_scaffold577	99,259	100,951	-	179
	ChiCRCb	Crahi.1382s0006	Cram_scaffold1382	69,402	70,601	-	175
Descurainia sophioides (n=7)	*DsoFIL*	Desop.0219s0009	Desc_scaffold219	29,528	31,924	-	223
	DsoYAB2	Desop.0203s0077	Desc_scaffold203	298,913	303,287	+	185
	DsoYAB3	Desop.0222s0495	Desc_scaffold222	1,956,838	1,959,852	-	233
	DsoYAB5	Desop.0209s0235	Desc_scaffold209	1,555,477	1,562,313	+	164
	DsoINO	Desop.0021s0072	Desc_scaffold21	255,743	257,410	-	232
	DsoCRC	Desop.0206s0503	Desc_scaffold206	2,156,037	2,157,548	+	179
Diptychocarpus strictus (n=7)	*DstFIL*	Distr.0015s0383	Dipt_scaffold15	2,014,241	2,016,828	+	240
	DstYAB2	Distr.0254s0628	Dipt_scaffold254	3,401,127	3,405,025	-	197
	DstYAB3	Distr.0019s0020	Dipt_scaffold19	67,000	69,281	-	257
	DstYAB5	Distr.0261s0816	Dipt_scaffold261	5,334,583	5,338,026	-	163
	DstINO	Distr.0035s0539	Dipt_scaffold35	3,418,324	3,419,845	-	218
	DstCRC	Distr.0260s0491	Dipt_scaffold260	3,916,079	3,917,348	-	177
Eruca vesicaria (n=11)	*EveFILa*	Eruve.1903s0016	Eruc_scaffold1903	87,090	89,528	+	228
	EveFILb	Eruve.0665s0002	Eruc_scaffold665	5,696	7,717	+	217[i]
	EveFILc	Eruve.0140s0003	Eruc_scaffold140	63,768	66,285	-	233
	EveFILd	Eruve.7673s0001	Eruc_scaffold7673	1,448	3,775	-	228
	EveFILe	Eruve.1226s0001	Eruc_scaffold1226	3,722	6,224	+	228
	EveYAB2a	Eruve.7302s0003	Eruc_scaffold7302	12,523	16,401	-	189
	EveYAB2b	Eruve.0022s0027	Eruc_scaffold22	118,924	123,345	-	189
	EveYAB2c	Eruve.1111s0008	Eruc_scaffold1111	34,759	39,121	-	188
	EveYAB2d	Eruve.0130s0049	Eruc_scaffold130	204,620	208,653	-	187
	EveYAB2e	Eruve.0155s0050	Eruc_scaffold155	289,367	294,816	+	187
	EveYAB2f	Eruve.3702s0005	Eruc_scaffold3702	27,073	31,913	+	188
	EveYAB3a	Eruve.1065s0020	Eruc_scaffold1065	121,482	124,559	+	235
	EveYAB3b	Eruve.4375s0001	Eruc_scaffold4375	7,015	11,767	-	103[a]
	EveYAB5	Eruve.0979s0006	Eruc_scaffold979	143,744	148,847	+	164

	EveINOa	Eruve.0052s0080	Eruc_scaffold52	393,340	395,248	+	236
	EveINOb	Eruve.0510s0016	Eruc_scaffold510	88,972	90,660	-	235
	EveCRC	Eruve.0230s0001	Eruc_scaffold230	5,137	6,814	+	179
Euclidium syriacum (n=7)	*EsyFIL*	Eusyr.0123s0351	Eucl_scaffold123	1,619,342	1,622,025	-	232
	EsyYAB2	Eusyr.0027s0277	Eucl_scaffold27	1,417,052	1,421,965	-	189
	EsyYAB3	Eusyr.0022s0547	Eucl_scaffold22	2,370,416	2,373,124	+	255
	EsyYAB5	Eusyr.0134s0184	Eucl_scaffold134	885,377	890,325	-	162
	EsyINO	Eusyr.0009s0365	Eucl_scaffold9	3,009,838	3,011,724	+	232
	EsyCRC	Eusyr.0118s0152	Eucl_scaffold118	993,263	994,870	-	180
Iberis amara (n=7)	*IamFILa*	Ibeam.1092s0001	Iber_scaffold1092	5,894	8,762	-	228
	IamFILb	Ibeam.2626s0006	Iber_scaffold2626	31,030	33,614	-	222
	IamYAB2a	Ibeam.1280s0011	Iber_scaffold1280	85,993	91,127	-	192
	IamYAB2b	Ibeam.6080s0004	Iber_scaffold6080	25,072	31,060	+	190
	IamYAB2c	Ibeam.4957s0008	Iber_scaffold4957	43,737	46,383	-	170[a]
	IamYAB3a	Ibeam.0485s0001	Iber_scaffold485	44,031	50,486	+	203
	IamYAB3b	Ibeam.0217s0042	Iber_scaffold217	200,432	203,217	+	227
	IamYAB5	Ibeam.5072s0001	Iber_scaffold5072	5,795	9,298	-	161
	IamINOa	Ibeam.3194s0008	Iber_scaffold3194	50,804	52,525	-	232
	IamINOb	Ibeam.6547s0003	Iber_scaffold6547	18,931	20,647	-	232
	IamCRC	Ibeam.0421s0006	Iber_scaffold421	92,177	93,662	-	179
Isatis tinctoria (n=14)	*ItiFILa*	Isati.1522s0001	Isat_scaffold1522	905	3,192	-	238
	ItiFILb	Isati.0970s0016	Isat_scaffold970	72,472	74,871	-	238
	ItiFILc	Isati.2901s0010	Isat_scaffold2901	49,856	52,327	-	238
	ItiFILd	Isati.0251s0020	Isat_scaffold251	82,782	85,253	+	238
	ItiYAB2a	Isati.5796s0006	Isat_scaffold5796	15,298	17,790	-	185
	ItiYAB2b	Isati.0242s0027	Isat_scaffold242	117,586	119,158	-	165[a]
	ItiYAB3a	Isati.3543s0007	Isat_scaffold3543	28,235	30,404	+	103[j]
	ItiYAB3b	Isati.5191s0004	Isat_scaffold5191	28,254	30,695	+	140[a]
	ItiYAB3c	Isati.5387s0001	Isat_scaffold5387	622	895	+	50[a]
	ItiYAB5	Isati.2783s0004	Isat_scaffold2783	51,827	56,627	-	164
	ItiINOa	Isati.11780s0001	Isat_scaffold11780	1,498	3,126	-	234
	ItiINOb	Isati.1919s0011	Isat_scaffold1919	27,811	29,444	-	235
	ItiINOc	Isati.2755s0001	Isat_scaffold2755	16,159	17,784	+	234
	ItiCRCa	Isati.2627s0004	Isat_scaffold2627	15,964	17,178	+	179
	ItiCRCb	Isati.0829s0003	Isat_scaffold829	14,627	15,846	-	179
Leavenworthia alabamica (n=11)	*LalFILa*	LA_scaffold1025_8	LA_scaffold1025	30,017	32,036	+	225
	LalFILb	LA_scaffold450_30	LA_scaffold450	122,688	124,681	+	227
	LalYAB2	LA_scaffold1507_52	LA_scaffold1507	186,182	189,006	-	184
	LalYAB5	LA_scaffold1966_1	LA_scaffold1966	1,331	4,484	-	165
	LalINOa	LA_scaffold1549_30	LA_scaffold1549	136,573	140,635	-	197[a]
	LalINOb	LA_scaffold2022_23	LA_scaffold2022	97,118	98,723	+	230
	LalCRC	LA_scaffold1695_18	LA_scaffold1695	69,479	70,588	-	182
Lepidium sativum (n=12)	*LsaFILa*	Lesat.0020s0455	Lepi_scaffold20	2,118,743	2,120,992	-	226
	LsaFILb	Lesat.0017s0176	Lepi_scaffold17	1,215,141	1,217,307	-	226
	LsaYAB2a	Lesat.0044s0002	Lepi_scaffold44	13,824	19,187	+	185
	LsaYAB2b	Lesat.0112s0862	Lepi_scaffold112	5,095,603	5,100,856	+	184
	LsaYAB3a	Lesat.0298s0020	Lepi_scaffold298	66,007	68,269	-	215
	LsaYAB3b	Lesat.0070s1033	Lepi_scaffold70	8,277,898	8,280,351	+	226
	LsaINOa	Lesat.0018s0454	Lepi_scaffold18	3,534,422	3,536,104	-	231
	LsaINOb	Lesat.0158s0026	Lepi_scaffold158	258,001	259,650	-	230
	LsaCRCa	Lesat.0080s0138	Lepi_scaffold80	767,452	768,947	+	181
	LsaCRCb	Lesat.0101s0554	Lepi_scaffold101	5,155,302	5,156,797	-	184
Lunaria annua (n=15)	*LanFILa*	Luann.0125s0105	Luna_scaffold125	461,509	463,883	-	225
	LanFILb	Luann.0011s0460	Luna_scaffold11	1,738,674	1,740,906	-	225
	LanFILc	Luann.0175s0077	Luna_scaffold175	355,577	357,859	-	225
	LanYAB2a	Luann.0179s0033	Luna_scaffold179	220,708	224,906	+	188
	LanYAB2b	Luann.0449s0001	Luna_scaffold449	1,410	5,080	-	189
	LanYAB3a	Luann.0132s0019	Luna_scaffold132	88,751	90,667	-	225
	LanYAB3b	Luann.0459s0011	Luna_scaffold459	100,478	103,495	-	239
	LanYAB5	Luann.0044s0161	Luna_scaffold44	937,131	941,183	-	164
	LanINOa	Luann.0033s0419	Luna_scaffold33	1,677,648	1,679,362	-	235

	LanINOb	Luann.0062s0082	Luna_scaffold62	321,881	323,591	-	224
	LanCRCa	Luann.0030s0056	Luna_scaffold30	251,854	253,309	+	162[k]
	LanCRCb	Luann.0149s0053	Luna_scaffold149	287,395	288,493	-	170[k]
Malcolmia maritima (n=8)	*MmaFIL*	Mamar.0006s0086	Malc_scaffold6	342,367	344,823	-	227
	MmaYAB2	Mamar.0052s0119	Malc_scaffold52	434,845	439,392	-	184
	MmaYAB3	Mamar.0100s0320	Malc_scaffold100	1,293,713	1,297,082	+	239
	MmaYAB5	Mamar.0184s0006	Malc_scaffold184	139,236	143,416	+	165
	MmaINO	Mamar.0087s0210	Malc_scaffold87	893,850	895,442	+	230
	MmaCRC	Mamar.0084s0069	Malc_scaffold84	308,331	309,990	+	180
Myagrum perfoliatum (n=7)	*MpeFIL*	Myper.0013s0288	Myag_scaffold13	1,134,224	1,136,705	+	238
	MpeYAB2	Myper.0024s0064	Myag_scaffold24	258,194	262,452	+	187
	MpeYAB3	Myper.0035s0018	Myag_scaffold35	57,918	61,138	-	235
	MpeYAB5	Myper.0163s0003	Myag_scaffold163	10,954	15,809	+	164
	MpeINO	Myper.0063s0303	Myag_scaffold63	1,284,690	1,286,337	-	233
	MpeCRC	Myper.0132s1166	Myag_scaffold132	5,441,458	5,442,660	+	179
Rorippa islandica (n=8)	*RisFIL*	Roisl.0028s0065	Rori_scaffold28	261,146	263,542	+	227
	RisYAB2	Roisl.0091s1133	Rori_scaffold91	5,104,047	5,108,380	+	184
	RisYAB3	Roisl.0015s0030	Rori_scaffold15	125,703	128,672	-	233
	RisYAB5	Roisl.0215s0011	Rori_scaffold215	817,752	823,865	-	164
	RisINO	Roisl.0191s0182	Rori_scaffold191	814,514	816,720	+	231
	RisCRC	Roisl.0019s0628	Rori_scaffold19	3,255,854	3,257,455	+	180
Schrenkiella parvula (n=7)	*SpaFIL*	c0004_00279	Sp4	7,003,508	7,005,399	+	227
	SpaYAB2	c0001_00610	Sp1	2,454,121	2,455,370	+	177[l]
	SpaYAB3	c0011_00022	Sp6	72,173	74,600	-	237
	SpaYAB5	c0025_00112	Sp4	5,658,268	5,661,543	-	165
	SpaINO	c0001_01846	Sp1	7,694,639	7,696,275	+	227
	SpaCRC	c0012_00229	Sp5	11,593,959	11,595,252	-	179
Sinapis alba (n=12)	*SalFILa*	Sialb.1382s0006	Sina_scaffold1382	19,265	21,773	-	233
	SalFILb	Sialb.0045s0077	Sina_scaffold45	368,551	371,039	+	228
	SalFILc	Sialb.0034s0116	Sina_scaffold34	496,346	498,796	+	223
	SalYAB2a	Sialb.0085s0054	Sina_scaffold85	177,164	181,375	+	187
	SalYAB2b	Sialb.0020s0049	Sina_scaffold20	206,955	211,242	-	188
	SalYAB2c	Sialb.0501s0005	Sina_scaffold501	18,463	22,845	+	188
	SalYAB3	Sialb.1410s0006	Sina_scaffold1410	35,340	38,756	-	235
	SalYAB5	Sialb.0194s0022	Sina_scaffold194	601,408	605,089	-	164
	SalINOa	Sialb.0089s0100	Sina_scaffold89	549,960	551,569	+	232
	SalINOb	Sialb.0807s0015	Sina_scaffold807	57,800	59,717	-	231
	SalCRCa	Sialb.1064s0015	Sina_scaffold1064	70,125	71,741	+	180
	SalCRCb	Sialb.0848s0009	Sina_scaffold848	63,495	65,471	-	180
	SalCRCc	Sialb.0312s0027	Sina_scaffold312	177,302	179,094	+	178
Sisymbrium irio (n=7)	*SirFIL*	SI_scaffold1717_65	SI_scaffold1717	295,488	297,593	-	228
	SirYAB2a	SI_scaffold1384_35	SI_scaffold1384	146,957	151,034	+	187
	SirYAB2b	SI_scaffold1384_38	SI_scaffold1384	158,842	162,683	+	187
	SirYAB3	SI_scaffold2065_147	SI_scaffold2065	660,012	662,559	+	234
	SirYAB5	SI_scaffold889_15	SI_scaffold889	93,353	94,269	-	64[a]
	SirINO	SI_scaffold1556_73	SI_scaffold1556	345,868	347,539	+	230
	SirCRC	SI_scaffold2962_45	SI_scaffold2962	198,519	199,892	+	172
Stanleya pinnata (n=14)	*SpiYAB3*	Stapi.1881s0001	Stan_scaffold1881	2	1,809	+	160[m]
	SpiYAB5a	Stapi.0024s0015	Stan_scaffold24	475,127	480,128	-	164
	SpiYAB5b	Stapi.0299s0001	Stan_scaffold299	18,796	28,487	-	161
	SpiCRCa	Stapi.0752s0012	Stan_scaffold752	160,162	161,371	+	177
	SpiCRCb	Stapi.7281s0001	Stan_scaffold7281	12,469	13,691	+	176
Thellungiella Halophile (n=7)	*ThaFIL*	Thhalv10001617m	scaffold_22	739,732	742,355	-	229
	ThaYAB2	Thhalv10008858m	scaffold_5	12,910,772	12,915,017	-	189
	ThaYAB3	Thhalv10028925m	scaffold_3	84,014	87,086	-	237
	ThaYAB5	Thhalv10017320m	scaffold_10	541,740	545,537	+	164
	ThaINO	Thhalv10008669m	scaffold_5	7,113,497	7,115,554	-	231
	ThaCRC	Thhalv10019126m	scaffold_9	5,321,944	5,324,683	+	214
Thellungiella Salsuginea (n=7)	*TsaFIL*	Tsa4g30660	ch4-82	2,415,182	2,417,838	-	229
	TsaYAB2	Tsa1g07240	ch1-01	2,753,066	2,757,670	+	189
	TsaYAB3	Tsa6g00210	ch6-01	85,755	89,140	-	237

	TsaYAB5	Tsa4g09600	ch4-58	109,464	113,469	-	164
	TsaINO	Tsa1g20990	ch1-02	1,693,095	1,695,345	+	231
	TsaCRC	Tsa5g26460	ch5-76	139,540	141,215	-	180
Thlaspi arvense (n=7)	*TarFIL*	Thlar.0014s0810	Thla_scaffold14	3,731,062	3,733,395	+	228
	TarYAB2	Thlar.0006s0532	Thla_scaffold6	1,903,061	1,907,240	+	188
	TarYAB3	Thlar.0018s0022	Thla_scaffold18	121,265	124,117	-	237
	TarYAB5	Thlar.0260s0006	Thla_scaffold260	94,900	98,917	-	161
	TarINO	Thlar.0015s0337	Thla_scaffold15	1,448,057	1,449,983	+	234
	TarCRC	Thlar.0346s0025	Thla_scaffold346	144,021	145,600	-	180

[a] partial sequence due to incomplete sequencing data; [b] missing the first exon due to sequence deletion; [c] partial sequence improved by using the NCBI sequence XM_013811248.2; [d] partial sequence improved by using the NCBI sequence XM_013802157.2; [e] partial sequence improved by using the NCBI sequence XM_013838179.2; [f] partial sequence improved by the NCBI sequence EV163186.1; [g] truncated by a stop codon; [h] missing the 4th exon by deletion of internal sequence; [i] incomplete sequence of the first exon due to deletion; [j] missing the first three exons due to sequence deletion; [k] missing the last exon due to deletion; [l] missing the first exon due to its translocation to the chromosome Sp7; [m] imperfect YABBY domain due to sequence deletion.
