# Supplementary material for: Evolutionary Analysis of the YABBY Gene Family in Brassicaceae
Source: Plants (Basel). 2021 Dec 8;10(12):2700. doi: 10.3390/plants10122700 (PMC8704796; doi:10.3390/plants10122700)
Supplement: Supplementary file 1 [file plants-10-02700-s001.zip › Table S2.pdf]

Table S2 Syntenic relationships between *YABBY* genes of different Brassicaceae species.

Species (genome)	Subgenome	tPCK1 Block A	tPCK1 Block B	tPCK6 Block E	tPCK3 Block I	tPCK3 Block J	tPCK5 Block O	Undetermined
Aethionema arabicum		-	*AarINO*	-	*AarYAB5*	*AarFIL*	-	*AarYAB2, AarCRC, AarYAB3*
Arabidopsis halleri		*AhaYAB2*	*AhaINO*	*AhaCRC*	-	*AhaFIL*	*AhaYAB3*	*AhaYAB5*
Arabidopsis lyrata		*AlyYAB2*	*AlyINO*	*AlyCRC*	*AlyYAB5*	*ALyFIL*	*AlyYAB3*	
Arabidopsis thaliana		*AthYAB2*	*AthINO*	*AThCRC*	*AthYAB5*	*AthFIL*	*AthYAB3*	
Boechera stricta		*BstYAB2*	*BstINO*	*BstCRC*	-	*BstFIL*	*BstYAB3*	*BstYAB5*
	LF	*CsaYAB2c*	*CsaINOc*	*CsaCRCc*	*CsaYAB5c*	*CsaFILa*	*CsaYAB3c*	
Camelina sativa	MF1	*CsaYAB2b*	*CsaINOb*	*CsaCRCb*	*CsaYAB5b*	*CsaFILc*	*CsaYAB3b*	
	MF2	*CsaYAB2a*	*CsaINOa*	*CsaCRCa*	*CsaYAB5c*	*CsaFILb*	*CsaYAB3a*	
Capsella grandiflora		*CgrYAB2*	*CgrINO*	*CgrCRC*	-	*CgrFIL*	*CgrYAB3*	*CgrYAB5*
Capsella rubella		*CruYAB2*	*CruINO*	*CruCRC*	*CruYAB5*	*CruFIL*	*CruYAB3*	
Leavenworthia alabamica		*LalYAB2*	*LalINOb*	*LalCRC*	-	*LalFILb*	-	*LalFILa, LalYAB3, LalINOa*
Schrenkiella parvula		*SpaYAB2*	*SpaINO*	*SpaCRC*	*SpaYAB5*	*SpaFIL*	*SpaYAB3*	
Sisymbrium irio		*SirYAB2a, SirYAB2b*	*SirINO*	*SirCRC*	-	*SirFIL*	*SirYAB3*	*SirYAB5*
Thellungiella halophila		*ThaYAB2*	*ThaINO*	*ThaCRC*	*ThaYAB5*	*ThaFIL*	*ThaYAB3*	
Thellungiella salsuginea		*TsaYAB2*	*TsaINO*	*TsaCRC*	-	*TsaFIL*	*TsaYAB3*	*TsaYAB5*
	LF	*BraYAB2a*	*BraINOb*	*BraCRC*	-	-	*BraYAB3*	
Brassica rapa (A genome)	MF1	*BraYAB2b*	-	-	-	*BraFILc*	-	*BraFILb*
	MF2	*BraYAB2c*	*BraINOa*	-	*BraYAB5*	*BraFILa*	-	
	LF	*BniYAB2a*	*BniINOa*	*BniCRCa*	-	*BniFILb*	*BniYAB3*	
Brassica nigra (B genome)	MF1	*BniYAB2b*	-	*BniCRCb*	-	*BniFILa*	-	
	MF2	*BniYAB2c*	*BniINOb*	-	*BniYAB5*	*BniFILc*	-	
	LF	*BolYAB2c*	*BolINOb*	*BolCRC*	-	-	*BolYAB3*	
Brassica oleracea (C genome)	MF1	*BolYAB2b*	-	-	-	*BolFILb*	-	*BolFILa*
	MF2	*BolYAB2a*	*BolINOa*	-	*BolYAB5*	*BolFILc*	-	
	LF	*BjuYAB2a*	*BjuINOa*	*BjuCRCc*	-	-	*BjuYAB3a*	
Brassica juncea (A subgenome)	MF1	*BjuYAB2b*	-	-	-	*BjuFILb*	-	
	MF2	*BjuYAB2e*	*BjuINOb*	-	*BjuYAB5a*	*BjuFILa*	-	*BjuFILc, BjuFILg, BjuYAB2g*
	LF	*BjuYAb2d*	*BjuINOc*	*BjuCRCa*	-	*BjuFILe*	*BjuYAB3b*	
Brassica juncea (B subgenome)	MF1	*BjuYAB2c*	-	*BjuCRCb*	-	*BjuFILf*	-	
	MF2	*BjuYAB2f*	*BjuINOd*	-	*BjuYAB5b*	*BjuFILd*	-	
	LF	*BnaYAB2f*	*BnaINOc*	*BnaCRCb*	-	-	-	
Brassica napus (A subgenome)	MF1	*BnaYAB2b*	-	-	-	-	-	
	MF2	*BnaYAB2e*	*BnaINOa*	-	*BnaYAB5b*	*BnaFILf*	-	*BnaFILa, BnaFILb, BnaFILe, BnaYAB3a, BnaYAB3b*
	LF	*BnaYAB2c*	*BnaINOd*	*BnaCRCa*	-	-	-	
Brassica napus (C subgenome)	MF1	*BnaYAB2d*	-	-	-	*BnaFILc*	-	
	MF2	*BnaYAB2a*	*BnaINOb*	-	*BnaYAB5a*	*BnaFILd*	-	
Total gene number		36	29	23	16	30	18	21
