# Supplementary material for: Evolutionary Analysis of the YABBY Gene Family in Brassicaceae
Source: Plants (Basel). 2021 Dec 8;10(12):2700. doi: 10.3390/plants10122700 (PMC8704796; doi:10.3390/plants10122700)
Supplement: Supplementary file 1 [file plants-10-02700-s001.zip › Figure S1.pdf]

Figure S1 List of 364 YABBY homologous protein sequences (fasta format) identified from the 37 Brassicaceae genomes

```
>Aethionema arabicum FIL
MSMSNSPSSAVFSPDHSLSPSEHLCYVQCKFCETILAVSVPYTSLFKTVTVRCGCCTNLLSVNTRSLLLPASNQLQLQLGPHT
YYNPQNILEELRDAPTNMNMMMNQHPNMNDIPSFMDLRGLDQQHEIPKAPPVNRPPEKRQRVPSAYNRFIKEEIQRIKAGNPD
ISHREAFSAAAKNWAHFPHIHFGLVPENQPVKKTNMPQQEAEENVVMKEGFYAPAANVGVTPY
>Aethionema arabicum YAB2
VSVPYASLFTLVTVRCGHCTNLLSLNIGVSLHQTSAPPVHQDLQLHKQHTSSSTARIDYGSSSRNNSSLSESVDRDTPRMPPI
RPPEKRQRVPSAYNRFIKEEIQRIKACNPEISHREAFSTAAKNWAHFPHIHFGLKLDSNKKGKQLDQTVAGQKANGYY
>Aethionema arabicum YAB3
MSSSSPSSSSSSDQPLYPQHLCYVHCTICDTLLAVNVPSTNLLSVVTVRCGHCTTLLTVDVRLLFQDLGQNLFSHRQNLTVEN
ENENEKPNANYLGEEATRNNLLLASTASTPTLTPHPPQIVGEVPHPLLQPSLRTPSMKRQRTPSAYNRFIKEEIQRIKAENPN
IAHREAFSAAAKNGAEDPVGRETRDAFLRFS
>Aethionema arabicum YAB5
MADQLCYIPCNFCNIVLAVSVPCSSLFDIVTVRCGHCTNLWSVNMAASLQSLSNPNFQATNYTIPEYGSSSRGHNKVSSRISS
RTMTDQRVVNRPPEKRQRVPSAYNQFIKEEIQRIKANNPDISHREAFSTAAKNWAHFPHIHFGLMLESNKQAKLA
>Aethionema arabicum INO
MTRIPNMTTLNHLFDLPGQICHVQCGFCTTILLVSVPFTSLSMMVTVRCGHCSSLLSVNLMKASFIPLHLLASLSNLDHEVGQ
EETVATTDGVEEEEGWKVNNVDKDSPTTLVSSNSDNEDEDKDVSHRVYQVVNKPPEKRQRAPSAYNCFIKEEIRRLKAQNPSM
AHKEAFSLAAKNWAHFPPVHHKRAASDQCFCQEDDNNVALIPHQEEHEESNTNGFRERKAQRHSIWGKSPFE
>Aethionema arabicum CRC
MNLEDKPTMASRPSPQSEHLYYVRCSICNTILAVGIPLKRMLDTVTVKCGHCGNLSFLTTSPPLQGHVSLTLQMQSFGGSHDQ
YKKGTSSSSSSSTSSEQPSSPTAPFVVKPPEKKQRLPSAYNRFMRDEIQRIKTAHPEIPHREAFSAAAKNWAKYIPNSPTSLT
SGGNTINGLSFGENK
>Alyssum linifolium FIL
MSMSSPSSAVFSPDHLSPSEHLCYVQCNFCETILAVSVPYTSLFKTVTVRCGCCTNLLSVNMRTHFLPASNPLQLQLGPHSYF
NSQNILEELRDSPSNMMMMNQHPNMNDIPTFMDLHQQHEIPKAPPVNRPPEKRQRVPSAYNRFIKEEIQRIKAGNPDISHREA
FSAAAKNWAHFPHIHFGLVPDNQPVKKTNMPQQEGEENMVMKEGFYAPAANVGVTPY
>Alyssum linifolium YAB2a
MSVDLSSERVCYVHCSFCTTILAVSVPYASLFTLVTVRCGHCTNLLSLNIGVSLHQTSPPPIHQDLQPHKQHITSSVTRKDCA
SSSSRSTNNLSENIDRETPRMPPIRPPEKRQRVPSAYNRFIKEEIQRIKACNPEISHREAFSTAAKNWAHFPHIHFGLKLDGN
KKGKQLDQSVAGQKSNGYY
>Alyssum linifolium YAB2b
MSVDLSPERVCYVHCSFCTTILAVSVPYASLFTLVTVRCGHCTNLLSLNIGVSLHQTSPPPIHQDLQPHKQHITSSVTRKDCA
SSSSRSTNNLSENIDREAPRMPPIRPPEKRQRVPSAYNRFIKEEIQRIKACNPEISHREAFSTAAKNWAHFPHIHFGLKLDGN
KKGKQLDQSVAGQKSNGYY
>Alyssum linifolium YAB3
MSMSSSSAPAFSPDHISSLDQLCYVHCSFCDTVLAVSVPPSSLYKTVTVRCGHCSNLLSVTVSMRALLLPSVSNHGHSFIPPP
PPSNLLEEMRSGGQNINMNLMMSHHQGAHHPNESLVMATRNGRVDHLQEMPRPPPANRPPEKRQRVPSAYNRFIKEEIQRIKA
GNPDISHREAFSAAAKNWAHFPHIHFGLMADHPPTKKANVRQQEGEDVMMGREGFYGSTANVGVTHN
>Alyssum linifolium YAB5a
MSSHRLSLLQHKQLFFGLIFLRSIMANSATTSEQLCYIPCNFCNIVLAVSVPCSSLFDIVTVRCGHCTNMWSVNMAAALQSLS
RPNFQATNYALPEYGSSSRSHTKIPSRISNRNITEQRVVNRPPEKRQRVPSAYNQFIKEEIKRIKANNPDISHREAFSTAAKN
WAHFPHIHFGLMLESNKQAKLA
```

>Alyssum linifolium YAB5b
MSSHRLSLLQHKQLFFGLIFLRSIMANSATTSEQLCYIPCNFCNIVLAVSVPCSSLFDIVTVRCGHCTNLWSVNMAAALQSLS
RPNFQATNYALPEYGSSSRSHTKIPSRISNRNITEQRVVNRPPEKRQRVPSAYNQFIKEEIKRIKANNPDISHREAFSTAAKN
WAHFPHIHFGLMLESNKQAKLA
>Alyssum linifolium INOa
MTRKPNMTTLNHLFDLPGQICHVQCGFCTTILLVSVPFTSLSMVVTVRCGHCTSLLSVNLMKASFIPLHLLTSLSGHLDEAGK
EEVAAIDGVEEEAWKVNQEKENSPTTLVTSSDNEDEDVSRVYQVVNKPPEKRQRAPSAYNCFIKEEIRRLKAQNPSMAHKEAF
SLAAKNWAHFPPVHNKRGASDLCFCEEDGNAELPCNNALEDHEESNNGFRERKAQRHSIWGKSPFE
>Alyssum linifolium INOb
MTRKPNMTTLNHLFDLPGQICHVQCGFCTTILLVSVPFTSLSMVVTVRCGHCTSLLSVNLMKASFIPLHLLTSLSGHLDEAGK
EEVAAIDGVEEEAWKVNQEKENSPTTLVTSSDNEDEDVSRVYQVVNKPPEKRQRAPSAYNCFIKEEIRRLKAQNPSMAHKEAF
SLAAKNWAHFPPVHNKRGASDLCFCEEDGNAELPCNNALEDHEESNNGFRERKAQRHSIWGKSPFE
>Alyssum linifolium CRCa
MNLEEKPTMTSRASPQAEHLYYVRCSICNTILAVGIPLKRMLDTVTVKCGHCGNLSFLTTSPPLQGHVSLTLQMQSFGGSEYK
KGSSSSSSSSTSSDQPPSPTPPFVVKPPEKKQRLPSAYNRFMRDEIQRIKSANPEIPHREAFSAAAKNWAKYIPNSSTSLTSG
LNNINGLGFGENK
>Alyssum linifolium CRCb
MNLEEKPTMTSRASPQAEHLYYVRCSICNTILAVGIPLKRMLDTVTVKCGHCGNLSFLTTSPPLQGHVSLTLQMQSFGGSEYK
KGSSSSSSSSTSSDQPPSPTPPFVVKPPEKKQRLPSAYNRFMRDEIQRIKSANPEIPHREAFSAAAKNWAKYIPNSPTSLTSG
LNNINGLGFGENK
>Arabidopsis halleri FIL
MSMSSMSSPSSAVCSPDHFSPSEHLCYVQCNFCQTILAVSVPYTSLFKTVTVRCGCCTNLLSVNMRSYVLPASNQLQLQLGPH
SYFNPQDILEELRDAPSNMNMMMMNQHPTMNDIPSFMDLHQQHEIPKAPPVNRPPEKRQRVPSAYNRFIKEEIQRIKAGNPDI
SHREAFSAAAKNWAHFPHIHFGLVPDNQPVKKTNMPQQEGDDNMVMKEGFYAPAAANVGVTPY
>Arabidopsis halleri YAB2
MSVDLSSERVCYVHCSFCTTILAVSVPYASLFTLVTVRCGHCTNLLSLNIGVSLHQTAPPPIHQDLQPPHKQHTTSMVTRKDC
ASSSRSTNNLSEHIDREAPRMPPIRPPEKRQRVPSAYNRFIKEEIQRIKACNPEISHREAFSTAAKNWAHFPHIHFGLKLDGN
KKGKQLDQSVAGQKSNGYY
>Arabidopsis halleri YAB3
MSSMSMSSSSAPAFPPDHFSSSEQLCYVHCSFCDTVLAVSVPPSSLFKTVTVRCGHCSNLLSVTVSMRALLLPSVSNLGHSFL
PPPPPPSPPNLLEEMRSGGQNINMNMMMSHHGAAHHPNESLVMPTRNGRVDHVQEMPRPPANRPPEKRQRVPSAYNRFIKEEI
QRIKAGNPDISHREAFSAAAKNWAHFPHIHFGLMADHPPTKKANVRQQEGEDSMMGREGFYGSTANVGVTHN
>Arabidopsis halleri YAB5
MANSAMATEQLCYIPCNFCNIILAVSVPCSSLFDIVTIRCGHCTNLWSVNMAAALQSLSRPNFHATNNAVPEYGSSSRDHTKI
PSRISTRTITEQRIVNRPSEKRQRVPSAYNQFIKEEIQRIKANNPDISHREAFSTAAKNWAHFPHIHFGLMLESNKQAKLA
>Arabidopsis halleri INO
MTKLPNMTTTLNHLFDLPGQICHVQCGFCTTILLVSVPFTSLSMVVTVRCGHCTSLLSVNLMKASFIPLHLLASLSHLEETGK
EEVPVIDGVEEEAWKVNQEKENSPTTLVSSSDNEDEDVSRVYQVVNKPPEKRQRAPSAYNCFIKEEIRRLKAQNPSMAHKEAF
SLAAKNWAHFPPVHNKRAASDQCFCEEDNNAILPCNALEDHEESNNGFRERKAQRHSIWGKSPFE
>Arabidopsis halleri CRC
MNLEEKPTMAASRASPQTEHLYYVRCSICNTILAVGIPLKRMLDTVTVKCGHCGNLSFLTTTPPLQGHVSLTLQMQSFGGSEY
KKGSSSSSSSSSTSSDQPPSPTPPFVVKPPEKKQRLPSAYNRFMRDEIQRIKSANPEIPHREAFSAAAKNWAKYIPNSPTSITS
GGHNMIHGLGFGEKK
>Arabidopsis lyrata FIL
MSMSSMSSPSSAVCSPDHFSPSEHLCYVQCNFCQTILAVSVPYTSLFKTVTVRCGCCTNLLSVNMRSYVLPASNQLQLQLGPH

SYFNPQDILEELRDAPSNMNMMMMNQHPTMNDIPSFMDLHQQHEIPKAPPVNRPPEKRQRVPSAYNRFIKEEIQRIKAGNPDI
SHREAFSAAAKNWAHFPHIHFGLVPDNQPVKKTNMPQQEGEDNMVMKEGFYAPAAANVGVTPY
>Arabidopsis lyrata YAB2
MSVDLSSERVCYVHCSFCTTILAVSVPYASLFTLVTVRCGHCTNLLSLNIGVSLHQTAPPPIHQDLQPHKQHTTSLVTRKDCA
SSSRSTNNLSEHIDREAPRMLPIRPPEKRQRVPSAYNRFIKEEIQRIKACNPEISHREAFSTAAKNWAHFPHIHFGLKLDGNK
KGKQLDQSVAGQKSNGYY
>Arabidopsis lyrata YAB3
MSSMSMSSSSAPAFPPDHFSSSEQLCYVHCSFCDTVLAVSVPPSSLFKTVTVRCGHCSNLLSVTVSMRALLLPSVSNLGHSFL
PPPPPPSPPNLLEEMRSGGQNINMNMMMSHHGAAHHPDESLVMPTRNGRVDHLQEMPRPPANRPPEKRQRVPSAYNRFIKEEI
QRIKAGNPDISHREAFSAAAKNWAHFPHIHFGLMADHPPTKKANVRQQEGEDSMMGRDREGFYGSTANVGVTHN
>Arabidopsis lyrata YAB5
MANSAMATEQLCYIPCNFCNIILAVSVPCSSLFDIVTVRCGHCTNLWSVNMAAALQSLSRPNFHATNYAVPEYGSSSRDHTKI
PSRISTRTITEQRIVNRPSEKRQRVPSAYNQFIKEEIQRIKANNPDISHREAFSSAAKNWAHFPHIHFGLMLESNKQAKLA
>Arabidopsis lyrata INO
MTTLNHLLDLPGQICHVQCGFCTTILLVSVPFTSLSMVVTVRCGHCTSLLSVNLMKASFIPLHLLASLSHLDETGKEEVAATD
AVEEEAWKVNQEKENSPTTLVSSSDNEDEDVSRVYQVVNKPPEKRQRAPSAYNCFIKEEIRRLKAQNPSMAHKEAFSLAAKNW
AHFPPVHNKRAASDQCFCEEDNNAILPCNAFEDHEESNNGFRERKAQRHSIWGKSPFEYNNLGYEILTKK
>Arabidopsis lyrata CRC
MNLEEKPTMAASRASPQTEHLYYVRCSICNTILAVGIPLKRMLDTVTVKCGHCGNLSFLTTTPPLQGHVSLTLQMQSFGGSEY
KKGSSSSSSSSTSSDQPPSPTPPFVVKPPEKKQRLPSAYNRFMRDEIQRIKSANPEIPHREAFSAAAKNWAKYIPNSPTSITS
GGHNMIHGLGFGEKK
>Arabidopsis thaliana FIL
MSMSSMSSPSSAVCSPDHFSPSDHLCYVQCNFCQTILAVNVPYTSLFKTVTVRCGCCTNLLSVNMRSYVLPASNQLQLQLGPH
SYFNPQDILEELRDAPSNMNMMMMNQHPTMNDIPSFMDLHQQHEIPKAPPVNRPPEKRQRVPSAYNRFIKEEIQRIKAGNPDI
SHREAFSAAAKNWAHFPHIHFGLVPDNQPVKKTNMPQQEGEDNMVMKEGFYAPAAANVGVTPY
>Arabidopsis thaliana YAB2
MSVDFSSERVCYVHCSFCTTILAVSVPYASLFTLVTVRCGHCTNLLSLNIGVSLHQTSAPPIHQDLQPHRQHTTSLVTRKDCA
SSSRSTNNLSENIDREAPRMPPIRPPEKRQRVPSAYNRFIKEEIQRIKACNPEISHREAFSTAAKNWAHFPHIHFGLKLDGNK
KGKQLDQSVAGQKSNGYY
>Arabidopsis thaliana YAB3
MSSMSMSSSSAPAFPPDHFSSTDQLCYVHCSFCDTVLAVSVPPSSLFKTVTVRCGHCSNLLSVTVSMRALLLPSVSNLGHSFL
PPPPPPPPPNLLEEMRSGGQNINMNMMMSHHASAHHPNEHLVMATRNGRSVDHLQEMPRPPPANRPPEKRQRVPSAYNRFIKE
EIQRIKAGNPDISHREAFSAAAKNWAHFPHIHFGLMADHPPTKKANVRQQEGEDGMMGREGFYGSAANVGVAHN
>Arabidopsis thaliana YAB5
MANSVMATEQLCYIPCNFCNIILAVNVPCSSLFDIVTVRCGHCTNLWSVNMAAALQSLSRPNFQATNYAVPEYGSSSRSHTKI
PSRISTRTITEQRIVNRPPEKRQRVPSAYNQFIKEEIQRIKANNPDISHREAFSTAAKNWAHFPHIHFGLMLESNKQAKIA
>Arabidopsis thaliana INO
MTKLPNMTTTLNHLFDLPGQICHVQCGFCTTILLVSVPFTSLSMVVTVRCGHCTSLLSVNLMKASFIPLHLLASLSHLDETGK
EEVAATDGVEEEAWKVNQEKENSPTTLVSSSDNEDEDVSRVYQVVNKPPEKRQRAPSAYNCFIKEEIRRLKAQNPSMAHKEAF
SLAAKNWAHFPPAHNKRAASDQCFCEEDNNAILPCNVFEDHEESNNGFRERKAQRHSIWGKSPFE
>Arabidopsis thaliana CRC
MNLEEKPTMTASRASPQAEHLYYVRCSICNTILAVGIPLKRMLDTVTVKCGHCGNLSFLTTTPPLQGHVSLTLQMQSFGGSDY
KKGSSSSSSSSTSSDQPPSPSPPFVVKPPEKKQRLPSAYNRFMRDEIQRIKSANPEIPHREAFSAAAKNWAKYIPNSPTSITS
GGHNMIHGLGFGEKK
>Boechera stricta FIL

MSMSSPSSAVFSPDHLSPSEHLCYVQCNFCDTILAVSVPYTSLFKTVTVRCGCCTTLLSVNMRLVLPASNQLQLQLGPHSYFD

SQNILEELRDAPSNMNMMMLNQHPNMNDIPSFMDLHQQHEIPKAPPVNRPPEKRQRVPSAYNRFIKEEIQRIKAGNPDISHRE

AFSAAAKNWAHFPHIHFGLVPDNQPVKKTNMPQQEGEDNMVMKDGFYAPAANVGVTPY

>Boechera stricta YAB2

MSVDLSSERVCYVHCSFCTTILAVSVPYASLFTLVTVRCGHCTNLLSLNIGVSLHQTSPPPINQDLQPHKQHITSSVTRKDFG

SSSRSTNNISTTLPENIDREAPRMPPIRPPEKRQRVPSAYNRFIKEEIQRIKACNPEISHREAFSTAAKNWAHFPHIHFGLKL

DGNKKGKQLDQTVASQKSNGYY

>Boechera stricta YAB3

MSSMSMSSSSAPAYPPDHISSSDQLGYVHCSFCDTVLAVSVPPSSLFKTVTVRCGHCSNLLSVTVNMRTLLLPSVSNIGHSFI

PPPPPPPNLLEEMRSGGQSINMNMMMSHHGAAYHSNESLATRNGRVDHLQEIPRPPPANRPPEKRQRVPSAYNRFIKEEIQRI

KAGNPDISHREAFSAAAKNWAHFPHIHFGLMADHPPTKKADVRQQEGEDVMMGREGFYGSAANVGVTHN

>Boechera stricta YAB5

MANSAMATEQLCYIPCNFCNIVLAVSVPCSSLFDIVTVRCGHCTNMWSVNMAAALQSLSRPNFQATNYAVPEYGSSSRGHTKI

PSRITTRAITEQRIVNRPPEKRQRVPSAYNQFIKEEIQRIKASNPDISHREAFSTAAKNWAHFPHIHFGLMLESNKQAKLA

>Boechera stricta INO

MTKIPNMTTTLNHLFDLPGQICHVQCGFCTTILLVSVPFTTLSMVVTVRCGHCTSLLSVNLMKASFVPLHLLASLSHLDEAGK

EEVVATDGVEEEAWKVNSLEKENSPTTLVSSSDNEDEDVSRVYQVVNKPPEKRQRAPSAYNCFIKEEIRRLKAQNPSMAHKEA

FSLAAKNWAHFPPVHNKRAASDHCFCEEDNNAIPPCNALEDHEENNNGFRERKAQRHSIWGKSPFE

>Boechera stricta CRC

MNLEEKPTMTSKASPQAEHLYYVRCSICNTILAVGIPLKRMLDTVTVKCGHCGNLSFLTTSPPLQGHVSLTLQMQSFGGSEYK

KGNSSSSSSSTSSDKPPSPTPPFVVKPPEKKQRLPSAYNRFMRDEIQRIKSANPEIPHREAFSAAAKNWAKYIPNSPTSTTSG

GNNIHGLGFGEKK

>Brassica rapa FILa

MSMSSMSSPSSAVFSPEPLSPSDHLCYVQCNFCETILAVSVPYTSMFKTVTVRCGCCTNLLSVNMRSAALPASNQLQLGPHSY

FNTQNILEELRDAPSNMNMMMMNQHPNMNDIPSFMNIHQHEITKAPPVNRPPEKRQRVPSAYNRFIKEEIQRIKAGNPDISHR

EAFSAAAKNWAHFPHIHFGLAPDNQPVKKTNMPQQDGENNMGMKEGLYAPAAHVGVAPY

>Brassica rapa FILb

MSMSSMSSPSSAVFSPEHLSPSEHLCYVQCNFCETILAVSVPYTSLFKTVTVRCGCCTNLLSVNMRSLVLPASNQLQLQLGPQ

SYFTPQNILEELREAPSNMNMMMINQHPNMNDIPSLMDLHQKHEIPKAPPTNRPPEKRQRVPSAYNRFIKEEIQRIKAGNPDI

SHREAFSAAAKNWAHFPHIHFGLMPDNQ PVKKTNMPQQEGEENMGMKEGFYAPY

>Brassica rapa FILc

MSMSSMSSPSSAVFSPENLSPDPLSPSEQLCYVQCNYCETILAVSVPYTSMFKTVTVRCGCCTNLISVNMRSLVLPASNQLQL

QLGPHSYFTPQNILEELKDAPSNMNMMMMNQHPNMNDIPSFMDLHQQHEIPKAPPVNRPPEKRQRVPSAYNRFIKEEIQRIKA

GNPDISHREAFSAAAKNWAHFPHIHFGLAPDNQPVKKTNMPQQEGEDNMGMKEGFYAPAANVGVIPY

>Brassica rapa YAB2a

MSIDISSERVCYVHCNFCTTILAVSVPYASLFTLVTVRCGHCTNLLSLNIGVSLHQSSPAPPIHQDLQHKQHITSSVTRKEHG

SSSRSFNHFSTTLSENVEREAPRMPPIRPPEKRQRVPSAYNRFIKEEIQRIKAGNPEISHREAFSTAAKNWAHFPHIHFGLKL

DGNNKKGKQIDQSVAGQKSNGYY

>Brassica rapa YAB2b

MSIDLSSDRVCYVHCNFCTTILAVSVPYASMFTLVTVRCGHCTNLLSLNIGVSLHQSPPTPIHQDLQHKQQITTSITRKEYGS

SSRSSNHFSTTLSENVDREAPRMPPIRPPEKRQRVPSAYNRFIKEEIQRIKAGNPEISHREAFSTAAKNWAHFPHIHFGLKLD

GNKKGKQLDQTVAGQKSNGYY

>Brassica rapa YAB2c

MSIDLSSDRVCYVLCNFCTTTLAVSVPYASLFTLVTVRCGHCTNLLSLNIGVSLHQSSPPPIHQDLQPKQHIASSVTRKEWGS

SSRSSNHFSTTLSENVDREAPRMPPIRPPEKRQRVPSAYNRFIKEEIQRIKAGNPAISHREAFSTAAKNWAHFPHIHFGLKLD

GNKKGKQLDQTVAGHKSNGYF

>Brassica rapa YAB3

MSSMSMSSSSAPAYPPDHISSSDQLCYVHCSFCDTVLAVSVPPSSLFKTVTVRCGHCSNLLSVTVNMRALLLPSVSNIGHSFL

PSPPPPPPPNLLEEMRNGGQNINMNMMMSHHAAAHHSNESFVMATRVRSVDLQEMPRPPPANRPPEKRQRVPSAYNRFIKEEI

QRIKAGNPDISHREAFSAAAKNWAHFPHIHFGLMPDHPPTKKANVRQQEGEEVMMGREGFYGSAANVGVTHN

>Brassica rapa YAB5

MANSATAAEQLCYIPCNFCNIVLAVSVPCSSLFDIVTVRCGHCTNLWSVNMAAALQSLSRPNFQVTPYAMPEYGSSSRGNTKI

SSRISARTISEQRIVNRPPEKRQRVPSAYNQFIKEEIQRIKANNPDISHREAFSTAAKNWAHFPHIHFGLMLESNKQAKLA

>Brassica rapa INOa

MTKMANMTTLNQLFDLPGQICHVQCGFCTTILLVSVPFTSLSMVVTVRCGHCTSLLSVNLMKASFIPLHLLTSLSHLDETEKE

EVAATTDGVEKEAWKVTQEKENSPTTLVTSSDNEDEDEDKDVSRVYQVVNKPPEKRQRAPSAYNCFIKEEIRRLKAQNPSMAH

KEAFSLAAKNWAHFPPVQNKRTASDQCFCEEDNNVLLSCNALEDHEVSNNGFRERKAQRHSIWGKSPFE

>Brassica rapa INOb

MTTLNQLFDLPGQVCHVQCGFCTTILLVSVPFTSLSMVVTVRCGHCTSLLSVNLMKASFIPLHLLTSLSHMDEKGHEEVAATT

DGVEEEAWKVNQEKENSPATLVTSSESEDEDRDVSRVYQVVNKPPEKRQRAPSAYNCFIKEEIRRLKAQNPSMAHKEAFSLAA

KNWANFPPVQNKRAASDQYFCEDDNNALLSCNALGDHDESNNGFRERKAQRHSIWGKSPFD

>Brassica rapa CRC

MNLEEKPTMASRVSPQAEHLYYVRCSICNTILAVGIPMKRMLDTVTVKCGHCGNLSFLTTTPPLQGHVSLTLQMQSFGGSEYK

KGSSSSSSSSTSSDQPPSPRPPFVVKPPEKKQRLPSAYNRFMRDEIQRIKSANPEIPHREAFSAAAKNWAKYIPNSPTSITSG

ASNIHGFGFGEKK

>Brassica nigra FILa

MSMSSMSSPSSAVFSPENLSPDPLSPSEQLCYVQCNYCETILAVSVPYTSMFKTVTVRCGCCTNLISVNMRSLVLPASNQLQL

QLGPHSYFTPQNILEELKDAPSNMNMMMMNQHPNMNDIPSFMDLHQQHEIPKAPPVNRPPEKRQRVPSAYNRFIKEEIQRIKA

GNPDISHREAFSAAAKNWAHFPHIHFGLAPDNQPVKKTNMPQQESEDNMGMREGFYPPAANVGVIPY

>Brassica nigra FILb

MSMSSMSSPSSAVFSQEHLSPSEHLCYVQCNFCETILAVSVPYTSLFKTVTVRCGCCTNLLSVNMRSLVLPASNQLQLQLGPQ

SYFAPQNILEELREPPSNMNMMMINQHPNVNDITSFMDLQQHHEIPKAPTANRPPEKRQRVPSAYNRFIKEEIQRIKAGNPDI

SHREAFSAAAKNWAHFPHIHFGLMPDNQPVKKTNMPQQEGEENMGMKEGFYARAANVGMTPY

>Brassica nigra FILc

MSMSSMSSPSSAVFSPEHLSPSDHLCYVQCNFCETILAVSVPYTSMFKTVTVRCGCCTNLLSVNMRSAALPASNQLQLQLGPH

SYFNTQNILEELRDAPSNMNMMMMNQHPSMNDIPSFMNIHQQHEIPKAPPVNRPPEKRQRVPSAYNRFIKEEIQRIKAGNPDI

SHREAFSAAAKNWAHFPHIHFGLAPDNQPVKKTNMPQQEGEDNMGMKDGFYAPAANVGVAPY

>Brassica nigra YAB2a

MSIDLSSERVCYVHCNFCTTILAVSVPYASLFTVVTVRCGHCTNLLSLNIGVSLHQSSPPPIHQDLQHKQHITSSVTRKEHGS

SSRSFNHFSTTLSENIEREAPRMPPIRPPEKRQRVPSAYNRFIKEEIQRIKASNPQISHREAFSTAAKNWAHFPHIHFGLKLD

GNKKGKQIDQTVAGQNSNGYY

>Brassica nigra YAB2b

MSIDLSSDRVCYVHCNFCTTILAVSVPYASLFTLVTVRCGHCTNLLSLNIGVSLHQTSPTHPIHQDLQHKQHITSSVTRKEYG

SSSRSSNHFSTTLTENVDREAPKMPPIRPPEKRQRVPSAYNRFIKEEIQRIKAGNPEISHREAFSTAAKNWAHFPHIHFGLKL

DGNKKGKQLDQTVAGQKSNGYY

>Brassica nigra YAB2c

MSIDLSSDRVCYVHCNFCTTILA

VSVPYASLFTLVTVRCGHCTNLLSLNMGVPLHQSSPPPIHQDL

QPKQHITSSVTRKEWGSSSRSSNHFSTTLSENVDREAPRMPPIRPPEKRQRVPSAYNRFIKEEIQRIKAGNPAISHREAFSTA

AKNWAHFPHIHFGLKLDGNKKGKQLDQTVAGHKSYGYF

>Brassica nigra YAB3
MSSMSMSSSSAPAYPPDHISSSDQLCYVHCSFCDTVLAVSVPPSSLFKTVTVRCGHCSNLLSVTVNMRALLLPSVSNIGHSFL
PSPPPNLLEEMRNGGQNINTNMMMSHQAAAHHSNESFVMATRNGRVDLQEMPRPPPANRPPEKRQRVPSAYNRFIKEEIQRIK
AGNPDISHREAFSAAAKNWAHFPHIHFGLMPDHPPTKKANVRQQEGEEVMMGREGFYGSAANVGVTHN
>Brassica nigra YAB5
MANSATAAEQLCYIPCNFCNIVLAVSVPCSSLFDIVTVRCGHCTNLWSVNMAAALQSLSRPNFQATPYATPEYGSSSRGHTKI
SSRISARAISEQRVVNRPEKRQRVPSAYNQFIKEEIQRIKANNPDISHREAFSTAAKNWAHFPHIHFGLMLESNKQAKLA
>Brassica nigra INOa
MTTLNQLFDLPGQICHVQCGFCATILLVSVPLTSLSMVVTVRCGHCTSLLSVNLLKASFIPLHLLTSLSHLDEAGKQEAAATT
DGVEEEAWKVNQEETSPTTLVTSSDNEDEDRDLSRVYQVVNKPPEKRQRAPSAYNCFIKEEIKRLKVQNPSMAHKEAFSLAAK
NWANFPPVQNKRAASDQCFYEDDNNALLSCNALEDHEERNNGFRERKAQRHSIWGKSPFE
>Brassica nigra INOb
MPNMSTLNQLFDLPGQICHVQCGFCTTILLVSVPFASLSMVVTVRCGHCTSLLSVNLMKASFLPLHLLTSLCQLDETGKDEVA
ATTDDVEEETLKVNQEKGNSPTTLVTSSDNEDEDQDVSRVYQVVNKPPEKRQRAPSAYNCFIKEEIRRLKAQNPSMAHKEAFS
LAAKNWAHFPPVQNKRAASDQCFCEEDDNVLLSCNALEDHELSNNGFRERKAQRHSIWGKSPFE
>Brassica nigra CRCa
MNLEEKPTMASRASPQAEHLYYVRCSICNTILAVGIPMKRMLDTVTVKCGHCGNLSFLTTSPPLQGHVSLTLQMQSFGGSEYK
KGSSSSSSSSTSSDQPPSPRPPFVVKPPEKKQRLPSAYNRFMRDEIQRIKSANPEIPHREAFSAAAKNWAKYIPNSPTSITSG
GSNINGFGFGEKK
>Brassica nigra CRCb
MASRTSPQSEHIYYVRCSICNTILAVGIPSKRMLDTVTVKCGHCGSLSFLTTSHPLKGHVSLSLQMPSSSSGGGGYKKGSSSS
SSSSTSSDQPSSPRPPFVVKPPGKKQRLPSAYNRFMRDEIQRIKSANPEIPHREAFSTAAKNWAKYIPNSPTSITSGAKNING
FGFPEKK
>Brassica oleracea FILa
MSMSSMSSPSSAVFSAEHLSPSEHLCYVQCNFCETILAVSVPYTSLFKTVTVRCGCCANLLSVNMRSLVLPASNQIQLQLGPQ
SYFTPQNILEELREAPSNMNMMMINQHPNMNDIPSFMDLHQKHEIPKAPPTNRPPEKRQRVPSAYNRFIKEEIQRIKAGNPDI
SHREAFSAAAKNWAHFPHIHFGLMPDNQPVKKTNMPQQAGEENMGMKEGFYAPAANVGMTPY
>Brassica oleracea FILb
MSMSSMSSPSSAVFSPENLSPDPLSPSEQLCYVQCNYCETILAVSVPYTSMFKTVTVRCGCCTNLISVNMRSLVLPASNQLQL
QLGPHSYFTPQNILEELKDAPSNMNMMMMNQHPNMNDIPSFMDLHQQHEIPKAPPVNRPPEKRQRVPSAYNRFIKEEIQRIKA
GNPDISHREAFSAAAKNWAHFPHIHFGLAPDNQPVKKTSMPQQEGEDNMGMKEGFYAPAANVGVIPY
>Brassica oleracea FILc
MSMSSMSSPSSAVFSPEPLSPSDHLCYVQCNFCETILAVSVPYTSMFKTVTVRCGCCTNLLSVNMRSAALPASNQLQLGPHSY
FNTQNILEELRDAPSNMNMMMMNQHPNMNDIPSFMNIHRQHEITKAPPVNRPPEKRQRVPSAYNRFIKEEIQRIKAGNPDISH
REAFSAAAKNWAHFPHIHFGLAPDNQPVKKTNMPQQDGEDNMGMKEGLYAPAAHVGVAPY
>Brassica oleracea YAB2a
MSIDLSSDRVCYVLCNFCTTTLAVSVPYASLFTLVTVRCGHCTNLLSLNIGVSLHQSSPPPIHQDLQPKQHITSSVTRKEWGS
SSRSSNNFSTTLSENVDQEAPRMPPIRPPEKRQRVPSAYNRFIKEEIQRIKAGNPAISHREAFSTAAKNWAHFPHIHFGLKLD
GNKKGKQLDQTVAGHKSNGYF
>Brassica oleracea YAB2b
MSIDLSSDRVCYVHCNFCTTILAVSVPYASLFTLVTVRCGHCTNLLSLNIGVSLQQSPPTPPIHQDLQHKQQITTSITRKEYG
SSSRSSNHFSATLSENVDREAPRMPPIRPPEKRQRLPSAYNRFIKEEIQRIKAGNPEISHREAFSTAAKNWAHFPHIHFGLKL
DGNKKGKQLDQSVAGQKSNGYY
>Brassica oleracea YAB2c
MSIDISSERVCYVHCNFCTTILAVSVPYASLFTLVTVRCGHCTNLLSLNIGVSLHQSSPPPIHQDLQQHKQHITSSVTRKEHG

SSSRSFNHFSTTLSENVEREVPRMPPIRPPEKRQRVPSAYNRFIKEEIQRIKAGNPEISHREAFSTAAKNWAHFPHIHFGLKL
DGNKKGKQIDQTVAGQKSNGYY
>Brassica oleracea YAB3
MSSMSMSSSSAPAYPPDHISSSDQLCYVHCSFCDTVLAVSVPPSSLFKTVTVRCGHCSNLLSVTVNMRALLLPSVSNIGHPFL
PSPPPPPPPSLLEEMRNGGQNINMNMMMSHHAAAHHSNESFVMATRNGSVDLQEMPRPPPANRPPEKRQRVPSAYNRFIKEEI
QRIKAGNPDISHREAFSAAAKNWAHFPHIHFGLMPDHPPTKKANEGEEAMMGREGFYGSAANVGVTHN
>Brassica oleracea YAB5
MANSPTAAEQLCYIPCNFCNIVLAVSVPCSSLFDIVTVRCGHCTNLWSVNMAAALQSLSRPNFQVTPYATPEYGSSSRGHTKI
SSRISARTISEQRIVNRPPEKRQRVPSAYNQFIKEEIQRIKANNPDISHREAFSTAAKNWAHFPHIHFGLMLESNKQAKLA
>Brassica oleracea INOa
MANMTTLNQLFDLPGQICHVQCGFCTTILLVSVPFTSLSMVVTVRCGHCTSLLSVNLMKASFIPLHLLTSLSHLDETEKDEVA
ATTDGVEEEAWKVTLEKENSPTTLVTSSDNEDEDEDKDVSRVYQVVNKPPEKRQRAPSAYNCFIKEEIRRLKAQNPSMAHKEA
FSLAAKNWAHFPPVQNKRTASDQCFCEEDNNVLLSCNALEDHEVSNNGFRERKAQRHSIWGKSPFE
>Brassica oleracea INOb
MTKIPNMTTLNQLFDLPGQVCHVQCGFCTTILLVSVPFTSLSMVVTVRCGHCTSLLSVNLMKASFIPLHLLTSLSHMDEKGNE
EVAATTDGGVEEEAWKVNQEKENSPATLVTSSDSEDEDRDVSRVYQVVNKPPEKRQRAPSAYNCFIKEEIRRLKAQNPSMAHK
EAFSLAAKNWANFPPVQNRRAASDQCFCEDDNNALLSCNALGDHDESNNGFRERKAQRHSIWGKSPFD
>Brassica oleracea CRC
MNLEEKPTMASRALPQAENLYYVRCSICNTILAVGIPMKRMLDTVTVKCGHCGNLSFLTTTPPLQGHVSLTLQMQSFDGSEYK
KGSSSSSSSSTSSDQPPSPRPPFVVKPPEKKQRLPSAYNRFMRDEIQRIKSANPEIPHREAFSAAAKNWAKYIPNSPTSIASG
ASYIHGFGFGEKK
>Brassica juncea FILa
MSMSSMSSPSSTVFSPEHLSPSDHLCYVQCNFCETILAVSVPYTSMFKTVTVRCGCCTNLLSVNMRSAALPAPNQLQLQLGPH
SYFNTQNILEELRDAPSNMNMMMMNQHPSMNDIPSFMNIHQQHEIPKAPPVNRPPEKRQRVPSAYNRFIKEEIQRIKAGNPDI
SHREAFSAAAKNWAHFPHIHFGLAPDNQPVKKTNMPQQDGENNMGMKEGLYAPAAHVGVAPY
>Brassica juncea FILb
MSMSSMSSPSSAVFSPENLSPDPLSPSEQLCYVQCNYCETILAVSVPYTSMFKTVTVRCGCCTNLISVNMRSLVLPASNQLQL
QLGPHSYFTPQNILEELKDAPSNMNMMMMNQHPNMNDIPSFMDLHQQHEIPKAPPVNRPPEKRQRVPSAYNRFIKEEIQRIKA
GNPDISHREAFSAAAKNWAHFPHIHFGLAPDNQPVKKTNMPQQEGEDNMGMKEGFYAPAANVGVIPY
>Brassica juncea FILc
MSMSSMSSPSSAVFSQEHLSPSEHLCYVQCNFCETILAVSVPYTSLFKTVTVRCGCCTNLLSVNMRSLVLPASNQLQLGPQSY
FTPQNILEELREAPSNMNMMMINQHPNMNDIPSLMDLHQKHEIPKAPPTNRPPEKRQRVPSAYNRFIKEEIQRIKAGNPDISH
REAFSAAAKNWAHFPHIHFGLMPDNQSVKKTNMPQQEGEENMGMKEGFYAPY
>Brassica juncea FILd
MSMSSMSSPSSTVFSPEHLSPSDHLCYVQCNFCETILAVSVPYTSMFKTVTVRCGCCTNLLSVNMRSAALPAPNQLQLQLGPH
SYFNTQNILEELRDAPSNMNMMMMNQHPSMNDIPSFMNIHQQHEIPKAPPVNRPPEKRQRVPSAYNRFIKEEIQRIKAGNPDI
SHREAFSAAAKNWAHFPHIHFGLAPDNQPVKKTNMPQQEGEDNMGMKDGFYAPAANVGVAPY
>Brassica juncea FILe
MSMSSMSSPSSAVFSQEHLSPSEHLCYVQCNFCETILAVSVPYTSLFKTVTVRCGCCTNLLSVNMRSLVLPASNQLQLQLGPQ
SYFAPQNILEELREPPSNMNMMMINQHPNVNDITSFMDLQQHHEIPKAPTANRPPEKRQRVPSAYNRFIKEEIQRIKAGNPDI
SHREAFSAAAKNWAHFPHIHFGLMPDNQPVKKTNMPQQEGEENMGMKEGFYAPAANVGMTPY
>Brassica juncea FILf
MSMSSMSSPSSAVFSPENLSPDPLSPSEQLCYVQCNYCETILAVSVPYTSMFKTVTVRCGCCTNLISVNMRSLVLPASNQLQL
QLGPHSYFTPQNILEELKDAPSNMNMMMMNQHPNMNDIPSFMDLHQQHEIPKAPPVNRPPEKRQRVPSAYNRFIKEEIQRIKA
GNPDISHREAFSAAAKNWAHFPHIHFGLAPDNQPVKKTNMPQQESEDNMGMREGFYPPAANVGVIPY

>Brassica juncea FILg

MSMSSMSSPSSAVFSPEPLSPSDHLCYVQCNFCETILAVSVPYTSMFKTVTVRCGCCTNLLSVNMRSAALPASNQLQLGPHSY
FNTQNILEELRDAPSNMNMMMMNQHPNMNDIPSFMNIHQHEITKAPPVNRPPEKRQRVPSAYNRFIKEEIQRIKAGNPDISHR
EAFSAAAKNWAHFPHIHFGLAPDNQPVKKTNMPQQDGENNMGMKEGLYAPAAHVGVAPY

>Brassica juncea YAB2a

MSIDISSERVCYVHCNFCTTILAVSVPYASLFTLVTVRCGHCTNLLSLNIGVSLHQSSPAPPIHQDLQHKQHITSSVTRKEHG
SSSRSFNHFSTTLSENVEREAPRMPPIRPPEKRQRVPSAYNRFIKEEIQRIKAGNPEISHREAFSTAAKNWAHFPHIHFGLKL
DGNNKKGKQIDQSVAGQKSNGYY

>Brassica juncea YAB2b

MSIDLSSDRVCYVHCNFCTTILAVSVPYASMFTLVTVRCGHCTNLLSLNIGVSLHQSPPTPIHQDLQQHKQQITTSITRKEYG
SSSRSSNHFSTTLSENVDREAPRMPPIRPPEKRQRVPSAYNRFIKEEIQRIKAGNPEISHREAFSTAAKNWAHFPHIHFGLKL
DGNKKGKQLDQTVAGQKSNGYY

>Brassica juncea YAB2c

MSIDLSSDRVCYVHCNFCTTILAVSVPYASLFTLVTVRCGHCTNLLSLNIGVSLHQTSPTHPIHQDPQHKQHITSSVTRKEYG
SSSRSSNHFSTTLTENVDREAPRMPPIRPPEKRQRVPSAYNRFIKEEIQRIKAGNPEISHREAFSTAAKNWAHFPHIHFGLKL
DGNKKGKQLDQTVAGQKSNGYY

>Brassica juncea YAB2d

MSIDLSSERVCYVHCNFCTTILAVSVPYASLFTIVTVRCGHCTNLLSLNIGVSLHQSSPPPIHQDLQHKQHITSSVTRKEHGS
SSRSFNHFSTTLSENIEREAPRMPPIRPPEKRQRVPSAYNRFIKEEIQRIKASNPQISHREAFSTAAKNWAHFPHIHFGLKLD
GNKKGKQIDQTVAGQNSNGYY

>Brassica juncea YAB2e

MSIDLSSDRVCYVLCNFCTTTLAVSVPYASLFTLVTVRCGHCTNLLSLNIGVSLHQSSPPPIHQDLQPKQHIASSVTRKEWGS
SSRSSNHFSTTLSENVDREAPRMPPIRPPEKRQRVPSAYNRFIKEEIQRIKAGNPAISHREAFSTAAKNWAHFPHIHFGLKLD
GNKKGKQLDQTVAGHKSNGYF

>Brassica juncea YAB2f

MSIDLSSDRVCYVHCNFCTTILAVSVPYASLFTLVTVRCGHCTNLLSLNMGVPLHQSSPPPIHQDLQPKQHITSSVTRKEWGS
SSRSSNHFSTTLSENVDREAPRMPPIRPPEKRQRVPSAYNRFIKEEIQRIKAGNPAISHREAFSTAAKNWAHFPHIHFGLKLD
GNKKGKQLDQTVAGHKSYGYF

>Brassica juncea YAB2g

MSIDLSSDRVCYVHCNFCTTILAVSVPYASMFTLVTVRCGHCTNLLSLNIGVSLHQSPPTPIHQDLQHKQQITTSITRKEYGS
SSRSSNHFSTTLSENVDREAPRMPPIRPPEKRQRVPSAYNRFIKEEIQRIKAGNPEISHREAFSTAAKNWAHFPHIHFGLKLD
GNKKGKQLDQTVAGQKSNGYY

>Brassica juncea YAB3a

MSSMSMSSSSAPAYPPDHISSSDQLCYVHCSFCDTVLAVSVPPSSLFKTVTVRCGHCSNLLSVTVNMRALLLPSVSNIGHSFL
PSPPHLPPPNLLEEMRNGGQNINTNMMMSHQAAAHHSNESFVMATRNGRVDLQEMPRPPPANRPPEKRQRVPSAYNRFIKEEI
QRIKAGNPDISHREAFSAAAKNWAHFPHIHFGLMPDHPPTKKANVRQQEGEEVMMGREGFYGSAANVGVTHN

>Brassica juncea YAB3b

MSSMSMSSSSAPAYPPDHISSSDQLCYVHCSFCDTVLAVSVPPSSLFKTVTVRCGHCSNLLSVTVNMRALLLPSVSNIGHSFL
PSPPHLPPPNLLEEMRNGGQNINTNMMMSHQAAAHHSNESFVMATRNGRVDLQEMPRPPPANRPPEKRQRVPSAYNRFIKEEI
QRIKAGNPDISHREAFSAAAKNWAHFPHIHFGLMPDRPPTKKANVRQQEGEEVMMGREGFYGSAANVGVTHN

>Brassica juncea YAB5a

MANSATAAEQLCYIPCNFCNIVLAVSVPCSSLFDIVTVRCGHCTNLWSVNMAAALQSLSRPNFQATPYATPEYGSSSRGHTKI
SSRISARAISEQRVVNRPPEKRQRVPSAYNQFIKEEIQRIKANNPDISHREAFSTAAKNWAHFPHIHFGLMLESNKQAKLA

>Brassica juncea YAB5b

MANSATAAEQLCYIPCNFCNIVLAVSVPCSSLFDIVTVRCGHCTNLWSVNMAAALQSLSRPNFQATPYATPEYGSSSRGHTKI

SSRISARAISEQRVVNRPEKRQRVPSAYNQFIKREEIQRIKANNPDISHREAFSTAAKNWAHFPHIHFGLILESNKQAKLA

>Brassica juncea INOa

MTKIPNMTTLNQLFDLPGQVCHVQCGFCTTILLVSVPFTSLSMVVTVRCGHCTSLLSVNLMKASFIPLHLLTSLSHMDEKGNE

EVAATTDGVEEEAWKVNQEKENSPATLVTSSDTPEKRQRAPSAYNCFIKEEIRRLKAQNPSMAHKEAFSLAAKNWANFPPVQN

KRAASDQYFCEDDNNALLSCNALGDHDESNNGFRERKAQRHSIWGKSPFD

>Brassica juncea INOb

MTKMANMTTLNQLFDLPGQICHVQCGFCTTILLVSVPFTSLSMVVTVRCGHCTSLLSVNLMKASFIPLHLLTSLSHLDETEKE

EVAATTDGVEKEAWKVTQEKENSPTTLVTSSDNEDEDEDKDVSRVYQVVNKPPEKRQRAPSAYNCFIKEEIRRLKAQNPSMAH

KEAFSLAAKNWAHFPPVQNKRTASDQCFCEEDNNVLLSCNGLEDHEVSNNGFRERKAQRHSIWGKSPFE

>Brassica juncea INOc

MTKIPNMTTLNQLFDLPGQICHVQCGFCATILLVSVPLTSLSMVVTVRCGHCTSLLSVNLLKASFIPLHLLTSLSHLDEAGKQ

EAAATTDGVEEEAWKVNQEETSPTTLVTSSDNEDEDRDVSRVYQVVNKPPEKRQRAPSAYNCFIKEEIKRLKVQNPSMAHKEA

FSLAAKNWANFPPLQNKRAASDQCFYEDDNNALLSCNALEDHEERNNGFRERKAQRHSIWGKSPFE

>Brassica juncea INOd

MPNMSTLNQLFDLPGQICHVQCGFCTTILLVSVPFASLSMVVTVRCGHCTSLLSVNLMKASFLPLHLLTSLCQLDETGKDEVA

ATTDDVEEETLKVNQEKGNSPTTLVTSSDNEDEDQDVSRVYQVVNKPPEKRQRAPSAYNCFIKEEIRRLKAQNPSMAHKEAFS

LAAKNWAHFPPVQNKRAASDQCFCEEDDNVLLSCNALEDHELSNNGFRERKAQRHSIWGKSPFE

>Brassica juncea CRCa

MNLEEKPTMASRVSPQAEHLYYVRCSICNTILAVGIPMKRMLDTVTVKCGHCGNLSFLTTSPPLQGHVSLTLQMQSFGGSEYK

KGSSSSSSSSTSSDQPPSPRPPFVVKPPEKKQRLPSAYNRFMRDEIQRIKSANPEIPHREAFSAAAKNWAKYIPNSPTSITSG

GSNINK

>Brassica juncea CRCb

MASRTSPQSEHIYYVRCSICNTILAVGIPSKRMLDTVTVKCGHCGSLSFLTTSHPLKGHVSLSLQEMPSSSSGGGGYKKGSSS

SSSSSTSSDQPSSPRPPFVVKPPGKKQRLPSAYNRFMRDEIQRIKSANPEIPHREAFSAAAKNWAKYIPNSPTSITSGAKNIN

GFGFPEKK

>Brassica juncea CRCc

MLDTVTVKCGHCGTSRFSPQAPPLQGHVSLTLQMQSFGGSEYKKGSSSSSSSSTSSDQPPSPRPPFVVKPPEKKQRLPSAYNR

FMRDEIQRIKSANPEIPHREAFSAAAKNWAKYIPNSPTSITSGASNIHGFGFGEKK

>Brassica napus FILa

MSMSSMSSPSSAVFSPEHLSPSEHLCYVQCNFCETILAVSVPYTSLFKTVTVRCGCCTNLLSVNMRSLVLPASNQLQLGPQSY

FTPQNILEELREAPSNMNMMMINQHPNMNDIPSFMDLHQKHEIPKAPPTNRPPEKRQRVPSAYNRFIKEEIQRIKAGNPDISH

REAFSAAAKNWAHFPHIHFGLMPDNQSVKKTNMPQQEGEENMGMKEGFYAPY

>Brassica napus FILb

MSMSSMSSPSSAVFSPENLSPDPLSPSEQLCYVQCNYCETILAVSVPYTSMFKTVTVRCGCCTNLISVNMRSLVLPASNQLQL

QLGPHSYFTPQNILEELKDAPSNMNMMMMNQHPNMNDIPSFMDLHQQHEIPKAPPVNRPPEKRQRVPSAYNRFIKEEIQRIKA

GNPDISHREAFSAAAKNWAHFPHIHFGLAPDNQPVKKTSMPQQEGEDNMGMKEGFYAPAANVGVIPY

>Brassica napus FILc

MSMSSMSSPSSAVFSPENLSPDPLSPSEQLCYVQCNYCETILAVSVPYTSMFKTVTVRCGCCTNLISVNMRSLVLPASNQLQL

QLGPHSYFTPQNILEELKDAPSNMNMMMMNQHPNMNDIPSFMDLHQQHEIPKAPPVNRSPEKRQRVPSAYNRFIKEEIQRIKA

GNPDISHREAFSAAAKNWAHFPHIHFGLAPDNQPVKKTSMPQQEGEDNMGMKEGFYAPAANVGVIPY

>Brassica napus FILd

MSMSSMSSPSSAVFSPEPLSPSDHLCYVQCNFCETILAVSVPYTSMFKTVTVRCGCCTNLLSVNMRSAALPASNQLQLGPHSY

FNTQNILEELRDAPSNMNMMMMNQHPNMNDIPSFMNIHRQHEITKAPPVNRPPEKRQRVPSAYNRFIKEEIQRIKAGNPDISH

REAFSAAAKNWAHFPHIHFGLAPDNQPVKKTNMPQQDGEDNMGMKEGLYAPAAHVGVAPY

>Brassica napus FILe

MSMSSMSSPSSAVFSAEHLSPSEHLCYVQCNFCETILAVSVPYTSLFKTVTVRCGCCANLLSVNMRSLVLPASNQIQLQLGPQ
SYFTPQNILEELREAPSNMNMMMINQHPNMNDIPSFMDLHQKHEIPKAPPINRAPEKRQRVPSAYNRFIKEEIQRIKAGNPDI
SHREAFSAAAKNWAHFPHIHFGLMPDNQPVKKTNMPQQAGEENMGMKEGFYAPAANVGMTPY
>Brassica napus FILf
MSMSSMSSPSSAVFSPEPLSPSDHLCYVQCNFCETILAVSVPYTSMFKTVTVRCGCCTNLLSVNMRSAALPASNQLQLGPHSY
FNTQNILEELRDAPSNMNMMMMNQHPNMNDIPSFMNIHQHEITKAPPVNRPPEKRQRVPSAYNRFIKEEIQRIKAGNPDISHR
EAFSAAAKNWAHFPHIHFGLAPDNQPVKKTNMPQQDGENNMGMKEGLYAPAAHVGVAPY
>Brassica napus YAB2a
MSIDLSSDRVCYVLCNFCTTTLAVSVPYASLFTLVTVRCGHCTNLLSLNIGVSLHQSSPPPIHQDLQQPKQHITSSVTRKEWG
SSSRSSNHFSTTLSENVDQEAPRMPPIRPPEKRQRVPSAYNRFIKEEIQRIKAGNPAISHREAFSTAAKNWAHFPHIHFGLKL
DGNKKGKQLDQTVAGHKSNGYF
>Brassica napus YAB2b
MSIDLSSDRVCYVHCNFCTTILAVSVPYASMFTLVTVRCGHCTNLLSLNIGVSLHQSPPTPIHQDLQQHKQQITTSITRKEYG
SSSRSSNHFSTTLSENVDREAPRMPPIRPPEKRQRVPSAYNRFIKEEIQRIKAGNPEISHREAFSTAAKNWAHFPHIHFGLKL
DGNKKGKQLDQTVAGQKSNGYY
>Brassica napus YAB2c
MSIDTSSERVCYVHCNFCTTILAVSVPYASLFTLVTVRCGHCTNLLSLNIGVSLHQSSPPPIHQDLQQHKQHITSSVTRKEHG
SSSRSFNHFSTTLSENVEREVPRMPPIRPPEKRQRVPSAYNRFIKEEIQRIKAGNPEISHREAFSTAAKNWAHFPHIHFGLKL
DGNKKGKQIDQTVAGQKSNGYY
>Brassica napus YAB2d
MSIDLSSDRLCYVHCNFCTTILAVSVPYASLFTLVTVRCGHCTNLLSLNIGVSLQQSPPTPPIHQDLQQHKQQITTSITRKEY
GSSSRSSNHFSATLSENVDREAPRMPPIRPPEKRQRLPSAYNRFIKEEIQRIKAGNPEISHREAFSTAAKNWAHFPHIHFGLK
LDGNKKGKQLDQSVAGQKSNGYY
>Brassica napus YAB2e
MSIDLSSDRVCYVLCNFCTTTLAVSVPYASLFTLVTVRCGHCTNLLSLNIGVSLHQSSPPPIHQDLQQPKQHIASSVTRKEWG
SSSRSSNHFSTTLSENVDREAPRMPPIRPPEKRQRVPSAYNRFIKEEIQRIKAGNPAISHREAFSTAAKNWAHFPHIHFGLKL
DGNKKGKQLDQTVAGHKSNGYF*
>Brassica napus YAB2f
MSIDISSERVCYVHCNFCTTILAVSVPYASLFTLVTVRCGHCTNLLSLNIGVSLHQSSPAPPIHQDLQQHKQHITSSVTRKEH
GSSSRSFNHFSTTLSENVEREAPRMPPIRPPEKRQRVPSAYNRFIKEEIQRIKAGNPEISHREAFSTAAKNWAHFPHIHFGLK
LDGNNKKGKQIDQSVAGQKSNGYY
>Brassica napus YAB3a
MSSMSMSSSSAPAYPPDHISSSDQLCYVHCSFCDTVLAVSVPPSSLFKTVTVRCGHCSNLLSVTVNMRALLLPSVSNIGHSFL
PSPPPPPPPNLLEEMRNGGQNINMNMMMSHHAAAHHSNESFVMATRVRSVDLQEMPRPPPANRPAPEKRQRVPSAYNRFIKEE
IQRIKAGNPDISHREAFSAAAKNWAHFPHIHFGLMPDHPPTKKANVRQQEGEEVMMGREGFYGSAANVGVTHN
>Brassica napus YAB3b
MSSMSMSSSSAPAYPPDHISSSDQLCYVHCSFCDTVLAVSVPPSSLFKTVTVRCGHCSNLLSVTVNMRALLLPSVSNIGHSFL
PSPPPPPPPNLLEEMRNGGQNINMNMMMSHHAAAHHSNESFVMATRVRSVDLQEMPRPPPANRPPEKRQRVPSAYNRFIKEEI
QRIKAGNPDISHREAFSAAAKNWAHFPHIHFGLMPDHPPTKKANVRQQEGEEVMMGREGFYGSAANVGVTHN
>Brassica napus YAB5a
MANSPTAAEQLCYIPCNFCNIVLAVSVPCSSLFDIVTVRCGHCTNLWSVNMAAALQSLSRPNFQVTPYATPEYGSSSRGHTKI
SSRISARTISEQRIVNRPPEKRQRVPSAYNQFIKEEIQRIKANNPDISHREAFSTAAKNWAHFPHIHFGLMLESNKQAKLA
>Brassica napus YAB5b
MANSPTAAEQLCYIPCNFCNIVLAVSVPCSSLFDIVTVRCGHCTNLWSVNMAAALQSLSRPNFQVTPYAMPEYGSSSRGNTKI
SSRISARTISEQRIVNRPPEKRQRVPSAYNQFIKEEIQRIKANNPDISHREAFSTAAKNWAHFPHIHFGLMLESNKQAKLA

>Brassica napus INOa
MTKMANMTTLNQLFDLPGQICHVQCGFCTTILLVSVPFTSLSMVVTVRCGHCTSLLSVNLMKASFIPLHLLTSLSHLDETEKE
EVAATTDGVEKEAWKVTQEKENSPTTLVTSSDNEDEDEDKDVSRVYQVVNKPPEKRQRAPSAYNCFIKEEIRRLKAQNPSMAH
KEAFSLAAKNWAHFPPVQNKRTASDQCFCEEDNNVLLSCNALEDHEVSNNGFRERKAQRHSIWGKSPFE
>Brassica napus INOb
MTKMANMTTLNQLFDLPGQICHVQCGFCTTILLVSVPFTSLSMVVTVRCGHCTSLLSVNLMKASFIPLHLLTSLSHLDETEKD
EVAATTDGVEEEAWKVTQEKENSPTTLVTSSDNEDEDEDKDVSRVYQVVNKPPEKRQRAPSAYNCFIKEEIRRLKAQNPSMAH
KEAFSLAAKNWAHFPPVQNKRTASDQCFCEEDNNVLLSCNALEDHEVSNNGFRERKAQRHSIWGKSPFE
>Brassica napus INOc
MTKIPNMTTLNQLFDLPGQVCHVQCGFCTTILLVSVPFTSLSMVVTVRCGHCTSLLSVNLMKASFIPLHLLTSLSHMDEKGHE
EVAATTDGVEEEAWKVNQEKENSPATLVTSSESEDEDRDVSRVYQVVNKPPEKRQRAPSAYNCFIKEEIRRLKAQNPSMAHKE
AFSLAAKNWANFPPVQNKRAASDQYFCEDDNNALLSCNALGDHDESNNGFRERKAQRHSIWGKSPFD*
>Brassica napus INOd
MTKIPNMTTLNQLFDLPGQVCHVQCGFCTTILLVSVPFTSLSMVVTVRCGHCTSLLSVNLMKASFIPLHLLTSLSHMDEKGNE
EVAATTDGGVEEEAWKVNQEKENSPATLVTSSDSEDEDRDVSRVYQVVNKPPEKRQRAPSAYNCFIKEEIRRLKAQNPSMAHK
EAFSLAAKNWANFPPVQNRRAASDQCFCEDDNNALLSCNALGDHDESNNGFRERKAQRHSIWGKSPFD
>Brassica napus CRCa
MQSFDGSEYKKGSSSSSSSSTSSDQPPSPRPPFVVKPPEKKQRLPSAYNRFMRDEIQRIKSANPEIPHREAFSAAAKNWAKYI
PNSPTSIASGASNIHGFGFGEKK
>Brassica napus CRCb
MNLEEKPTMASRVSPQAEHLYYVRCSICNTILAVGIPMKRMLDTVTVKCGHCGNLSFLTTTPPLQGHVSLTLQQMQSFGGSEY
KKGSSSSSSSSTSSDQPPSPRPPFVVKPPEKKQRLPSAYNRFMRDEIQRIKSANPEIPHREAFSAAAKNWAKYIPNSPTSITS
GASNIHGFGFGEKK
>Brassica carinata FILa
MSMSSMSSPSSAVFSPEHLSPSDHLCYVQCNFCETILAVSVPYTSMFKTVTVRCGCCTNLLSVNMRSAALPASNQLQLQLGPH
SYFNTQNILEELRDAPSNMNMMMMNQHPSMNDIPSFMNIHQQHEIPKAPPVNRPPEKRQRVPSAYNRFIKEEIQRIKAGNPDI
SHREAFSAAAKNWAHFPHIHFGLAPDNQPVKKTNMPQQEGEDNM
GMKDGFYAPAANVGVAPY
>Brassica carinata FILb
MSMSSMSSPSSAVFSQEHLSPSEHLCYVQCNFCETILAVSVPYTSLFKTVTVRCGCCTNLLSVNMRSLVLPASNQLQLQLGPQ
SYFAPQNILEELREPPSNMNMMMINQHPNVNDITSFMDLQQHHEIPKAPTANRPPEKRQRVPSAYNRFIKEEIQRIKAGNPDI
SHREAFSAAAKNWAHFPHIHFGLMPDNQPVKKTNMPQQEGEENM
GMKEGFYAPAANVGMTPY
>Brassica carinata FILc
MSMSSMSSPSSAVFSPEPLSPSDHLCYVQCNFCETILAVSVPYTSMFKTVTVRCGCCTNLLSVNMRSAALPASNQLQLGPHSY
FNTQNILEELRDAPSNMNMMMMNQHPNMNDIPSFMNIHRQHEITKAPPVNRLPEKRQRVPSAYNRFIKEEIQRIKAGNPDISH
REAFSAAAKNWAHFPHIHFGLAPDNQPVKKTNMPQQDGEDNMGM
KEGLYAPAAHVGVAPY
>Brassica carinata FILd
MSMSSMSSPSSAVFSPEHLSPSEHLCYVQCNFCETILAVSVPYTSLFKTVTVRCGCCANLLSVNMRSLVLPASNQIQLQLGPQ
SYFTPQNILEELREAPSNMNMMMINQHPNMNDIPSFMDLHQKHEIPKAPPTNRPPEKRQRVPSAYNRFIKEEIQRIKAGNPDI
SHREAFSAAAKNWAHFPHIHFGLMPDNQPVKKTNMPQQAGEENM
GMKEGFYAPAANVGMTPY
>Brassica carinata YAB2a
MSIDLSSDRVCYVHCNFCTTILAVSVPYASLFTLVTVRCGHCTNLLSLNIGVSLHQTSPTHPIHQDPQQHKQHITSSVTRKEY

GSSSRSSNHFSTTLTENVDREAPRMPPIRPPEKRQRVPSAYNRFIKEEIQRIKAGNPEISHREAFSTAAKNWAHFPHIHFGLK
LDGNMKGKQLDQTVAGQKSNGYY
>Brassica carinata YAB2b
MSIDTSSERVCYVHCNFCTTILAVSVPYASLFTLVTVRCGHCTNLLSLNIGVSLHQSSPPPIHQDLQQHKQHITSSVTRKEHG
SSSRSFNHFSTTLSENVEREVPRMPPIRPPEKRQRVPSAYNRFIKEEIQRIKAGNPEISHREAFSTAAKNWAHFPHIHFGLKL
DGNKKGKQIDQTVAGQKSNGYY
>Brassica carinata YAB2c
MSIDLSSDRLCYVHCNFCTTILAVSVPYASLFTLVTVRCGHCTNLLSLNIGVSLQQSPPTPPIHQDLQQHKQQITTSITRKEY
GSSSRSSNHFSATLSENVDREAPRMPPIRPPEKRQRLPSAYNRFIKEEIQRIKAGNPEISHREAFSTAAKNWAHFPHIHFGLK
LDGNKKGKQLDQSVAGQKSNGYY
>Brassica carinata YAB2d
MSIDLSSDRVCYVLCNFCTTTLAVSVPYASLFTLVTVRCGHCTNLLSLNIGVSLHQSSPPPIHQDLQQPKQHITSSVTRKEWG
SSSRSSNHFSTTLSENVDQEAPRMPPIRPPEKRQRVPSAYNRFIKEEIQRIKAGNPAISHREAFSTAAKNWAHFPHIHFGLKL
DGNKKASN
>Brassica carinata YAB3a
MSSMSMSSSSAPAYPPDHISSSDQLCYVHCSFCDTVLAVSVPPSSLFKTVTVRCGHCSNLLSVTVNMRALLLPSVSNIGHSFL
PSPPHLPPPNLLEEMRNGGQNINTNMMMSHQAAAHHSNESFVMATRNGRVDLQEMPRPPPANRPPEKRQRVPSAYNRFIKEEI
QRIKAGNPDISHREAFSAAAKNWAHFPHIHFGLMPDHPPTKKANVRQQEGEEVMMGREGFYGSAANVGVTHN
>Brassica carinata YAB3b
MSSMSMSSSSAPAYPPDHISSSDQLCYVHCSFCDTVLAVSVPPSSLFKTVTVRCGHCSNLLSVTVNMRALLLPSVSNIGHPFL
PSPPPPPPPSLLEEMRNGGQNINMNMMMSHHAAAHHSNESFVMATRNGSVDLQEMPRPPPANRPPEKRQRVPSAYNRFIKEEI
QRIKAGNPDISHREAFSAAAKNWAHFPHIHFGLMPDHPPTKKANEGEEAMMGREGFYGSAANVGVTHN
>Brassica carinata INOa
MPNMSTLNQLFDLPGQICHVQCGFCTTILLVSVPFASLSMVVTVRCGHCTSLLSVNLMKASFLPLHLLTSLCQLDETGKDEVA
ATTDDVEEETLKVNQEKGNSPTTLVTSSDNEDEDQDVSRVYQVVNKPPEKRQRAPSAYNCFIKEEIRRLKAQNPSMAHKEAFS
LAAKNWAHFPPVQNKRAASDQCFCEEDDNVLLSCNALEDHELSNNGFRERKAQRHSIWGKSPFE
>Brassica carinata INOb
MTTLNQLFDLPGQICHVQCGFCATILLVSVPLTSLSMVVTVRCGHCTSLLSVNLLKASFIPLHLLTSLSHLDEAGKQEAAATT
DGVEEEAWKVNQEETSPTTLVTSSDNEDEDRDVSRVYQVVNKPPEKRQRAPSAYNCFIKEEIKRLKVQNPSMAHKEAFSLAAK
NWANFPPLQNKRAASDQCFYEDDNNALLSCNALEDHEERNNGFRERKAQRHSIWGKSPFE*
>Brassica carinata INOc
MTKIPNMTTLNQLFDLPGQVCHVQCGFCTTILLVSVPFTSLSMVVTVRCGHCTSLLSVNLMKASFIPLHLLTSLSHMDEKGNE
EVAATTDGGVEEEAWKVNQEKENSPATLVTSSDSEDEDRDVSRVYQVVNKPPEKRQRAPSAYNCFIKEEIRRLKAQNPSMAHK
EAFSLAAKNWANFPPVQNRRAASDQCFCEDDNNALLSCNVLGDH
DERNNGFRERKAQRHSIWGKSPFD
>Brassica carinata INOd
MANMTTLNQLFDLPGQICHVQCGFCTTILLVSVPFTSLSMVVTVRCGHCTSLLSVNLMKASFIPLHLLTSLSHLDETEKDEVA
ATTDGVEEEAWKVTLEKENSPTTLVTSSDNEDEDEDKDVSRVYQVVNKPPEKRQRAPSAYNCFIKEEIRRLKAQNPSMAHKEA
FSLAAKNWAHFPPVQNKRTASDQCFCEEDNNVLLSCNALEDHEV
SNNGFRERKAQRHSIWGKSPFE
>Brassica carinata YAB5
MANSATAAEQLCYIPCNFCNIVLAVSVPCSSLFDIVTVRCGHCTNLWSVNMAAALQSLSRPNFQATPYATPEYGSSSRGHTKI
SSRISARAISEQRVVNRPPEKRQRVPSAYNQFIKEEIQRIKANNPDISHREAFSTAAKNWAHFPHIHFGLMLESNKQAKLA
>Brassica carinata CRC
MNLEEKPTMASRALPQAENLYYVRCSICNTILAVGIPMKRMLDTVTVKCGHCGNLSFLTTSPPLQGHVSLTLQMQSFDGSEYK

KGSSSSSSSSTSSDQPPSPRPPFVVKPPEKKQRLPSAYNRFMRDEIQRIKSANPEIPHREAFSAAAKIGLSTYPILLLPLLPE
AATSTVSDSVRRSDIPKKCLKITNARVRDRTFQRRGLS

>Cakile maritima FILa
MSMSSISSPSSAVFSPEHISPSDHLCYVQCNFCETILAVNVPYTSLFKTVTVRCGCCTNLLSVNMRSAVLPASNQLQLQLGPH
SYFNTQNMLEELRDAPSNMNTMMMNQHPNMNDIPSFMDLHQHETPKAPPVNRPPEKRQRVPSAYNRFIKEEIQRIKASNPDIS
HREAFSAAAKNWAHFPHIHFGLAPDNQLVKKTNMPQQEGEDNMMMKEGFYAPAANVGVAPY
>Cakile maritima FILb
MSMSSMSSPSSAVFSPEHLSPSEHLCYVQCNFCETILAVSVPYTSMFKTVTVRCGCCTNLLSVNMRSAALPASNQLQLQLGPH
SYFNTQNILEELRDAPSNMNMMMMNQHPNMNDIPSFMNIHQQHEIPKAPPVNRPPEKRQRVPSAYNRFIKEEIQRIKAGNPDI
SHREAFSAAAKNWAHFPHIHFGLAPDNQPVKKTNMPQQEGEDNMGMKEGFYPPVANFGVAPY
>Cakile maritima FILc
MSMSSMSSPSSAVFSPENLSPDPLSPSEQLCYVQCNYCETILAVSVPYTSMFKTVTVRCGCCTNLISVNMRSLVLPASNQLQL
QLGPHSYFTHQNILEELKEAPSNMNMMMMNQHPNMNDIPSFMDLHHQQHEIPKAPPANRPPEKRQRVPSAYNRFIKEEIQRIK
AGNPDISHREAFSAAAKNWAHFPHIHFGLAPDNQPVKKTNMPQQEGEDNMGMKEGFYAPAANVGVIPY
>Cakile maritima YAB2a
MSIDLSSERVCYVHCNFCTTILAVSVPYTSLFTLVTVRCGHCTNLLSLNIGVSLHQSSPAPPIHQDLQQHKQHITSPVTRKEY
GSSSRSYNHFSTTLSENVEREAPRMPPNRPPEKRQRVPSAYNRFIKEEIQRIKAGNPEISHREAFSTAAKNWAHFPHIHFGLK
LDGNKKGKQIDQTVAGQKSNGYY
>Cakile maritima YAB2b
MSTDLSSDRVCYVHCNFCTTILAVSVPYASLFTLVTVRCGHCTNLLSLNMGVSLHQSSPPVHQDLQQPKQHITSSVTRKEIGS
SSRSFNHFSNTLSENIDREAPRMPPIRPPEKRQRVPSAYNRFIKEEIQRIKAGNPAISHREAFSTAAKNWAHFPHIHFGLKLD
GNKKGKQSDQTVAGQKSNGYY
>Cakile maritima YAB2c
MSVDLSSDHVCYVHCNFCTTILAVNVPYASLFTLVTVRCGHCTNLLSLNIGASLHQSSPTPIHQDPQEHKQHTTSSVTRKEYG
SSSRSSSHFTTTLSESVDRDAPRMSPIRPPEKRQRVPSAYNRFIKEEIQRIKAGNPEISHREAFSTAAKNWAHFPHIHFGLKM
DGNKKGKQLDQTVAGQKSNGYY
>Cakile maritima YAB3a
MSSMSMSSSSAPPYPPDHISSSDDQLCYVHCSFCDTVLAVSVPPSSLFKTVTVRCGHCSNLLSVTVNMRALVLPSVSNTGHSF
LPSPPPPHNLFEEMRNGGQNINMSMMMSHQAAAHHSNESLVMASRNGRVDLQEMPRPPPANRPPEKRQRVPSAYNRFIKEEIQ
RIKAGNPDISHREAFSAAAKNWAHFPHIHFGLMPDHPPTKKANVRQQDGEEVMMGREGFYGSAANVGVTHN
>Cakile maritima YAB3b
MIMQVCSLSLQWPLHHMLPNCLEKQKESMSSMSMSSSSAPPYPPDHISSSDDQLCYVHCSFCDTVLAVSVPPSSLFKTVTVRC
GHCSNLLSVTVNMRALVLPSVSNTGHSFLPSPPPPPNLFEEMRNGGQNINMSMMMSHQAAAHHSNESLVMASRNGRVDLQEMP
RPPPANRPPEKRQRVPSAYNRFIK*
>Cakile maritima YAB5
VSVPCSSLFDIVTVRCGHCTNLWSVNMAAALQSLSRPNFQAITPYAMPEYGSSSRGHTKISSRISARTISEQRVVIRPPEKRQ
RVPSAYNQFIKEEIQRIKANNPDISHRQAFSTTAKNASFHFFSFSILSLQDAVAIPTAAFVGISSGTGYSRRNSDE*
>Cakile maritima INOa
MTKMPNMTTLNQLFDLPGQICHVQCGFCTTILLVSVPFTSLSMVVTVRCGHCTSLLSVNLMKASFIPLHLLTSLSHLDETGKE
GVAATTDGVEEEAWKVNQEKENSPATLVTSSDNEDEDKDVPRVYQVVNKPPEKRQRAPSAYNCFIKEEIRRLKAQNPNMTHKE
AFSLAAKNWAHFPPVQNKRSASDQCFYEEDNNALLPCNALEDHDVSNNGFRERKAQRHSIWGKSPLE
>Cakile maritima INOb
MTKMPNMTALNQLFDLPGQICHVQCGFCTTILLVSVPFTSLSMVVTVRCGHCTSLLSVNLMKASFIPLHLLTSLSHLDETGKE
EVAATTDGVEEEAWKVNQEKENTSSDNEEEDKDVPRVYQVVNKPPEKRQRAPSAYNCFIKEEIRRLKAQNPNMTHKEAFSLAA

KNWAHFPPVQNKRAASDQCFYEEDNNALLPCNALEDHDVSNNGFRERKAQRHSIWGKSPLE
>Cakile maritima INOc
MPNMTTLNQLFDLPGQICHVQCGFCTTILLVSVPFTSLSMVVTVRCGHCTSLLSVNLMKATFIPLHLLTSLSHLDEAGKEEVA
ATTNVVEEEAWKVNQEKETSPTTLVTSSDNEDEDRDVSPVYQVVNKPPEKRQRAPSAYNCFIKEEIRRLKAQNPSMAHKEAFS
LAAKNWANCPPEQNKRVASDQCLCEDDNNALLSCNALEDHEESNNGFRERKAQRHSIWGKSPFEVRVVCERREESSSIDEYVD
MDF
>Cakile maritima INOd
MTKMPNMTTLNQLFDLPGQICHVQCGFCTTILLVSVPFTSLSMVVTVRCGHCTSLLSVNLMKATFIPLHLLTSLSHLDEAGKE
EVAAKTNVVEEEAWKVNQEKETSPTTLVTSSDNEDEDRDVSPVYQVVNKPPEKRQRAPSAYNCFIK*
>Cakile maritima INOe
MTKMPNMPNMTTLNQLFDLPGQICHVQCGFCTTILLVSVPFTSLSMVVTVRCGHCTSLLSVNLMKATFIPLHLLTSLSHLDEA
GKEEVAATTNVVEEEAWKVNQEKETSPTTLVTSSDNEDEDRDVSPVYQVVNKRTFVCV*
>Cakile maritima CRCa
MNLEEKPSMPSRASPQAEHLYYVRCSICNTILAVGIPMKRMLDTVTVKCGHCGNLSFLTTSPPLQGHVSLTLQMQSFGGSEYK
KGSSSSSSSSTSSDQPPSPTPPFVVKPPEKKQRLPSAYNRFMRDEIQRIKSANPEIPHREAFSAAAKNWAKYIPNSPTSITSG
ACNINGFGFGEKK
>Cakile maritima CRCb
MNLEEKSNMASPQAEHLYYVRCSICNTVLAVGIPFKRMLDTVTVKCGHCGSLSFLTTNPPLKGHVSLSLQMMSASSGGSEYKK
RSSSSSSSSTTSDQPSSPRPPFVVKPPEKKKRLPSAYNRFMRDEIQRIKSADPEIPHRDAFSAAAKNWAKYIPNSPTSITSGG
NNIYGLGFRQKK
>Camelina sativa FILa
MSMSSMSSPSSAVFSPDHLSPSDHLCYVQCNFCDTILAVSVPYTSLFKTVTVRCGCCTTLLSVNMRLVLPASNQLQLQLGPHS
YFNSQNIMEELRDAPSNMNMMMMNQHSNMNDIPSFMDLHQQHEIPKAPPVNRPPEKRQRVPSAYNRFIKEEIQRIKAGNPDIS
HREAFSAAAKNWAHFPHIHFGLVPDNQPVKKTNMPQQEGEDNMVMKDGFYAPAANVGVTPY
>Camelina sativa FILb
MSMSSMSSPSSAVFSPDHLSPSDHLCYVQCNFCDTILAVSVPYTSLFKTVTVRCGCCTTLLSVNMRLVLPASNQLQLQLGPHS
YFNSQNIMEELRDAPSNMNMMMMNQHSNMNDIPSFMDLHQQHEIPKAPPVNRPPEKRQRVPSAYNRFIKEEIQRIKAGNPDIS
HREAFSAAAKNWAHFPHIHFGLVPDNQPVKKTNMPQQEGEDNNMVMKDGFYAPAANVGVTPY
>Camelina sativa FILc
MSMSSMSSPSSAVFSPDHLSPSDHLCYVQCNFCDTILAVSVPYTSLFKTVTVRCGCCTTLLSVNMRLVLPASNQLQLQLGPHS
YFNSQNIMEELRDAPSNMNMMMMNQHSNMNDIPSFMDLHQQHEIPKAPPVNRPPEKRQRVPSAYNRFIKEEIQRIKAGNPDIS
HREAFSAAAKNWAHFPHIHFGLVPDNQPVKKTNMPQQEGEDNMVMKDGFYAPAANVGVTPY
>Camelina sativa YAB2a
MSVDLSSERVCYVHCSFCTTILAVSVPYASLFTLVTVRCGHCTNLLSLNIGVSLHQTSPPPINQDLQPYKQHITSSVTRKDFG
SSSRSTNNISTTLSENIDRDAPRMPPIRPPEKRQRVPSAYNRFIKEEIQRIKACNPEISHREAFSTAAKNWAHFPHIHFGLKL
DGNKKGKQLDQTVASQKSNGYC
>Camelina sativa YAB2b
MSVDLSSERVCYVHCSFCTTILAVSVPYASLFTLVTVRCGHCTNLLSLNIGVSLHQTSTPPINQDLQPYKQHITSSVTRKDFG
SSSRSANNISTTLSENIDREAPRMPPIRPPEKRQRVPSAYNRFIKEEIQRIKACNPEISHREAFSTAAKNWAHFPHIHFGLKL
DGNKKGKQLDQTVASQKSNGYC
>Camelina sativa YAB2c
MSVDLSSERVCYVHCSFCTTILAVSVPYASLFTLVTVRCGHCTNLLSLNIGVSLHQTSPTPINQDLQPYKQHITSSVTRKDFG
SSSRSTNNISTTLSENIDREAPRMPPIRPPEKRQRVPSAYNRFIKEEIQRIKACNPEISHREAFSTAAKNWAHFPHIHFGLKL
DGNKKGKQLDQTVASQKSNGYC
>Camelina sativa YAB3a

MCDCRQVSVPPSSLFKTVTVRCGHCSNLLSVTVNMRTLLLPSVSNIGHSFIPPPPPPPNLLEEMRSGGQNINMNMMMSHHGAA
HHPNESLAMAARNGLQEMPRPPPANRPPEKRQRVPSAYNRFIKEEIQRIKAGNPDISHREAFSAAAKNWAHFPHIHFGLMADH
PPTKKANVRQQQEGEDGMMGSREGFYGSAANVGVTNN
>Camelina sativa YAB3b
MSSMSMSSSSAPAYPPDHISSTDQLCYVHCSFCDTVLAVSVPPSSLFKTVTVRCGHCSNLLSVTVNMRTLLLPSVSNIGHSFI
PPPPPPPNLLEEMRSGGQYINMNMMMSHHGAAHHPNESLAMAARNGLQEMPRPPPANRPPEKRQRVPSAYNRFIKEEIQRIKA
GNPDISHREAFSAAAKNWAHFPHIHFGLMADHPPTKKANVRQQQQEGEDGMMGSREGFYGSAANVGVTNN
>Camelina sativa YAB3c
MSSMSMSSSSAPAYPPDHISSTDQLCYVHCSFCDTVLAVSVPPSSLFKTVTVRCGHCSNLLSVTVNMRTLLLPSVSNIGHSFI
PPPPPPPNLLEEMRSGGQNINMNMMMSHHGAAHHPNESLAMAARNGLQEMPRPPPANRPPEKRQRVPSAYNRFIKEEIQRIKA
GNPDISHREAFSAAAKNWAHFPHIHFGLMADHPPTKKANVRQQQEGEDGMMGSREGFYGSAANVGVTNN
>Camelina sativa YAB5a
MANSVMATEQLCYIPCNFCNIVLAVSVPCSSLFDIVTVRCGHCTNLWSVNMAAALQSLSRPNFQATNNAVPEYGSSSRGHTKI
PSRISTRTKTEQRIVNRSTEKRQRVPSAYNQFIKEEIQRIKANNPDISHREAFSTAAKNWAHFPHIHFGLMLESNKQAKLA*
>Camelina sativa YAB5b
MANSVMATEQLCYIPCNFCNIVLAVSVPCSSLFDIVTVRCGHCTNLWSVNMAAALQSLSRPYFQATNNAVPEYGSSSRGHTKI
PSRISTRTKTEQRILNRSTEKPQRVHSAYNQFIKEEIQRIKANNPDISHREAFSTAAKNWAHFPHIHFGLMLESNKQAKLA
>Camelina sativa YAB5c
MANSMMATEQLCYIPCNFCNIVLAVSVPCSSLFDIVTVRCGYCTNLWSVNMAAALQSLSRPNFQATNNAVPEYGSSSRGHTKI
PSRISTRTKTEQRIVNRSTEKRQRVPSAYNQFIKEEIQRIKANNPDISHREAFSTAAKNWAHFPHIHFGLMLESNKQAKLA
>Camelina sativa INOa
MTKIPNMTTTLNNLFDLPGQICHVQCGFCTTILLVSVPYTSLSMVVTVRCGHCTSLLSVNLMKASFIPLHLLASLSNLDEAGK
EEVAATDGVEEEALKVNSLEKENSPTTLVSSSDNEDEDVSRVYQVVNKPPEKRQRAPSAYNCFIKEEIRRLKAQNPSMAHKEA
FSLAAKNWAHFPPVHNKRAASDHCFCVEDNNAIPSCNALEDHEESNNGFRERKAQRHSIWGKSPFE
>Camelina sativa INOb
MTKIPNMTTTLNHLFDLPGQICHVQCGFCTTILLVSVPYTSLSMVVTVRCGHCTSLLSVNLMKASFIPLHLLASLSNLDEAGK
EEVAATDGVEEEALKVNSQEKENSPTTLVSSSDNEDEDVSRVYQVVNKPPEKRQRAPSAYNCFIKEEIRRLKAQNPSMAHKEA
FSLAAKNWAHFPPVNNKRATSDHCFCEEDNNAIPPFNALEDHEESNNGFRERKAQRHSIWGKSPFE*
>Camelina sativa INOc
MTKIPNMTTTLNHLFDLPGQICHVQCGFCTTILLVSVPYTSLSMVVTVRCGHCTSLLSVNLMKASFIPLHLLASLSNLDEAGK
EEVAATDGVEEEALKVNSQEKENSPTTLVSSSDNEDEDVSRVYQVVNKPPEKRQRAPSAYNCFIKEEIRRLKAQNPSMAHKEA
FSLAAKNWAHFPPVNNKRATSDYCFCEEDNNAIPPFNALEDHEESNNGFRERKAQRHSIWGKSPFE
>Camelina sativa CRCa
MNLEEKPTMTSKASSQAEHLYYVRCSICNTILAVGIPLKRMLDTVTVKCGHCGNLSFLTTSPPLQGHVSLTLQMQSFGGSEYK
KGSSSSSSSSTSSDQPPPPTPPFVVKPPEKKQRLPSAYNRFMRDEIQRIKSANPEIPHREAFSAAAKNWAKYIPNSPTSITSG
GNNIHGLAFGEKK
>Camelina sativa CRCb
MNLEEKPTMTSKASSQAEHLYYVRCSICNTILAVGIPLKRMLDTVTVKCGHCGNLSFLTTSPPLQGHVSLTLQMQSFGGSEYK
KGSSSSSSSSTSSDQPPPPTPPFVVKPPEKKQRLPSAYNRFMTDEIQRIKSANPEIPHREAFSAAAKNWAKYIPNSPTSITSG
GNNIHGLGFGEKK
>Camelina sativa CRCc
MNLEEKPPTMTSKASSQAEHLYYVRCSICNTILAVGIPLKRMLDTVTVKCGHCGNLSFLTTSPPLQGHVSLTLQMQSFGGSEY
KKGSSSSSSSSTSSDQPPSPTPPFVVKPPEKKQRLPSAYNRFMRDEIQRIKSANPEIPHREAFSAAAKNWAKYIPNSPTSITS
GGNNIHGLGFGEKK
>Capsella grandiflora FIL

MSMSSMSSPSSAVFSPDHLSPSDHLCYVQCNFCDTILAVSVPYTSLFKTVTVRCGCCTTLLSVNMRLVLPASNQLQLQLGPHS
YFNSQNMLEELRDAPSNMNMMMMNQHPNMNDMPSFMDLHQQHEIPKAPPVNRPPEKRQRVPSAYNRFIKEEIQRIKAGNPDIS
HREAFSAAAKNWAHFPHIHFGLVPDNQPVKKTNMPQQEGEDNMVMKDGFYAPAANVGVTPY*
>Capsella grandiflora YAB2
MSVDLSSERVCYVHCNFCTTILAVSVPYASLFTLVTVRCGHCTNLLSLNIGVSLHQSSPPPIHQDLQPHKQHITSSVTRKDYG
SSSRSTNHMSTTMSENIDREAPRMPPIRPPEKRQRVPSAYNRFIKEEIQRIKACNPEISHREAFSTAAKNWAHFPHIHFGLKL
DGNKKGKQLDQTVASQKSNGYC*
>Capsella grandiflora YAB3
MSSMSMSSSSAPAYPPDHISSSDQLCYVHCNFCDTVLAVSVPPSSLFKTVTVRCGHCSNLLSVAVNMRALLLPSVSNIGHSFI
SPTPPPPPNLLEEMRSGGQNINMNMIMSHHHGGAHHPNESLAMGTRNGRVDHHLQEMPRPPPANRPPEKRQRVPSAYNRFIKE
EIQRIKAGNPDISHREAFSAAAKNWAHFPHIHFGLMADHPPTKKANVRQQEGEDVMMGSSREGFYGSAANVGVTHN*
>Capsella grandiflora YAB5
MANSAMSSEQLCYIPCNFCNIVLAVSVPCSSLFDIVTVRCGHCTNLWSVNMAAALQSLSRPNFQATNYAVPDYGSTSRGHTKT
PSRISTRTITEQRIVNRPPEKRQRVPSAYNQFIKEEIQRIKANNPDISHREAFSTAAKNWAHFPHIHFGLMLESNKQAKLA*
>Capsella grandiflora INO
MTKIPNMTTTLNHLFDLPGQICHVQCGFCTTILLVSVPFTSLSMVVTVRCGHCSSLLSVNLMKASFIPLHLLASLSHLDEAGK
EEVAATTGVEEEEALKVNSQEKENSPTTLVSSSDNEDEDVTRVYQVVNKPPEKRQRAPSAYNCFIKEEIRRLKSQNPSMAHKE
AFSLAAKNWAHFPPVLNKRAASSDHCYCEDQDNNAIPPCNALEDHEESNNGFRERKAQRHSIWGKSPFE*
>Capsella grandiflora CRC
MNLEEKPTMTSKASPQAEHLYYVRCSICNTILAVGIPLKRMLDTVTVKCGHCGNLSFLTTSPPLQGHVSLTLQMQSFGGSEYK
KGSSSSSSSSTSSDQPPSPTPPFVVKPPEKKQRLPSAYNRFMRDEIQRIKSANPEIPHREAFSAAAKNWAKYIPNSPTSLTSG
GHNIHGLGFGEKK*
>Capsella rubella FIL
MSMSSPSSAVFSPDHLSPSDHLCYVQCNFCDTILAVSVPYTSLFKTVTVRCGCCTTLLSVNMRLVLPASNQLQLQLGPHSNFN
SQNMLEELRDAPSNMNMMMMNQHPNMNDMPSFMDLHQQHEIPKAPPVNRPPEKRQRVPSAYNRFIKEEIQRIKAGNPDISHRE
AFSAAAKNWAHFPHIHFGLVPDNQPVKKTNMPQQEGEDNMVMKDGFYAPAANVGVTPY
>Capsella rubella YAB2
MSVDLSSERVCYVHCNFCTTILAVSVPYASLFTLVTVRCGHCTNLLSLNIGVSLHQSSPPPIHQDLQPHKQHITSSVTRKDYG
SSSRSTNHMSTTMSENIDREAPRMPPIRPPEKRQRVPSAYNRFIKEEIQRIKACNPEISHREAFSTAAKNWAHFPHIHFGLKL
DGNKKGKQLDQTVASQKSNGYC
>Capsella rubella YAB3
MSSMSMSSSSAPAYPPDHISSSDQLCYVHCNFCDTVLAVSVPPSSLFKTVTVRCGHCSNLLSVAVNMRALLLPSVSNIGHSFI
SPTPPPPPNLLEEMRSGGQNINMNMIMSHHHGGAHHPNESLAMGTRNGRVDHHLQEMPRPPPANRPPEKRQRVPSAYNRFIKE
EIQRIKAGNPDISHREAFSAAAKNWAHFPHIHFGLMADHPPTKKANVRQQEGEDVMMGSSREGFYGSAANVGVTHN
>Capsella rubella YAB5
MANSAMSSEQLCYIPCNFCNIVLAVSVPCSSLFDIVTVRCGHCTNLWSVNMAAALQSLSRPNFQATNYAVPDYGSTSRGHTKT
PSRISTRTITEQRIVNRPPEKRQRVPSAYNQFIKEEIQRIKANNPDISHREAFSTAAKNWAHFPHIHFGLMLESNKQAKLA
>Capsella rubella INO
MTKIPNMTTTLNHLFDLPGQICHVQCGFCTTILLVSVPFTSLSMVVTVRCGHCSSLLSVNLMKASFIPLHLLASLSHLDEAGK
EEVAATTGVEEEEALKVNSQEKENSPTTLVSSSDNEDEDVTRVYQVVNKPPEKRQRAPSAYNCFIKEEIRRLKSQNPSMAHKE
AFSLAAKNWAHFPPVLNKRAASSDHCYCEDQDNNAIPPCNALEDHEESNNGFRERKAQRHSIWGKSPFE
>Capsella rubella CRC
MNLEEKPTMTSKASPQAEHLYYVRCSICNTILAVGIPLKRMLDTVTVKCGHCGNLSFLTTSPPLQGHVSLTLQMQSFGGSEYK
KGSSSSSSSSTSSDQPPSPTPPFVVKPPEKKQRLPSAYNRFMRDEIQRIKSANPEIPHREAFSAAAKNWAKYIPNSPTSLTSG
GHNIHGLGFGEKK

>Caulanthus amplexicaulis FILa
MSMSSMSSPSSAVFSPEHLSPSEHLCYVQCKFCETILAVSVPYTSLFKTVTVRCGCCTNLLSVNMRLVLPASNQLQLQLGPHS
YFNPQNILEELRDAPSNMNMMMMNQHPNMNDIPSFMDLHQQHEIPKAPPVNRPPEKRQRVPSAYNRFIKEEIQRIKAGNPDIS
HREAFSAAAKNWAHFPHIHFGLVPDNQPVKKTNMPQQGDDIMGMKEGFYAPAANVGETPY
>Caulanthus amplexicaulis FILb
MSMSSMSSPSSAVFSPEHLSPSDHICYVQCNFCETILAVSVPYTSLFKTVTVRCGCCTNLLSVNMRTAVLPASNQLQLQLGPH
SYFNTQNILEELRDAPSNMNMMMMNQHPNMNDIPSFIDLHQQHEIPKAPPVNRPPEKRQRVPSAYNRFIKEEIQRIKAGNPDI
SHREAFSAAAKNWAHFPHIHFGLVPDNQPVKKTNMPQQEGDDNMGMKEGFYAPAANVGVAPY
>Caulanthus amplexicaulis YAB2a
MSIDLSSERVCYVHCNFCTTILAVSVPYASLFTLVTVRCGHCTNLLSLNIGVSLHQSSPPPIHQDLQHRQHITSSVTIARKEC
GSSSRSSNHFSTTLSENVDREAPRMPPIRPPEKRQRVPSAYNRFIKEEIQRIKAGNPEISHREAFSTAAKNWAHFPHIHFGLK
LDGNKKGKQLDQTVSGQKSNGYY
>Caulanthus amplexicaulis YAB2b
MSIDLASESVCYVHCNFCTTILAVSVPYASLFTLVTVRCGHCTNLLSLNIGVSLHQSSPPPIHQELQHKQHITSAVTRKECGS
SSRSSNHFSSTFSENVDREAPRMPPIRPPEKRQRVPSAYNRFIKEEIQRIKAGNPAISHREAFSTAAKNWAHFPHIHFGLKLD
GNKKGKQLDQTVAGQKSNGYY
>Caulanthus amplexicaulis YAB3a
MSSMSMSSSSAPAYPPDHISSSEQLCYVHCNFCDTVLAVSVPPSSLFKTVTVRCGHCSNLLSVTVNMRALLLPSVSNLGHSFL
PPPPPPPLLLEEMRNGGQNINMNMMMSHHGAVHHSNESLVMATRNGRVDLQELPRPAPANRPPEKRQRVPSAYNRFIKEEIQR
IKAGNPDISHREAFSAAAKNWAHFPHIHFGLMPDHPPTKKANVRQQEGEDVMMGREGFYGSAANVGVTHT
>Caulanthus amplexicaulis YAB3b
MSSMSMSSSSAPAYSPDHISSTDQLGYVHCSFCDTVLAVSVPPSSLFKTVTVRCGHCSNLLSVTVNMRALLLPSVSNLGHSLL
PPPPPPPPPNFLEEMRNGGQIINMNMMMSHQAAAHHSNESLVMATRNGRVDQQEMHRPPPANRPAPEKRQRVPSAYNRFIKEE
IQRIKAGNPDISHREAFSAAAKNWAHFPHIHFGLIPDHPPTKKANVRQQERM
>Caulanthus amplexicaulis YAB5a
MANSATAAEQLCYIPCNFCNIVLAVSVPCSSLFDIVTVRCGHCTNLWSVNMAAALQSLSRPNFQATNYAMPEYGSSSRGYTKI
PSRLSARTTNEQRVVNRPPEKRQRVPSAYNQFIKEEIQRIKANNPDISHREAFSTAAKNWAHFPHIHFGLMLESNKQAKLA
>Caulanthus amplexicaulis YAB5b
MADSTMATEQLCYIPCNFCNIVLAVSVPCSSLFEIVTVRCGHCTNLWSVNMAAALQSLSRPNFQAINYAMPEYGSSSRGHSKI
PSRISARTITEQRVVNHPPEKRQRVPSAYNQFIKEEIQRIKANNPDISHREAFSTAAKNWAHFPHIHFGLMLESNKQAKLA

>Caulanthus amplexicaulis INOa
MTKMQNMTTLNQLFDLPGQICHVQCGFCTTILLVSVPFTSLSMVVTVRCGHCTSLISVNLMKASFIPFHLFTSLSHLDEVPAG
REEVAATTGDVEEEACKMTQEKENSPTTLVTSSDNEDEDRDVSRVYQVVNKPPEKRQRAPSAYNCFIKEEIRRLKAQNPSMAH
KEAFSLAAKNWAHFPPVQNKRANSDQCFCENDNNALLSCNALEDHEESNNGFRERKAQRHSIWGKSPFE
>Caulanthus amplexicaulis INOb
MTKKPNMTTLNQLFDLPGQICHVQCGFCTTILLVSVPFTSLSMVVTVRCGHCTSLLSVNLMKASFIPLHLLTSLSHLDETGKE
EVAATTDGVEEEAWKVNREKENSPTTLVTSSDNEDEDKDVSRVYQVVNKPPEKRQRAPSAYNCFIKEEIRRLKAQNPSMAHKE
AFSLAAKNWAHFPPVQNKRAASDQCFCEEDNNALISCNALEDHEESNNGFRERKAQRHSIWGKSPFE
>Caulanthus amplexicaulis CRCa
MNLEEKPTMASRAASQAEHLYYVRCSICNTILAVGIPLKRMLDTVTVKCGHCGNLSFLTTSPPLRGHVSLTLQMQSFGGSEYK
KGSSSSSSSSTSSEQPPSPRPPFVVKPPEKKQRLPSAYNRFMRDEIQRIKSANPEIPHREAFSAAAKNWAKYIPNSPTSTTSG
ANNINGFVFGEKK
>Caulanthus amplexicaulis CRCb
MNHEEKSAMASRASPQAEHLYYVRCSICNTILAVGIPMKRMLDTVTVKCGHCGNLSFLTTSPPLQGHVSLTLQMQSFGGNGYK

KGSSSSSSSSTSSDQPPSPRPPFVVKPPEKKQRLPSAYNRFMRDEIQRIKSANPEIPHREAFSAAAKNWAKYVPISPASITSG
ASNINGFGFGEKK

>Crambe hispanica FILa
MSMSSPSSAVFSPEHLSPSEHLCYVQCNFCETILAVSVPYTSLFKTVTVRCGCCTNLLSVNMRSLVLPASNQLQLQLGPQSFF
TPQNILEELREAPSNMNMMMNQHPNMNDIPSFMDLHQQHEIPKAPPANRPPEKRQRVPSAYNRFIKEEIQRIKAGNPDISHRE
AFSAAAKNWAHFPHIHFGLMPDNQPVKKTNMPHQEDEENMGMKEGFYAPAANVGMTPY
>Crambe hispanica FILb
MSMSSMSSPSSAVFSPENLSPDPLSPSEQLCYVQCNYCETILAVSVPYTSMFKTVTVRCGCCTNLISVNMRSLVLPASNQLQL
QLGPHSFFTPQNILEELKDAPSNMNMMMMNQHPNMTDIPSFMDLHHQHEIPKAPPVNRPPEKRQRVPSAYNRFIKEEIQRIKA
GNPDISHREAFSAAAKNWAHFPHIHFGLAPDNQPVKKTNMPQQEGEDNMGMREGFYPPAANVAVIPY
>Crambe hispanica FILc
MSMSYMSSPSSAVFSPEHLSPSDHLCYVQCNFCETILAVSVPYTSMFKTVTVRCGCCTNLLSVNMRSAALPASNQLQLQLGPH
SYFNTQNILDELRDAPSNMNMMMMNQHPNMNDIPSFMNIHQQHEIPKAPPVNRPPEKRQRVPSAYNRFIKEEIQRIKAGNPDI
SHREAFSAAAKNWAHFPHIHFGLAPDNQPVKKTNMPQQEGDDNMGMKEGFYAPAANVGVAPY
>Crambe hispanica YAB2a
MSIDISSERVCYVHCNFCTTILAVSVPYASLFTLVTVRCGHCTNLLSLNIGVSLHQSSPPPIHQDLQQHKQHITSSVTRKEHG
SSSRSFNHFSTTLSENVEREAPRMPPIRPPEKRQRVPSAYNRFIKEEIQRIKAGNPEISHREAFSTAAKNWAHFPHIHFGLKL
DGNKKGKQIDQTVAGQKSNGYY
>Crambe hispanica YAB2b
MSIDLSSDRVCYVHCNFCTTILAVSVPYASMFTLVTVRCGHCTNLLSLNIGVSLHQTSPTPIHQDPQHKQQITSSVTRKEYGS
SSRSSNHFSTTLSENVDREAPRMLPIRPPEKRQRVPSAYNRFIKEEIQRIKAGNPEISHREAFSTAAKNWAHFPHIHFGLKLD
GNKKGKQLDQTVAGQKSNGYY
>Crambe hispanica YAB2c
MSIDLSSDRVCYVHCNFCTTTLAVSVPYASLFTLVTVRCGHCTNLLSLNIGVSLHQSSPPPTHQELQPKQHITSSVTRKEWGS
SSRSSNHFSATLSGNVDRDVPRTPPIRPPEKRQRVPSAYNRFIKDEIQRIKAGNPAISHREAFSTAAKNWAHFPHIHFGLKLD
GNKKGKQLDQTVAGQKSNGYY
>Crambe hispanica YAB3
MSSMSMSSSSAPAYPPDHISSLDQLCYVHCSFCDTVLAVSVPPSSLFKTVTVRCGHCSNLLSVTVNMRALLLPSVSNIGHSFL
PSPPPPPPNLLEEMRNGGQNINMNMMMSHQAAAHHSNESLVMATRNGRVDLQEMPRPPPANRPPEKRQRVPSAYNRFIKEEIQ
RIKAGNPDISHREAFSAAAKNWAHFPHIHFGLMPDHPPTKKANVRQQEGEEVMMGREGFYGSAANVGVTHN
>Crambe hispanica YAB5
MANSTTAAEQLCYIPCNFCNIVLAVSVPCSSLFDIVTVRCGHCTNLWSVNMAAALQSLSRPNFQATPYAMPEYGSSSRGHTKI
SSRISARTITEQRVVNRPPEKRQRVPSAYNQFIKEEIQRIKANNPDISHREAFSTAAKNWAHFPHIHFGLMLESNKQAKLA
>Crambe hispanica INOa
MSKVPNMTTLNQLFDLAGQICHVQCGFCTTILLVSVPFSSLSMVVTVRCGHCTSLLSVNLTKASFIPLHLLTSLSHLGETGKE
EVAATTDVVEEEAWKANQEENSPTTLVTSSDNEDEDKDVSRVYQVVNKPPEKRQRAPSAYNCFIKEEIRRLKAQNPSMAHKEA
FSLAAKNWAHFPPVQNKRTASDQCFLEEDNNALLSCNALENHEVSNNCFRERKAQRHSIWGKSPFE
>Crambe hispanica INOb
MTTLNQLFDLPGQICHVQCGFCNTILLVSVPFTSLPMVVTVRCGHCTSLLSVNLMKASFIPLHLLTSLSHPDEAGKEEVAATT
DGVEEEAWNVNQEKETSPTTLVTSSDNEDEDRNVSRVYQVVNKQAPEKRQRAPSAYNCFIKEEIRRLKAQNPSMVHKDAFSLA
AKNWANFPPVQNKRAASDQCFCEDDNNALLSCNALEDHEESNNGFRERKAQRHSIWGKSPFE
>Crambe hispanica CRCa
MNLEEKPTMASRASPQAEHLYYVRCSICNTILAVGIPMKRMLDTVTVKCGHCGNLSFLTTTPPLQGHVSLTLQMQSFGGSEYK
KGSSSSSSSSTSSDQPPSPRPPFVVKPPEKKQRLPSAYNRFMRDEIQRIKSANPEIPHREAFSAAAKNWAKYIPNSPTSITSG

GSNINGFGFGEKK

>Crambe hispanica CRCb

MNLKEKSTMASPQAEHLYYVRCNICNTILAVGIPFKRMLDTVTVKCGHCGSLSFLTTSHPLKGNVSLSLQMRSSSSGGSGYKK

GSSSSSSSSTSIDQPSSPRPPFVVKPPEKKKRLPSAYNHFMREEIQRIKRVDPEIPHREAFSAAAKNWAKYIPNSPTSITYGA

NGFRFREKK

>Descurainia sophioides FIL

MSMSSPSSAVFSPDHLSPSEHLCYVQCNFCETILAVSVPYTSLFKTVTVRCGCCTNLLSVNMRTHFLPASNPLQLQLGPHSYF

NSQNILEELRDSPSNMMMMNQHPNMNDIPSFMDLHQQHEIPKAPPVNRPPEKRQRVPSAYNRFIKEEIQRIKAGNPDISHREA

FSAAAKNWAHFPHIHFGLVPDNQPVKKTNMPQQEGEENMVMKEGFYAPAANVGVTPY

>Descurainia sophioides YAB2

MSVDLSSERVCYVHCSFCTTILAVSVPYASLFTLVTVRCGHCTNLLSLNIGVSLHQTSPPPIHQDLQPHKQHITSSVTRKDCA

SSSSRSTNNLSENIDREAPRMPPIRPPEKRQRVPSAYNRFIKEEIQRIKACNPEISHREAFSTAAKNWAHFPHIHFGLKLDGN

KKGKQLDQSVAGQKSNGYY

>Descurainia sophioides YAB3

MSMSSSSAPAFSPDHISSLDQLCYVHCSFCDTVLAVSVPPSSLYKTVTVRCGHCSNLLSVTVSMRTLLLPSVSNHGHSFIPPP

PPPNLLEEMRSGGQNINMNLMMSHHQGAHHPNESLVMATRNGRVDHLQEMPRPPPANRPPEKRQRVPSAYNRFIKEEIQRIKA

SNPDISHREAFSAAAKNWAHFPHIHFGLMADHPPTKKANVRQQEGEDVMMGREGFYGSTANVGVTHN

>Descurainia sophioides YAB5

MANSATTSEQLCYIPCNFCNIVLAVSVPCSSLFDIVTVRCGHCTNLWSVNMAAALQSLSRPNFQATNYALPEYGSSSRSHTKI

PSRISNRNITEQRVVNRPPEKRQRVPSAYNQFIKEEIKRIKANNPDISHREAFSTAAKNWAHFPHIHFGLMLESNKQAKLA

>Descurainia sophioides INO

MTRKPNMTTLNHLFDLPGQICHVQCGFCTTILLVSVPFTSLSMVVTVRCGHCTSLLSVNLMKASFIPLHLLTSLSGHLDEAGK

EEVVAIDGVEEEAWKVNQEKENSPTTLVTSSDNEDEDVSRVYQVVNKPPEKRQRAPSAYNCFIKEEIRRLKAQNPSMAHKEAF

SLAAKNWAHFPPVHNKRGASDLCFCEEDGNAELPCNNALEDHEESNNGFRERKAQRHSIWGKSPFE

>Descurainia sophioides CRC

MNLEEKPTMTSRASPQAEHLYYVRCSICNTILAVGIPLKRMLDTVTVKCGHCGNLSFLTTSPPLQGHVSLTLQMQSFGGSEYK

KGSSSSSSSSTSSDQPPSPTPPFVVKPPEKKQRLPSAYNRFMRDEIQRIKSANPEIPHREAFSAAAKNWAKYIPNSPTSLTSG

LNNINGLGFGENK

>Diptychocarpus strictus FIL

MSMSSMSSPSSAVFSPDHLSPSPSEHLCYVQCNFCDTILAVNVPYTSLFKMVTVRCGCCTNLLSVNMRSLVLPPSNQLQLQLG

PHSYYNPQNILEELRETPTNMNMMMMNQQHPNMMNDMSTSFMDLHQQQHEIPKAPPVNRPPEKRQRVPSAYNRFIKEEIQRIK

AGNPDISHREAFSAAAKNWAHFPHIHFGLVPENQPVKKTNLPQQEGEDNNNNNNMVMKEGYYAPSANNLGVTPY

>Diptychocarpus strictus YAB2

MSMDLSSERVCYVHCNFCTTILAVSVPYASLFTFVTVRCGHCTNLLSLNIGVSLHQTSSTPPNIHHHQDHQIHQPHKQHMTSS

VTRKDSGSSSRSNINHFSSTLSENVDREAPRMPPIRPPEKRQRVPSAYNRFIKEEIQRIKASNPEISHREAFSTAAKNWAHFP

HIHFGLKLDGNKKGKQLEHTVAAQKSSNGYY

>Diptychocarpus strictus YAB3

MSSSMSMSSSSLAPPAYPPDHHISSSSSSSLDQLCYVHCRFCDTVLAVSVPPSSVFKTVTVRCGHCSNLLSVTVSMRALLLPS

SSVSNHHGHGHSFLPPPPPSPPNLLEEMRNGGQNINMNMNMMMNHHHHHHHANESLVMAARNGRVDLQQEMPRAPPQANRPPE

KRQRVPSAYNRFIKEEIQRIKAGNPDISHREAFSAAAKNWAHFPHIHFGLMPDHPPSKKPNVRQQEGEDVNVMMGREGFYGSA

TNFGLTHN

>Diptychocarpus strictus YAB5

MATASEQLCYIPCNFCNIVLAVSVPCSSLYDIVTVRCGHCTNLWSVNMAAALQSLSRPNFQATNYGIMPEYGSSSSRGHTKIP

SRISTRTITEQRVVNRPPEKRQRVPSAYNQFIKEEIQRIKANNPDISHREAFSTAAKNWAHFPHIHFGLMLESNKQAKLA

>Diptychocarpus strictus INO

MTTLNHLFDLPGQICHVQCGFCTTILLVSVPFTSMSMVVTVRCGHCTSLLSVNLMKASFIPLHLLTSLSHLDEEVAATDGVEE
EACKKDMSPTTLVTSSDNEDEDKDVSRVYQVVNKPPEKRQRAPSAYNCFIKEEIRRLKAQNPSMAHKEAFSLAAKNWAHFPPV
HNKRAASDQCYCEEDNNVVLPCNALEDHEESNNGFRERKAQRHSIWGKSPFE
>Diptychocarpus strictus CRC
MNLEEKPTMASSRASPQAEHLYYVRCSICNTILAVGIPMKRMLDTVTVKCGHCGNLSFLTTSPPLQGHVSLTLQMQSFGGNEY
KKGSSSSSSSSSTSSDQPPSPTPPFVVKPPEKKQRLPSAYNRFMRDEIQRIKSANPEIPHREAFSAAAKNWAKYIPNSPTSGGN
NIHGFGFGEKK*
>Eruca vesicaria FILa
MSMSSMSSPSSAVFSPEHLSPSDHLCYVQCNFCETILAVSVPYTSMFKTVTVRCGCCTNLLSVNMRSAALPASNQLQLQLGPH
SYFNTQNILEELRDAPSNVNMMMMNQHANMNDIPSFMNIHQQHEIPKAPPVNRPPEKRQRVPSAYNRFIKEEIQRIKAGNPDI
SHREAFSAAAKNWAHFPHIHFGLAPDNQPVKKTNMPQQEGEDNLGMKEGFYAPAANVGVAPY
>Eruca vesicaria FILb
AVFSPEHLSPSDHLCYVQCNFCETILAVSVPYTSLFKTVTVRCGCCTNLLSVNMRSAALPASNQLQLQLGPHSYFNTQNILEE
LRDAPSNVNMMMMNQHANMNDIPSFMNIHQQHEIPKAPPVNRPPEKRQRVPSAYNRFIKEEIQRIKAGNPDISHREAFSAAAK
NWAHFPHIHFGLAPDNQPVKKTNMPQQEGEDNLGMKEGFYAPAANVGVAPY
>Eruca vesicaria FILc
MSMSSMSSPSSAVFSPENLSPDPLSPSEQLCYVQCNYCETILAVSVPYTSMFKTVTVRCGCCTNLISVNMRSLVLPASNQLQL
QLGPHSYFTPQNILEELKEAPTNMNMMMMNQHPNMNDIPSFMDLHQQHEIPKAPPVNRPPEKRQRVPSAYNRFIKEEIQRIKA
GNPDISHREAFSAAAKNWAHFPHIHFGLAPDNQPVKKTNMPQQEGEDNMGMREGFYAPAANVGVIPY
>Eruca vesicaria FILd
MSVSSMSSPSSAVFSPEHLSPSDHLCYVQCNFCETILAVSVPYTSLFKTVTVRCGCCTNLLSVNMRSLVLPASNQLQLQLGPQ
SYFTPQNVLEELREAPSNMNMMLMNQQPNLNDIASFMDLHQQQEIPKAPPANRPPEKRQRVPSAYNRFIKEEIQRIKAGNPDI
SHREAFSAAAKNWAHFPHIHFGLMPDNQPVKKPNMPQQEGEENMGMKEGFYAPAANVVMTPY
>Eruca vesicaria FILe
MSVSSMSSPSSAVFSPEHLSPSDHLCYVQCNFCETILAVSVPYTSLFKTVTVRCGCCTNLLSVNMRSLVLPASNQLQLQLGPQ
SYFTPQNVLEELREAPSNMNMMLMNQQPNLNDIASFMDLHPQQEIPKAPPANRPPEKRQRVPSAYNRFIKEEIQRIKAGNPDI
SHREAFSAAAKNWAHFPHIHFGLMPDNQPVKKPNMPQQEGEENMGMKEGFYAPAANVVMTPY
>Eruca vesicaria YAB2a
MSIDLSSERVCYVHCNFCTTILAVSVPYASLFTLVTVRCGHCTNLLSLNIGVSLHQSSPAPPIHQDLQQPKQHMTSSATRKEW
GSSSRSSNHFSTTLSENVDREAPRMPPIRPPEKRQRVPSAYNRFIKEEIQRIKAGNPAISHREAFSTAAKNWAHFPHIHFGLK
LDGNKKGKQLDQTVAGHKSNGYY
>Eruca vesicaria YAB2b
MSIDLSSERVCYVHCNFCTTILAVSVPYASLFTLVTVRCGHCTNLLSLNIGVSLHQSSPAPPIHQDLQQPKQHMTSSATRKEW
GSSSRSSNHFSTTLSENVDREAPRMPPIRPPEKRQRVPSAYNRFIKEEIQRIKAGNPAISHREAFSTAAKNWAHFPHIHFGLK
LDGNKKGKQLDQTVAGHKSNGYY
>Eruca vesicaria YAB2c
MSLDLSSDRVCYVHCNFCTTILAVSVPYASLFTPVTVRCGHCTNLLSLNLGVSLHQTSPPPVHQDPQQHKQHITSSITRREYG
SSSRSSNHYSATLSENVNREAPRMPPIRPPEKRQRVPSAYNRFIKEEIQRIKAGNPEISHREAFSTAAKNWAHFPHIHFGLKL
DANKKGKQLEKTVAGQKSNGYY
>Eruca vesicaria YAB2d
MSLDLSSDRVCYVHCNFCTTILAVSVPYASLFTPVTVRCGHCTNLLSLNLGVSLHQTSPTPVHQDPQHKQHITSSITRREYGS
SSRSSNHFSTTLSENVNREAPRMPPIRPPEKRQRVPSAYNRFIKEEIQRIKAGNPEISHREAFSTAAKNWAHFPHIHFGLKLD
GNKKGKQLEKTVAGQKSNGYY
>Eruca vesicaria YAB2e
MSIDLSSERVCYVHCNFCTTILAVSVPYASLFTLVTVRCGHCTNLLSLNIGVSLHQSSPLPIHQDLQNKQHITSSVTRKEHGS

SSRSYNHFSTTLSENVEREAPRMPHIRPPEKRQRVPSAYNRFIKEEIQRIKAGNPEISHREAFSTAAKNWAHFPHIHFGLKLD
GNKKGKQIDQTVAGQKSNGYY
>Eruca vesicaria YAB2f
MSIDLSSERVCYVHCNFCTTILA
VSVPYASLFTLVTVRCGHCTNLLSLNIGVSLHQSSPLPIHQDLQQNKQHITSSVTRKEHGSSSKSYNHFSTTLSENVEREAPR
MPPIRPPEKRQRVPSAYNRFIKEEIQRIKAGNPEISHREAFSTAAKNWAHFPHIHFGLKLDGNKKGKQIDQTVAGQKSNGYY
>Eruca vesicaria YAB3a
MSSMSMSSSSAPAYPPDHISSSDHLCYVHCSFCDTVLAVSVPPSSLFKTVTVRCGHCSNLLSVTVNMRALFLPSVSNIGHSFL
PSPPPPNLLEEMRNGGQNINMNMMMSHQEVAHHSNESLVMATRNGRVDLQEMPRPPPANRPPEKRQRVPSAYNRFIKEEIQRI
KASNPDISHREAFSAAAKNWAHFPHIHFGLMPDHPPTKKANMRQQEGEEVVMGREGFYGSAANVGVTHN
>Eruca vesicaria YAB3b
MEFSETTHALAAPEKRQRVPSAYNRFIKEEIQRIKASNPDISHREAFSAAAKNWAHFPHIHFGLMPDHPPTKKANMRQQEGEE
VVMGREGFYGSAANVGVTHN
>Eruca vesicaria YAB5
MATTTTAAEQLCYVPCNFCNIVLAVSVPCSSLFDIVTVRCGHCTNLWSVNMAAALQSLSRPNFQATPYAMPEYGSSSRGHTKI
SSRISARTITEQRVVNRPPEKRQRVPSAYNQFIKEEIQRIKANNPDISHREAFSTAAKNWAHFPHIHFGLMVESNKQAKRA
>Eruca vesicaria INOa
MTKMPNMTTLNQLFDLPGQICHVQCGFCTTILLVSVPFTSLSMVVTVRCGHCTSLLSVNLMKASFIPIHLLNSLSHLDETGKE
EVEATTDGVQEEEDEEEEAWKVNQEKENSPTILVTSSDNEDEESRVYQVVNKPPEKRQRAPSAYNCFIKEEIRRLKAQNPSMA
HKEAFSLAAKNWAHFPPMQNKRAASDQCYCEEDNNALLSCNALEDHDVSNNGFRERKAQRHSIWGKSPFE
>Eruca vesicaria INOb
MTKMPNMTTLNQLFDLPGQICHVQCGFCNTILLVSVPFTSLSMVVTVRCGHCTSLLSVNLMKASFIPLHLLTSLSHLDEVPAG
KEEAAATTDGVEEEACKVNQEKENSPTTLVTSSDNEDDDRDVSRVYQVVNKPPEKRQRAPSAYNCFIKEEIRRLKAQNPSMAH
KEAFSLAAKNWANFPPVQNKRAASDQCFYEDENTALLSCNALQDHEESNNGFRERKAQRHSIWGKTPFE
>Eruca vesicaria CRC
MNLEEKPTMPSRASPQAEHLYYVRCSICNTILAVGIPMKRMLETVTVKCGHCGNLSFLTTSPPLQGHVSLTLQMQSFGGSEYK
KGSSSSSSSSTSSDQPPSPRPPFVVKPPEKKQRLPSAYNRFMRDEIQRIKSVNPEIPHREAFSAAAKNWAKYIPNSPTSITSG
GSNINGFGFGEKK
>Euclidium syriacum FIL
MSMSSISSPSSAVFSPDHLSPSEHLCYVQCNFCHTMLAVSVPYTSLFKTVTVRCGCCTNLLSVNMRSNVLPASNQLQLQLGPH
SYFNTQNILEELRDTPANMNMMMMMNQHPNMNDMSSFMDLHHQQHEIPKAPPVNRPPEKRQRVPSAYNRFIKEEIQRIKAGNP
DISHREAFSAAAKNWAHFPHIHFGLIPENQPVKKTNMPQQEGEDNNNMVMKEGFYAPAANVGVTPY
>Euclidium syriacum YAB2
MSMDLSSERVCYVHCNFCTTILAVSVPYASLFTLVTVRCGHCTNLLSLNIGVSLHQTSPPPIHQDLIQPHKQHITSSVTRKDC
GSSSRSSNSFSTTLSENVDREAPRLPPIRPPEKRQRVPSAYNRFIKEEIQRIKASNPEISHREAFSTAAKNWAHFPHIHFGLK
LDGNKKGKQLDHTVAGQKSNGYY
>Euclidium syriacum YAB3
MSSMSMSSSSAPAYPPDHISSSSSDQLCYVHCSFCDTVLAVSVPPSSVFKTVTVRCGHCSNLLSVTVNMRALLLPASVSNLGH
SFLPSPPPPPPPPPPPLLEEMRVGGQNINMNMMMSHHHHQAAAASAHQYHHATNEALVMAARTGRVDLQEMPRPPPANRPPEK
RQRVPSAYNRFIKEEIQRIKAGNPDISHREAFSAAAKNWAHFPHIHFGLMPDHPPTKKANVRQQEGEDVMMGRSEGFYGSAAN
LGVTHN
>Euclidium syriacum YAB5
MANSAIATEQLCYIPCNFCNIVLAVSVPCSSLFDIVTVRCGHCTNLWSVNMAAALQSLSRPNFQATNYAMPEYGSSSRGHSKI
PSRTRTITEQRVVNRPPEKRQRVPSAYNQFIKEEIQRIKANNPDISHREAFSTAAKNWAHFPHIHFGLMLDSNKQAKLA
>Euclidium syriacum INO

MTRIPNMTTLNHLFDLPGQICHVQCGFCTTILLVSVPFTSLSMVVTVRCGHCTSLLSVNLMKASFIPLHLLASLSHLDETGKE
EVAATDGVEDEARKTNQEKDSPTTLVTSSDNEDEDNDLSRVYQVVNKPPEKRQRAPSAYNCFIKEEIRRLKAQNPSMAHKEAF
SLAAKNWAHFPPVLHNKRAASEQCFCEEDNNAVLPCNALEDHEESNNGFRERKAQRHSIWGKSPFE
>Euclidium syriacum CRC
MNLEDKPTMASSRASPQAEHLYYVRCSICNTILAVGIPLKRMLDTVTVKCGHCGNLSFLTTSPPLQGHVSLTLQMQSFGGSEY
KKGSSSSSSSSTSSDQPPSPTPPFVVKPPEKKQRLPSAYNRFMRDEIQRIKSANPEIPHREAFSAAAKNWAKYIPNSPTSIAS
GGNTIHGLGFGEKK
>Iberis amara FILa
MSMSSISSPSSAVFSPDHLSPSEHLCYVQCNFCDTLLAVSVPYTSLFKTVTVRCGCCTNLLSVNMRTLALPASNQLQLQLGPN
SYFNPQNILEELRDAPSNMNMMMMNQHPNMNNDIPPFMDLHQQHEIPKAPPVNRPPEKRQRVPSAYNRFIKEEIQRIKASNPD
ISHREAFSAAAKNWAHFPHIHFGLVPDNQPVKKPNMPQQDGESNMVKEGYYAPVANVGVTPY
>Iberis amara FILb
MSMSSPSSGVFSPEHLSPSEHLCYVRCNFCETILAVNVPYTSLFKTVTVRCGCCTNLLSVNMRSLVLPATNQLQLGPNCYFNP
QNILEELRDAPTNMNMMMMNQHPNMNDIPSSFMDHHQQHEIPKAPPINRPPEKRQRVPSAYNRFIKEEIQRIKAGNPDISHRE
AFSAAAKNWAHFPHIHFGLVPPDNQPVKKTNMPQLEGEGNMVMKEGFYAPPVVTPY
>Iberis amara YAB2a
MSIDLSSERVCYVHCNFCTTILAVGVPYASLFTFVTVRCGHCTNLLSLNIGVSLHQTSPPPTHQDVLQQHKQHITSTSITTKK
DCGSSSSRTTNHFSTTLSENVDREAPRMPPIRPPEKRQRVPSAYNRFIKEEIQRIKASNPEISHREAFSTAAKNWAHFPHIHF
GLKLDGNKKGKQLDQTLAGQKSNGFY
>Iberis amara YAB2b
MSIDLSSERVCYVHCSFCTTILAVSVPYASLFTLVTVRCGHCTNLLSLNIGVSLHQTSPPSTPQDLQPHKQLMTSSVTRKECG
SSSSKSTNSLSTTLSQNIDREAPKLPPIRPPEKRQRVPSAYNRFIKEEIQRIKACNPEISHREAFSTAAKNWAHFPHIHFGLK
LDGNKKGKQLDQTVASGQNSNGYY
>Iberis amara YAB2c
MLVSVPYASLFTLVTVRCGHCTNLLSLNIGVSLHQTSPPNSIHQDLQPHKQLMTSSVTRKECGSSSSKSTNSLSTTLSQNIDQ
EAPKLPPIRPPEKRQRVPSAYNRFIKEEIQRIKACNPEISHREAFSAAAKNWAHFPHIHFGLKLDGNKKGKQLDQTVASGQKS
NGYY
>Iberis amara YAB3a
MSSMSMWSSSSLAYPPDHISSSDQLCYVHCRFCDTVLAVSVPPSSLLKTVTVRCGHCSNLLSVTVNMRSLLLPSAHSFLLPPN
LLQEELQNKNMSHHTAANGSVDLPPPIRPQEKRQRVPSAYNRFIKEEIQRIKAGNPLITHREAFSAAAKNWAHFPRIYYGLMA
DNHPTKKAHVHQEDGEDVMIGREGFYGSVNNIGLTHN
>Iberis amara YAB3b
MSNMSMSSSSEPTSPPDHISSSDQLCYVHCSFCNTILAVSVPPSSLFKTVTVRCGHCSNLLSVTANVRALLPPSSVGHPFLPP
PPNLLEEIRKGGQNINMNMNMMMSHQATADEPFVVASRTGRVDMPRPPPPNKPPEKRQRVPSAYNRFIKEEIQRIKAGNPDIS
HREAFSAAAKNWAHFPHIHFGLVSDHPPTKKANVRQQKGEDVMMGRDGFYGSAANVGVTHN
>Iberis amara YAB5
MANSEQLCYIPCNFCNIVLAVSVPCSSLFDIVTVRCGHCTNLWSVNMAAALQSLSRPNFQGTNYGMSSEYGSSSRGHTKIPSR
NSSRTINEPRVVNRPPEKRQRVPSAYNQFIKEEIQRIKANNPDITHREAFSTAAKNWAHFPHIHFGLMLESNKQAKLA
>Iberis amara INOa
MTRMHNMTTLNHLFDLPGQICHVQCGFCTTILLVSVPFTSLSMVVTVRCGHCTSLLSVNLMKASFIPLHLLASLSHLDHEVGK
EEVVTTTDGVEEEAWKLNQEKDSPTTLVSSSDNEDEDKDVSRVYQVVNKPPEKRQRAPSAYNCFIKEEIRRLKAQNPSMAHKE
AFSLAAKNWAHFPPVHNKRAASDQCFCDDDNNVVLCNALQDHEESNNGFRERKAQRHSIWGKSPFE
>Iberis amara INOb
MTRMHNMTTLNHLFDLPGQICHVQCGFCTTILLVSVPFTSLSMVVTVRCGHCTSLLSVNLMKASFIPLHLLASLSHLDEVGKE
EVVTTTTDGVEEEAWKLNQEKGSPTTLVSSSDNEDEDKDVSRVYQVVNKPPEKRQRAPSAYNCFIKEEIRRLKAQNPSMAHKE

AFSLAAKNWAHFPPVHNKRAASDQCFCDDDNNVVLCNALQDHEESNNGFRERKAQRHSIWGKSPFE

>Iberis amara CRC

MNLEEKPTMGGSRASPQGEHLYYVRCSICNTILAVGIPLKRMLDTVTVKCGHCGNLSFLTTSPPLQGHVSLTLQMQSFGGNEF
KKGSSSSSSSSTSSDQPPSPRPPFVVKPPEKKQRLPSAYNRFMRDEIQRIKSANPEVPHREAFSAAAKNWAKYIPNSPTSITS
GGNNINGLGFGEK

>Isatis tinctoria FILa

MSMSSMSSPSSAVFSPEHLSPSDHLCYVQCNFCETILAVNVPYTSLFKTVTVRCGCCTNLLTVFVQDRNLLSVNMRSAVLPAS
NQLQLQLGPHSYFNPQNILEELRDAPSNMNMMMMNQHPNMNDMPSFMDLHQQHEIPKAPPVNRPPEKRQRVPSAYNRFIKEEI
QRIKAGNPDISHREAFSAAAKNWAHFPHIHFGLVPDNQPVKKTNMPQQEGEDNMGMKEGFYAPAANVGVAPY

>Isatis tinctoria FILb

MSMSSMSSPSSAVFSPEHLSPSDHLCYVQCNFCETILAVNVPYTSLFKTVTVRCGCCTNLLTVFVQDRNLLSVNMRSAVLPAS
NQLQLQLGPHSYFNPQNILEELRDAPSNMNMMMMNQHPNMNDMPSFMDLHQQHEIPKAPPVNRPPEKRQRVPSAYNRFIKEEI
QRIKAGNPDISHREAFSAAAKNWAHFPHIHFGLVPDNQPVKKTNMPQQEGEDNMGMKEGFYAPAANVGVAPY

>Isatis tinctoria FILc

MSMSSMSSPSSAVFSPEHLSPSDHLCYVQCNFCETILAVNVPYTSLFKTVTVRCGCCTNLLTVFVQDRNLLSVNMRSAVLPAS
NQLQLQLGPHSYFNPQNILEELRDAPSNMNMMMMNQHPNMNDMPSFMDLHQQHEIPKAPPVNRPPEKRQRVPSAYNRFIKEEI
QRIKAGNPDISHREAFSAAAKNWAHFPHIHFGLVPDNQPVKKTNMPQQDGDDNMGMKEGFYAPAANVGVAPY

>Isatis tinctoria FILd

MSMSSMSSPSSAVFSPEHLSPSDHLCYVQCNFCETILAVNVPYTSLFKTVTVRCGCCTNLLTVFVQDRNLLSVNMRSAVLPAS
NQLQLQLGPHSYFNPQNILEELRDAPSNMNMMMMNQHPNMNDMPSFMDLHQQHEIPKAPPVNRPPEKRQRVPSAYNRFIKEEI
QRIKAGNPDISHREAFSAAAKNWAHFPHIHFGLVPDNQPVKKTNMPQPDGEDNMGMKEGFYAPAANVGVAPY

>Isatis tinctoria YAB2a

MSIDISSERVCYVHCNFCTTILALYSLTSYYILXRCGHCTNLLSLNIGVSLHQTSPPTIHQDLQQHKQHITSSVTRKECGSSS
RSSNHFATTLSENVDREAPRMPPIRPPEKRQRVPSAYNRFIKEEIQRIKAGNPEISHREAFSTAAKNWAHFPHIHFGLKLDGN
KKGKQLDQTVAGQKSNGYY

>Isatis tinctoria YAB2b

VSVPYASLFTLVTVRCGHCTNLLSLNIGVSLHQTSPPTIHQDLQQHKQHITSSVTRKECGSSSRSSNHFATTLSENVDREAPR
MPPIRPPEKRQRVPSAYNRFIKEEIQRIKAGNPEISHREAFSTAAKNWAHFPHIHFGLKLDGNKKGKQLDQTVAGQKSNGYY

>Isatis tinctoria YAB3a

MEFSEITHALAAPEKRQRVPSAYNRFIKEEIQRIKAGNPDISHREAFSAAAKNWAHFPHIHFGLMPDHPPTKKANVRPQEGED
VMMGREGFYGSAANVGVTHN

>Isatis tinctoria YAB3b

MRNGGQNINMNMMSHQAAAHHSNESLVMSTRSGRVDLQEMPRPPPANRPPEKRQRVPSAYNRFIKEEIQRIKAGNPDISHREA
FSAAAKNWAHFPHIHFGLMPDHPPTKKANVRPQEGEDVMMGREGFYGSAANVGVTHN

>Isatis tinctoria YAB3c

WAHFPHIHFGLMPDHPPTKKANVRQQEGEDVMMGREGFYGSAANVGVTHN

>Isatis tinctoria YAB5

MANSATATEQLCYIPCNFCNIVLAVSVPCSSLFDIVTVRCGHCTNLWSVNMAAALQSLSRPNFQATNYAMPEYGSSSRGHTKI
PSRISARTITEQRVVNRPPEKRQRVPSAYNQFIKEEIQRIKANNPDISHREAFSTAAKNWAHFPHIHFGLMLESNKQAKLA

>Isatis tinctoria INOa

MTKMPNMTTLNQLFDLPGQICHVQCGFCTTILLVSVPFTSLSMVVTVRCGHCTSLLSVNLMKASFIPLHLLTSLSHLDEAGKE
EVTAATTDVVEEEAWKVNQEKENSPTTLVTSSDNEDEDKDMSRVYQVVNKPPEKRQRAPSAYNCFIKEEIRRLKAQNPSMAHK
EAFSLAAKNWAHFPPAQNKRAASDQYFCEDDNSALLSCNALENHEEINNGFRERKAQRHSMWGKSPFE

>Isatis tinctoria INOb

MTKMPNMTTLNQLFDLPGQICHVQCGFCTTILLVSVPFTSLSMVVTVRCGHCTSLLSVNLMKASFIPLHLLTSLSHLDEAGKE

EVTAATTDVVEEEAWKVNQEKENSPTTLVTSSDNEDEDKDMSRVYQVVNKPPEKRQRAPSAYNCFIKEEIRRLKAQNPSMAHK
EAFSLAAKNWAHFPPAQNKRAASDQCFCDDDNSALLSCNALENHEEMINNGFRERKAQRHSMWGKSPFE

>Isatis tinctoria INOc

MTKMPNMTTLNQLFDLPGQICHVQCGFCTTILLVSVPFTSLSMVVTVRCGHCTSLLSVNLMKASFIPLHLLTSLSHLDEAGKE
EVTAATTDVVEEEAWKVNQEKENSPTTLVTSSDNEDEDKDMSRVYQVVNKPPEKRQRAPSAYNCFIKEEIRRLKAQNPSMAHK
EAFSLAAKNWAHFPPAQNKRAASDQCFCEDDNSALLSCNALENHEKINNGFRERKAQRHSMWGKSPFE

>Isatis tinctoria CRCa

MNLEEKTTMASRASPQAEHLYYVRCSICNTILAVGIPLKRMLDTVTVKCGHCGHLSFLTTSPPLQGHVSLSLQMQSFGGSEYK
KGSSSSSSSSTSSDQPPSPRPPFVVKPPEKKQRLPSAYNRFMRDEIQRIKSANPEIPHREAFSAAAKNWAKYIPNSPTSITSG
ANNISGFVFGEKK

>Isatis tinctoria CRCb

MNLEEKTTMASRASPQAEHLYYVRCSICNTILAVGIPLKRMLDTVTVKCGHCGHLSFLTTSPPLQGHVSLSLQMQSFGGSEYK
KGSSSSSSSSTSSDQPPSPRPPFVVKPPEKKQRLPSAYNRFMRDEIQRIKSANPEIPHREAFSAAAKNWAKYIPNSPTSITSG
ANNINGFVFGEKK

>Leavenworthia alabamica FILa

MSMSSSSSDVFSPDNLSPSDHLCYVQCNFCGTLLAVSVPYTSLYKTVTVRCGCCTNLLSVNMRTHVLPASSQLQLQLGPHSYY
NTQNILEELRDAPSNMNMMMMNQHPNMNDIPSFIDLNQQREIPKAPPVNRPPEKRQRVPSAYNRFIKEEIQRIKAGNPDISHR
EAFSAAAKNWAHFPHIHLGLVPDNQPVKKTNMPQQDGDDNMVMKEGFYAPLANVGVTPY

>Leavenworthia alabamica FILb

MSMSMSSPSSDVFSPDHLSPSEHLCYVQCNFCGTLLAVSVPYTSLFKTVTVRCGCCTNLLSVNMRTHVLPASNQLQLQLGPHP
CYNPQNILEELRDAPSNMNMMMMNQHPNMNDIPSFMDLHQQHDIPKAPPVNRPPEKRQRVPSAYNRFIKEEIQRIKAGNPDIS
HREAFSAAAKNWAHFPHIHFGLVPDNQPVKKTNMPQQDGEDNNNMVLKEGFYAPTAGVTPY

>Leavenworthia alabamica YAB2

MSIDLSSERVCYVHCSFCTTILAVSVPYASLFTLVTVRCGHCTNLLSLNIGVSLHQTSAAPVHQDLQPHKQHITSSVTRKDCG
SSSRSTSNLSENIDREAPRMPPIRPPEKRQRVPSAYNRFIKEEIQRIKACNPEISHREAFSTAAKNWAHFPHIHFGLKLDGNK
KGKQLDQTVAGQKSNGYY

>Leavenworthia alabamica YAB5

MTNSGTSMEQLCYIPCNFCNIVLAVSVPCSSLFDIVTVRCGHCTNLWSVNMAGALQSLSRPNFQATNYAIPEYGGSSSRGHTK
IPSRISTRTVTEQRVVNRPPEKRQRVPSAYNQFIKEEIQRIKANNPDISHREAFSTAAKNWAHFPHIHFGLMLESNKQAKIA

>Leavenworthia alabamica INOa

VSVPFTSLSMVVTVRCGHCTSLLSVNLMKASFIPLHILASLSHLDEAGENEVAATDGVEEEAWNVDQEKVDTQTTLVSSSDNE
DEDVSRVYQVVNKPPEKRQRAPSAYNCFIKEEIRRLKAQNPSMVHKEAFSLAAKNWAHFPSVHNKRAASGQCFCEEGNNEELH
CNALQDHEESNNESRERKAQRHSIYGKSPFE

>Leavenworthia alabamica INOb

MTIMPNMTTLNHLFDLPGQICHVQCGFCTTILLVSVPFTSLSMVVTVRCGHCTSLLSVNLMKASFIPLHLLASLSHLDEAGKE
EVAATDGVEEEAWKVNQEKESPTTLVSSSDNEDEDVSRRVYQVVNKPPEKRQRAPSAYNRFIKEEIRRLKAQNPSMAHKEAFS
LAAKNWAHFPLHNKRAASGQCFCEEGNNEVLQCNSLEDHEESNNGFRERKAQRHSIWGKSPLFE

>Leavenworthia alabamica CRC

MNLEEKPTNNMASRASPAQAEHLYYVRCSICNTILAVGIPLKRMLDTVTVKCGHCGNLSFLTTSPPLQGHVNLTLQMQSFGGN
EYKKGSSSSSSSSTSSDQPSSPKPPFVVKPPEKKQRLPSAYNRFMRDEIQRIKSANPEIPHREAFSAAAKNWAKYIPNSPTSL
TSGGNNINGLGFGEKK

>Lepidium sativum FILa

MSMSSPSSAVFSPDHLSPNEHLCYVRCNFCQTILAVSVPYTSLFKTVTVRCGCCTNLLSVNMRSHVLPPSNQLQLQLGPHSYF
DPQNILEELRDAPSNMNMMMINQHPNMNDLPSFMDLHQQHEIPKAPPVNRPPEKRQRVPSAYNRFIKEEIQRIKAGNPDISHR
EAFSAAAKNWAHFPHIHFGLVPDNQPVKKTNMPQQEGDDNNMVMKDGFYAPSPNVGVTPY

>Lepidium sativum FILb
MSMSSPSSAVFSPDHLSPNEHLCYVRCNFCQTSLAVSVPYTSLFKTVTVRCGCCTNLLSVNMRSHVLPASNQLQLQLGLHSYF
DPQNILGELRDSPSNMNMMMMNQHPNMNDLPSFMDLHQQHEIPKAPPVNRPPEKRQRVPSAYNRFIKEEIQRIKAGNPDISHR
EAFSAAAKNWAHFPHIHSGLVPDIQPVKKTNMPQQEGDDNNMVMKDGFYAPAANVGVTPY
>Lepidium sativum YAB2a
MSVELSSERVCYVHCNFCTTILAVSVPYASLFTLVTVRCGHCTNLLSLNIGVSLHQSSPPPIHQDLHQPHKQHITSSVTRKDG
ASSSRSTNNFSESIDREAPRMPPIRPPEKRQRVPSAYNRFIKEEIQRIKACNPEISHREAFSTAAKNWAHFPHIHFGLKLDGN
KKGKQLDQSAASQKSNGYY
>Lepidium sativum YAB2b
MSVELSSERVCYVHCNFCTTILAVSVPYASLFTLVTVRCGHCTNLLSLNIGVSLHQSSPPPVHQDLQPHKQHITSSVTRKDCA
SSSRSNNNFSESTDREAPRMPPIRPPEKRQRVPSAYNRFIKEEIQRIKACNPEISHREAFSTAAKNWAHFPHIHFGLKLDGNK
KGKQLDQSAASQKSNGYY
>Lepidium sativum YAB3a
MSSLSMSSSSETAAYSPDHISSSEQLCYVHCSFCDTVLAVSVPPSSLFKTVTVRCGHCSNLLSVTVSMRALLLPSISNLPHSL
PPPNHLEEMNMMMSQHSAAHHTDESLVMATRHGRVDHLQELPRPPPANRPAPEKRQRVPSAYNRFIKEEIQRIKASNPEISHR
EAFSAAAKNWAHFPHIHFGLMGGADHPHPPTKKNPNVRHHQADHPFTNN
>Lepidium sativum YAB3b
MSSASGEEVLDIYKSMSSLSMSSSSAPAAYSPDHISSSEQLCYVHCSFCDTVLAVSVPPSSLFKTVTVRCGHCSNLLSVTVSM
RALFLPSVSNLPHPLPPPNHLEEMRSGGQNINMNMMMSQHGAAHHPDESLVMATRHGRVDHLQEMPRPPPPNRPPEKRQRVPS
AYNRFIKEEIQRIKASNPEISHREAFSAAAKNWAHFPHIHFGLMGGAHHPHPPTKKKAIM
>Lepidium sativum INOa
MTRMANMTTLNNLFDIPGQICHVQCGFCTTILLVSVPFTSLSMVVTVRCGHCTSLLSVNLMKASFLPLHLLASVSHIDETGKE
EVTATDGVEKEAWKVNHQEKENSPTTLVTSSDNEDEDVSRVYQVVNKPPEKRQRAPSAYNCFIKEEIRRLKAQNPSMAHKDAF
SLAAKNWAHFPPVQNKRATSDQCFCQEDNNAVQPCNALEDHEESYIGFRERKAQRQFIWGKSPFE
>Lepidium sativum INOb
MTRMANMTTLNNLFDIPGQICHVQCGFCTTILLVSVPFTSLSMVVTVRCGHCTSLLSVNLMKASFIPLHLLASLSHLDEAGKE
EVTATDGVEEEAWKLNQEKENSPTTLVTSSDNEDEDLSRVYQVVNKPPEKRQRAPSAYNCFIKEEIRRLKAQNPSMAHKEAFS
LAAKNWAHFPPVQNKRATSDQCFCQEDNNAVQPCNALEDHEESYNGFRERKAQRQSNWGKSPFE
>Lepidium sativum CRCa
MNLDQEKPSMTSRASPQAEHLYYVRCSICNTILAVGIPMKRMLDTVTVKCGHCGNLSFLTTSPPLQGHVSLTLQMQSFGGSEY
KKGSSSSSSSSTSSDQTPSPTPPFVVKPPEKKQRLPSAYNRFMRDEIQRIKTANPEIPHREAFSAAAKNWAKYIPNSPTSLTS
GGNHMMNGLGFGEKK
>Lepidium sativum CRCb
MMNLDQEKPTMTSRASPQAEHLYYVRCSICNTILAVGIPMKRMLDTVTVKCGHCGNLSFLTTSPPLQGHVSLTLQMQSFGGSE
YKKGSSSSSSFSTSSDQPPSPTPPFVVKPPEKKQRLPSAYNRFMRDEIQRIKTANPEIPHREAFSAAAKNWAKYIPNSPTSLT
SGGSHMNMMNGLGLGEKK
>Lunaria annua FILa
MSMSSPSSAVFSPDHLSPSDHLCYVQCNFCETILAVSVPYTSLFKTVTVRCGCCTNLLSVNMRSLVLPASNQLQLQLGPHSYF
NSQNIMEELRDAPSNMNLMMMNQHPNMNDVPSFMDLHQQHEIPKAPPVNRPPEKRQRVPSAYNRFIKEEIQRIKASNPDISHR
EAFSAAAKNWAHFPHIHFGLVPDNQPVKKTNMPHQEGEDNMVMKEGFYASAGNVGVTPH
>Lunaria annua FILb
MSMSSPSSAVFSPDHLSPSEHLCYVQCNFCETILAVSVPYTSLFKTVTVRCGCCTNLLSVNMRSLVHPASNQLQLQLGPNSYF
NTQNILEELRDAPSNMNMMMMNQHPNMNDIPSFMDLHQQHEIPKAPPVNRPPEKRQRVPSAYNRFIKEEIQRIKAGNPDISHR
EAFSAAAKNWAHFPHIHFGLVPDNQPVKKTNMPQQEGDDNMVMKEGFYAPAANVGVTPY
>Lunaria annua FILc

MSMSSPSSAVFSPDHLSPSEHLCYVQCNFCETILVVSVPYTSLFKTVTVRCGCCTNLLSVNMRSLVLPASNQLQLQLGPNSYF
NTQNILEELRDAPSNMNMMMMNQHPNMNDIPSFMDLHQQHEIPKAPPVNRPPEKRQRVPSAYNRSIKEEIQRIKAGNPDISHR
EAFSAAAKNWAHFPHIHFGLVPDNQPVKKTNMPQQEGDDNMVMKEGFYAPAANVGVTPY
>Lunaria annua YAB2a
MSLDLSSERVCYVHCSFCTTILAVSVPYASLFTLVTVRCGHCTNLLSLNIGVSLHQTSPPPIHQDLQPHKPNITSSVTRNECG
SSSRSTNNLSPTLSENIDRESPRMPPIRPPEKRQRVPSAYNRFIKEEIQRIKACNPEITHREAFSTAAKNWAHFPHIHFGLKL
DGNKKGKQLDQTVAGQKSNGYY
>Lunaria annua YAB2b
MSIDLSSERVCYVHCNFCTTILAVSVPYASLFTLVTVRCGHCTNLLSLNIGVSLHQTSQAPPIHQDLQQHKQNITSSVTRKEC
GSSSRSTNHFSTTLSENVDREAPRLPPIRPPEKRQRVPSAYNRFIKEEIQRIKAGNPEISHREAFSTAAKNWAHFPHIHFGLK
LDGNKKGKQLDQTVAGQKSNGYY
>Lunaria annua YAB3a
MSSMPMSSSSAPAYQPDHISSSDQLCYVHCSFCDTVLAVSVPPTSLFKTVTVRCGHCSNLLSVTVNMRALLLPSVSNLGRSFL
PPPPNLLEEMRNGGQNINMNMNMNMNMNMMMNYETANESLVIQEMPRPVPPANKPPEKRQRVPSAYNRFIKEEIQRIKAGNPD
ISHREAFSAAAKNWAHFPHIHFGLMPDHPPTKKAKEGDQDVMMGREGLYGSAANVGVTP
>Lunaria annua YAB3b
MSSMSMSSSSAPAYQPDHISSSDQLCYVHCSFCDTVLAVSVPPTSLFKTVTVRCGHCSNLLSVTVNTRALLLPSVSSLGHSFL
PPPPPPHPNLLEEMRNMNMNMNMMMSHQTTTNHHSNEPLVMASRSGRVDLQEMPRPPPANRPAPEKRQRVPSAYNRFIKEEIQ
RLKAGNPDISHREAFSAAAKNWAHFPHMHFGLMPDHPPTKKANVRQQEGDDVMMLGRAQGFYGSPANLGVTHN
>Lunaria annua YAB5
MANSAMATEQLCYIPCNFCNIVLAVSVPCSSLFDIVTVRCGHCTNLWSVNMAAALQSLSRPNFQATNYALPEYGSSSRGHTKI
PSRISSRTVTEQRVVNRPPEKRQRVPSAYNQFIKEEIQRIKANNPDISHREAFSTAAKNWAHFPHIHFGLMLESNKQAKLA
>Lunaria annua INOa
MTTMPNMTTLNHLFDLPGQICHVQCGFCTTVLLVSVPFTSLSMVVTVRCGHCTSLLSVNLMKASFIPLHLLASLSHLDEAGKE
EVAAITDGVEEEAWKLNEEKEKENSPTTLVSSSDNEDEDKDVSRVYQVVNKPPEKRQRAPSAYNCFIKEEIRRLKSQNPSMAH
KEAFSLAAKNWAHFPPVQNKRAASDQCLCEEDNNTVLTCNALENHEESNNGFRERKAQRHSIWGKSPFE
>Lunaria annua INOb
MTTLNHLLDLPGQICHVQCGFCTTILLVSVPFTSLSMVVTVRCGHCTSLLSVNLMKASFIPLHLLASLSHLDEAGKEEVAVTT
DGVEEEAWKLNQDKDSPTTLVSSSDNEDEDKDVSHVYQVVNKPPEKRQRAPSAYNCFIKEEIRRLKAQNPSMAHKEAFSLAAK
NWAHFPPVHNKRAASDQCFWEEDNNAVLQCNALEKPIQLIKELELNMYIFSIHVLIYCLNVCGMQDHEQNNNGFRERKAQRHS
MWGKSPFE
>Lunaria annua CRCa
MNLEEKPTMASRASPQAEHLYYVRCSICNTILAVGIPLKRMIDTVTVKCGHCGNLSFLTTSPPLQGRVSLTLQMQSFGGSEYK
KGNSSSSSSSFSSDQPPSPSPPFVVKPPEKKQRLPSAYNRFMREEIQRIKSANPEIPHREAFSTAAKNWAKYIPNSPTS
>Lunaria annua CRCb
MNLEDKPTMASRASPQTEHLYYVRCSICNTILAVGIPLKRMLDTVTVKCGHCGNLSFLTTSPPLQGHVSLTLEMQSFCGNEYK
KGSSSSSSSSTSSDQPPSPTPPFVVKPPEKKQRLPSAYNRFMRDEIQRIKSANPEIPHREAFSAAAKNWAKYIPNSPTSITSG
TNNI
>Malcolmia maritima FIL
MSMSMSSPSSAVFSPDHLSPSDHLCYVQCNFCHTILAVSVPYTSLFKNVTVRCGCCTNLLSVTMKSHVFPASNQLQLQLGPHS
YFNTQNILEELRDAPSNMNMMMMNQHPNMNDIPSFMDLHQQHEIPKAPPVNRPPEKRQRVPSAYNRFIKEEIQRIKAGNPDIS
HREAFSAAAKNWAHFPHIHFGLVPDNQPVKKTNMPQQEGEDNMMMKEGFYAPAANVGVTPY
>Malcolmia maritima YAB2
MSVDLSSERVCYVHCNFCTTILAVSVPYASLFTLVTVRCGHCTNLLSLNIGVSLHQTSPPPIHQDLQPHRQHMTSSVTRKDCA
SSSRSTNNLSENMDREAPRMPPIRPPEKRQRVPSAYNRFIKEEIQRIKACNPEISHREAFSTAAKNWAHFPHIHFGLKLDGNK

KGKQLDQSAAGQKSNGYY
>Malcolmia maritima YAB3
MSSMSMSSSSAPAYPPDHISSTDQLCYVHCSFCDTVLAVSVPPSSLFKTVTVRCGHCSNLLSVTVSMRALILPSVSNLGHSFL
PPPPPPPPNLLEEMRSGGQNINMNMMMSHPAAAHHHPNESLVMATRNGRVDHLQEMPRPPPANRPPEKRQRVPSAYNRFIKEE
IQRIKAGNPDISHREAFSAAAKNWAHFPHIHFGLMADHPPTKKANVRQQEGEDVMIGREGFYGSAANVGVTHN
>Malcolmia maritima YAB5
MANSAMATEQLCYIPCNFCNIVLAVSVPCSSLFDIVTVRCGHCTNLWSVNMAAALQSLSRPNFQATNYAIPEYGSSSRGHTKI
PSRISTRSTVSEQRIVNRPPEKRQRVPSAYNQFIKEEIQRIKANNPDISHREAFSTAAKNWAHFPHIHFGLMLENNKQAKLA
>Malcolmia maritima INO
MTRMPNMTSLNHLFDLPGQICHVQCGFCTTILLVSVPFTSLSMVVTVRCGHCTSLLSVNLMKASFIPLHLLASLSHLDETGKE
EVTATDGVEEEACKVNQEKENSPTTLVTSSDNEDEDVPRVYQVVNKPPEKRQRAPSAYNCFIKEEIRRLKAQNPSMAHKEAFS
LAAKNWAHFPPVHNKRAASDQCFCEEDNNAVLPCNALEDHEESNNGFRERKAQRHSIWGKSPFE
>Malcolmia maritima CRC
MNLEDKPTSLTSRPSPQAEHLYYVRCSICNTILAVGIPLKRMLDTVTVKCGHCGNLSFLTTSPPLQGHVSLTLQMQSFGGSEY
KKGSSSSSSSSTSSDQPPSPTPPFVVKPPEKKQRLPSAYNRFMRDEIQRIKSANPEIPHREAFSAAAKNWAKYIPNSPTSITS
GGNNIHGLGFLEKK
>Myagrum perfoliatum FIL
MSMSSMSSPSSAVFSPEHLSPSDHLCYVQCNFCETILAVNVPYTSLFKTVTVRCGCCTNLLTVFVQDRNLLSVNMRSAVLPAS
NQLQLQLGPHSYFNPQNILEELRDAPSNMNMMMMNQHPNMNDIPSFMDLHQQHEIPKAPPVNRPPEKRQRVPSAYNRFIKEEI
QRIKAGNPDISHREAFSAAAKNWAHFPHIHFGLVPDNQPVKKTNMPQQEGEDNMGMKDGFYAPAANVGVAPY
>Myagrum perfoliatum YAB2
MSIDLSSERVCYVHCNFCTTILAVSVPYASLFTLVTVRCGHCTNLLSLNIGVSLHQSSPPTIHQDLQHKQHVTSSVTRKECGS
SSRSSNHFSTTMSENVDREAPRMPPIRPPEKRQRVPSAYNRFIKEEIQRIKAGNPEISHREAFSTAAKNWAHFPHIHFGLKLD
GNKKGKQLDQTVAGQKSNGYY
>Myagrum perfoliatum YAB3
MSSMSMSSSSAPAYPPDHISSLDQLCYVHCSFCDTVLAVSVPPSSLFKTVTVRCGHCSNLLSVTVNMRALLLPSVSNSGHSFL
PPPPPPPNLLEEMRNGGQNINMNMMSHQGAAQHSNESLVMATRNGRVDLQEMPRPPPANRPPEKRQRVPSAYNRFIKEEIQRI
KAGNPDISHREAFSAAAKNWAHFPHIHFGLMPDHPPTKKANVRQQEGEDVMMGREGFYGSAANVGVTHN
>Myagrum perfoliatum YAB5
MANSTTATEQLCYIPCNFCNIVLAVSVPCSSLFDIVTVRCGHCTNLWSVNMAAALQSLSRPNFQATKNAMPEYGSSSRGHTKI
PSRISARTITEQRVANRPPEKRQRVPSAYNQFIKEEIQRIKANNPDISHREAFSTAAKNWAHFPHIHFGLMLESNKQAKLA
>Myagrum perfoliatum INO
MTKMPNMTTLNQLFDLPGQICHVQCGFCTTILLVSVPFTSLSMVVTVRCGHCTSILSVNLMKASFIPLHLLTSLSHLDEAGKE
EVTTATNVVDEEAWKVNQEKENSPTTLVTSSDNEDEDKDVSRVYQVVNKPPEKRQRAPSAYNCFIKEEIRRLKAQNPSMAHKE
AFSLAAKNWAHFPPAQNKRAASDQCFCEDDNSALLSCNALEDHEESNKGFRERKAQRHSIWGKSPFE
>Myagrum perfoliatum CRC
MNLEEKSSMASRASPQAEHLYYVRCSICNTILAVGIPFKRMLDTVTVKCGHCGNLSFLTTSPPLQGHVSLTLQMQSFGGSEYK
KGSSSSSSSSTSSDQPTSPRPPFVVKPPEKKQRLPSAYNRFMRDEIQRIKSANPEIPHREAFSAAAKNWAKYIPNSPTSLTSG
ANNINGFVFGEKK
>Rorippa islandica FIL
MSTSMSSPSSDVFSPDHLSPSEHLCYVQCNFCGTLLAVSVPYTSLFKTVTVRCGCCTNLLSVNMRTHVLPASNQLQLQLGPHP
YYNPQNILEELREAPSNMNMMMMNQHPNMNDIPSFMDLHQQHEIPKAPPVNRPPEKRQRVPSAYNRFIKEEIQRIKAGNPDIS
HREAFSAAAKNWAHFPHIHFGLVPDNQPVKKTNMPQQDGEDSMVMKEGFYAPTANVGVTPY
>Rorippa islandica YAB2
MSIDLSSERVCYVHCSFCTTILAVSVPYASLFTLVTVRCGHCTNLLSLNIGVSLHQTSAAPVHQDLQPHKQHITSSVTRKDCG

SSSRSTCNLSENIDREAPRMPPVRPPEKRQRVPSAYNRFIKEEIQRIKACNPEISHREAFSTAAKNWAHFPHIHFGLKLDGNK
KGKQLDQTVAGQKSNGYY

>Rorippa islandica YAB3

MSSMSMSSSSAPAYPPDHISSTEQLCYVHCSFCDTVLAVSVPPSSLFKTVTVRCGHCSNLLSVTVNMRTLLLPSVSNLGHPFL
PPPPPPNVLEEMRSGGHNINMNMMGAAHHPNESLVMATRHGIVDHLQEMPRPPPANRPAPEKRQRVPSAYNRFIKEEIQRIKA
GNPDISHREAFSAAAKNWAHFPHIHFGLMADHPPSKKANVRQQEGEDVMIGREGFYGSAANVGVTHN

>Rorippa islandica YAB5

MANSGTATEQLCYIPCNFCNIVLAVSVPCSSLFDIVTVRCGHCTNLWSVNMAGALQSLSRPNFQATNYATTEYGSSSRGHTKI
PSRISTRTITEQRVVNRPPEKRQRVPSAYNQFIKEEIQRIKANNPDINHREAFSTAAKNWAHFPHIHFGLMLESNKQAKPA

>Rorippa islandica INO

MTRMPNMTTLNHLFDLPGQICHVQCGFCTTILLVSVPFTSLSMVVTVRCGHCTSLLSVNLMKASFIPLHLLASLSHLDEAGKE
EVAATDGVEEEAWKVNQEKDSPTTLVSSSDNEDEDVSRVYQVVNKPPEKRQRAPSAYNCFIKEEIRRLKAQNPSMAHKEAFSL
AAKNWAHFPPVHNKRAASGKCFCEEGNNEVLACNALEDHEESNNGFRERKAQRHSIWGKSPFEQQ

>Rorippa islandica CRC

MNLEEKPTMASRASPAQAEHLYYVRCSICNTILAVGIPLKRMLDTVTVKCGHCGNLSFLTTSPPIQGHVSLTLQMQSFGGNEY
KKGSSSSSSSSTSSDQPSSPRPPFVVKPPEKKQRLPSAYNRFMRDEIQRIKSANPEIPHREAFSAAAKNWAKYIPNSPTSLTS
GGNNLNGLGFGEKK

>Schrenkiella parvula FIL

MSMSSMSSPSSAVFSPEHLSPSEHLCYVQCNFCQTILAVSVPFTGLFKTVTVRCGCCTNLLSVNTRLVLPASNQLQLQLGPHS
YFNSQNILEELRDAPSNMNMMMMNQHPNMNDIPSFMDLHQQHEIPKAPPVNRPPEKRQRVPSAYNRFIKEEIQRIKAGNPDIG
HREAFSAAAKNWAHFPHIHFGLVPDNQPVKKTGMPQQEGEDNMGMKEGFYAPAANVGVTPY

>Schrenkiella parvula YAB2

MIQQILQIGLCDQVSVPYASLFTLVTVRCGYCNNLLSLNIGVSLHQGSPPPIHQDLQHKQHITSSVTRKECGSSSRGSNHFST
TSSEIVDREAPRMPSIRPPEKRQRVPSAYNRFIKEEIQRIKTGNPEISHREAFGAAAKNWAHFPHIHFGLKLDGNKKGKQLDQ
TVAGQKSNGYC

>Schrenkiella parvula YAB3

MSGMSMSSSSAPAYPPDHISSSDQLCYVHCRFCDTVLAVSVPPSSVFKTVTVRCGHCSNLLSVTVNMRALLLPPVSNLGHSSL
PPPPPPPLNLLEEMRNGGQNINMGMMMGHQAAAHHSGESLVMASRGGRVDLQEMPRPAPANRPPEKRQRVPSAYNRFIKEEIQ
RIKAGNPDIGHREAFSAAAKNWAHFPHIHFGLMPDHPPTKKANVRQQEGEDVMMGREGLYGSAANVGVTHN

>Schrenkiella parvula YAB5

MSNSTTTTTEQLCYIPCNFCNIVLAVSVPCGSLFDIVTVRCGHCTNLWSVNMAAALQSLSRPNFQATNYAMPEYGSSSRGHTK
IPSRISARTITEQRVVNRPPEKRQRVPSAYNQFIKEEIQRIKANNPDISHREAFGTAAKNWAHFPHIHFGLMLENNKQAKRA

>Schrenkiella parvula INO

MTTLNQLFDLPGQICHVQCGFCTTILLVSVPFTGLSMVVTVRCGHCTGLLSVNLMKASFIPLHLLTSLSPLDETGKEEVAATT
DGVEEEAWKVNQEKENSPTTLVTSSDNEDEDKDVSRVYQVVNKPPEKRQRAPSAYNCFIKEEIRRLKAQNPGMAHKEAFGLAA
KNWAHFPPVQNKRAASDQCFCEEDNNALLSCNVLEDREEGNNGCRERKAQRHSIWGKSPFE

>Schrenkiella parvula CRC

MNLEEKSTMASRASPQAEHLYYVRCGICNTILAVGIPLKRMLDTVTVKCGHCGNLSFLTTGPPLQGHVGLTLQMQGFGGNEYK
KGGSSSSSSSTSGDQPSSPRPPFVVKPPEKKQRLPSAYNRFMRDEIQRIKSANPEVPHREAFGAAAKNWAKYIPNSPTSTTSG
ANNINGFGFGEKK

>Sinapis alba FILa

MSMSSMSSPSSAVFSPENLSPDSLSPSEQLCYVQCNYCETILAVSVPYTSMFKTVTVRCGCCTNLISVNMRSLVLPASNQLQL
QLGPHSYFTPQNILEELKDAPSNMNMMMMNQHPNMNDIPSFMDLHQQHEIPKAPPVNRPPEKRQRVPSAYNRFIKEEIQRIKA
GNPDISHREAFSAAAKNWAHFPHIHFGLAPDNQPVKKTNMPQQESEDNMGMKEGFYAPAANVGVIPY

>Sinapis alba FILb

MSMSPMSSPSSAVFSPENLSPSDHLCYVQCNFCETILAVSVPYTSMFKTVTVRCGCCTNLLSVNMRSAALPASNQLQLQLGPH
SYFNTQNILEELRDAPSNMNMMMMNQHPNMNDIPSFMNIHQQHEIPKAPPVNRPPEKRQRVPSAYNRFIKEEIQRIKAGNPDI
SHREAFSAAAKNWAHFPHIHFGLAPDNQPVKKTNMPQQEGEDNMGMKEGFYAPAANVGVAPY
>Sinapis alba FILc
MSMSSPSSAVFSPEHLSPSEHLCYVQCNFCQTILAVSVPYTSLFKTVTVRCGCCTNLLSVNMRSLVLPASNQLQLGPQSYFTP
QNIMEEMREAPSNMNMMMINQHPNMNDIPSFMDLHQQHEIPKAPPANRPPEKRQRVPSAYNRFIKEEIQRIKAGNPDISHREA
FSAAAKNWAHFPHIHFGLMPNNQPVKKTNMPQQEGEENLGMKEGLYSPAASVGMAPY
>Sinapis alba YAB2a
MSVDLSSDRVCYVHCNFCTTILAVSVPYASLFSLVTVRCGHCTNLLSLNIGVSLHQSSPTPIHQDHQHKQHITSSVTRKEYGS
SSRSSNHFSTTLSENVDREAPRMPPIRPPEKRQRVPSAYNRFIKEEIQRIKAGNPEISHREAFSTAAKNWAHFPHIHFGLKLD
GNKKGKQLDQTVAGQKSNGYY
>Sinapis alba YAB2b
MSINDLSSERVCYVHCNFCTTILAVSVPYASLFTLVTVRCGHCTNLLSLNIGVSLHQSSPPPIHQDLHEQHITSSVTRKEHGS
SSRSCFNHFSTTSSENVEREAPRMPPIRPPEKRQRVPSAYNRFIKEEIQRIKAGNPQISHREAFSTAAKNWAHFPHIHFGLKL
DGNKKGKQIDQTVAGQKSNGYY
>Sinapis alba YAB2c
MSLDLSSERVCYVHCNFCTTILAVSVPYASLFTLVTVRCGHCTNLLSLNMGVSLHQSLPPPIHQDLQQPKQHITSSVTRKECG
SSSRSSNHFSTTLSENVDREAPRMPPIRPPEKRQRVPSAYNRFIKEEIQRIKSGNPAISHREAFSTAAKNWAHFPHIHFGLKL
DGNKKGKQLDQTVAGHKSYGYF
>Sinapis alba YAB3
MSSMSSSTPPYPPDHISSSDQLCYVHCSFCDTVLAVSVPPSSLFKTVTVRCGHCSNLLSVTVNMRALLLPSVSNIGHSFLPSP
PPPSPPNLLEEMRNGGQNINMNMMMSHQAAAHNSNESFVMATRNGRVDLQEMPRPPPPNRPPEKRQRVPSAYNRFIKEEIQRI
KAGNPDISHREAFSAAAKNWAHFPHIHFGLMPDHPPTKKANVRQQEGEEVMMGREGFYSSAANVGVTHN
>Sinapis alba YAB5
MANSAAAAEQLCYIPCNFCNIVLAVSVPCSSLFDIVTVRCGHCTNLWSVNMAAALQSLSRPNFQATPYATPEYGSSSRGHTKI
SSRISARTISEQRVVNRPPEKRQRVPSAYNQFIKEEIQRIKANNPDISHREAFSTAAKNWAHFPHIHFGLMLESNKQAKLA
>Sinapis alba INOa
MPNMTTLNQLFDMPGQICHVQCGFCTTILLVSVPFTSLSVVVTVRCGHCTSLLSVNLMKASFIPLHLLTSLSHLDEVPTGKEE
VAATTDGVEEEAWKVNQEKENSPTTLVTSSDNEDEDKDASRVYQVVNKPPEKRQRAPSAYNCFIKEEIRRLKAQNPSMAHKEA
FSLAAKNWAHFPPVQNKRAASDQCFCEEDNNVLLSCNALEDHEVSNNGFRERKAQRHSIWGKSPFE
>Sinapis alba INOb
MTKIPNMTTLNQLFDLPGQICHVQCGFCTTILLVSVPFTSLSMVVTVRCGHCTSLLSVNLMKASFIPLQLLTSLSHLDEAGKQ
EVAATTDGVEEEALKVNQEETSPTTLVTSSDNEDEDRDVSRVYQVVNKPPEKRQRAPSAYNCFIKEEIKRLKAQDPSLAHKEA
FSLAAKNWANFPPVQNKRAASDQCFCEDDNNALLSCNALEDHEESNKFRERKAQRHSIWGKSPFE
>Sinapis alba CRCa
MNLEEKPTMASRASPQAEHLYYVRCSICNTILAVGIPMKRMLDTVTVKCGHCGNLSFLTTTPPLQGHVSLTLQMQSFGGSDYK
KGSSSSSSSSTSSDQPPSPRPPFVVKPPEKKQRLPSAYNRFMRDEIQRIKSANPEIPHREAFSTAAKNWAKYIPNSPTSITSG
GSNINAFGFGEIKK
>Sinapis alba CRCb
MNLEEKPTMASRASPQAEHLYYVRCSICNTILAVGIPMKRMLDTVTVKCGHCGNLSFLTTTPPLQGHVSLTLQMQSFGGSDYK
KGSSSSSSSSTSSDQPPSPRPPFVVKPPEKKQRLPSAYNRFMRDEIQRIKSANPEIPHREAFSAAAKNWAKYIPNSPTSITSG
GSNINGFGFGEIKK
>Sinapis alba CRCc
MNLEEKSTLAPRASLQAEHLYYVRCSICNTILAVGIPLKRMLDTVTVKCGHCGSLSFLTTSHPLKGHVNLSLQMQSSSSGGSG
YKKGTSSSSSSSDQPSSPRPPFVVKPPEKKLRLPSAYNRFMRDEIQRIKSADPEIPHREAFSAAAKNWAQYIPNSPTSITSGA

NNINGFGFPEKK
>Sisymbrium irio FIL
MSMSSMSSPSSAVFSPEHLSPSDHLCYVQCNFCETILAVSVPYTSLFKTVTVRCGCCTNLLSVRTAVLPASNQLQLQLQLGPH
SYFNTQNILEELRDAPSNMNMMMMNQHPNMNDIPSFMDLHQQHEIPKAPPVNRPPEKRQRVPSAYNRFIKEEIQRIKAGNPDI
SHREAFSAAAKNWAHFPHIHFGLVPDNQPVKKTNMPQQEGEDNMGMKEGFYAPAANVGVAPY
>Sisymbrium irio YAB2a
MSIDLSSERVCYVHCSFCTTILAVSVPYASLFTLVTVRCGHCTNLLSLNIGVSLHQSAPPPVHQDLQHKQHITSSVTRKECGS
SSRSSNHFSTTLSENVDREAPRMPPIRPPEKRQRVPSAYNRFIKEEIQRIKAGNPEISHREAFSTAAKNWAHFPHIHFGLKLD
GNKKGKQLDQTVAGQKSNGYY
>Sisymbrium irio YAB2b
MSMDLSSERVCYVHCNFCTTVLAVSVPYASLFTLVTVRCGHCTNFLSLNIGVSLHQSSLPPVHQDLQHKQHVTSSVTRKECGS
SSRSTNPFSTTFSENVGREAPRMPPMRPPEKRQRVPSAYNRFIKEEIQRIKAGNPEISHREAFSTAAKNWAHFPQIRFGLKLD
GNKKGKQSDQTVAGQKSNGYY
>Sisymbrium irio YAB3
MSSMSMSSSSAPAYSPDHISSTDQLGYVHCNFCDTVLAVSVPPSSLFKTVTVRCGHCSNLLSVTVNMRAVLLPSVSNLGHSLL
PPSPPPPNLLEEMRNGGQNINMNMMMSHQAAAHHSNESLVMATRNGRVDQQEIPRPPPANRPPEKRQRVPSAYNRFIKEEIQR
IKAGNPDISHREAFSAAAKNWAHFPHIHFGLMPDHPPTKKANVRQQEGEDVMMGREGFYGSAANVGVT
HN
>Sisymbrium irio YAB5
MADSAMATEQLCYIPCNFCNIVLAVSVPCSSLFEIVTVRCGHCTNLWSVNMAAALQSLSRSNYQ
>Sisymbrium irio INO
MPNMTTLNQLFDLPGQICHVQCGFCTTILLVSVPFTSLSMVVTVRCGHCTSLLSVNLMKASFIPLHLLTSLSHLDEAGKEEVA
ATTDGVEEEACKVPQEKDNSPTTLVTSSDNEDEDRDVSRVYQVVNKPPEKRQRAPSAYNCFIKEEIRRLKSQNPSMAHKEAFS
LAAKNWAHFPPVQNKRAASDHCFCEDDNSALLSCNVLEDHEESNNGFRERKAQRHSIWGKSPFE
>Sisymbrium irio CRC
MNLEEKSTMASRASPQAEHLYYVRCSICNTILAVGIPMKRMLDTVTVKCGHCGNLSFLTTSPPLQGHVSLTLQMQSFGGSEYK
KGSSSSSSSSTSSEQPPSPRPPFVVKPPEKKQRLPSAYNRFMRDEIQRIKGANPEIPHREAFSAAAKNWAKYVPNSPTSITSG
ASNINK
>Stanleya pinnata YAB3
YSPDHISSTDQLGYVHCSFCDTVLAVSVPPSSLFKTVTVRCGHCSNLLSVTVNMRALLLPSVSNLGHSLLPPPPPPPNLLEEM
RNGGQNINMNMMMSHQAAAHHSNESLVMATCNGRVDQQEMHRPPPANRPPEKRQRVPSAYNRFIKEEIQRIKAGNPDISHREA
FSAAAKNENILIKSHWGNVCIYYM
>Stanleya pinnata YAB5a
MANSTMATEQLCYIPCNFCNIVLAVSVPCSSLFEIVTVRCGHCTNLWSVNMAAALQSLSRPNFQATNYPMPEYGSSTRGHSKT
PSRISARTITEQRVVNRPPEKRQRVPSAYNQFIKEEIQRIKANNPDISHREAFSTAAKNWAHFPRIHFGLMLESNKQAKLA
>Stanleya pinnata YAB5b
MANSETATEQLRYIPCNFCNIVLAVSVPCSSLFDIVTVRCGHCTNLWSVNMAAALQSLSRPNFQATNYAMPEYGSSSRGHTKI
PSRLSAGTITEQRVVNRPPEKRQRVPSAYNQFIKEEIQRIKANNRDISHREAFITAAKNWAHFPHIHFGLMLESNKQA
>Stanleya pinnata CRCa
MNLEEKSTMASRASPQAEHLYYVRCSICNTILAVGIPMKRMLDTVTVKCGHCGNLSFLTTSPPLQGHVSLTLQSFGGNEYKKG
SSSSSSSSTSSDQPPSPRPPFVVKPPEKKQRLPSAYNRFMRDEIQRIKSANPEIPHREAFSAAAKNWAKYVPNSPTSITSGAS
NINGFGFGEKK
>Stanleya pinnata CRCb
MNLEEKPTMGSRASPQAEHLYYVRCSICNTILAVGIPLKRMLDTVTVKCGHCGNLSFLTTSPPLQGHVSLTLQMQSFGGSEYK
KGSSSSSSSSTSSDQPPSPRPPFVVKPPEKKQRLPSAYNRFMRDEIQRIKSANPEIPHREAFSAAAKNWAKYIPNSPTSGANN

VNGFVFGEKK

>Thellungiella halophila FIL

MSMSSPSSAVFSPEHLSPSEHLCEHLCYVQCKFCETILAVSVPYTSLFKTVTVRCGCCTNLLSVNMRPVVLPASNQLQLQLGP

HSYMSPQNLLEELRDAPSNMNMMMMNQHPNMNDIPSFMDLHQQHEMPKAPTVNRPPEKRQRVPSAYNRFIKEEIQRIKAGNPD

ISHREAFSAAAKNWAHFPHIHFGLVPDNQPVKKTNMPQQEGDDNMVMKEGFYAPAANVGVSPY

>Thellungiella halophila YAB2

MSIDLSSERVCYVHCNFCTTILAVSVPYASLFTLVTVRCGHCTNLLSLNIGVSLHQTSAPPIHQDLQQHKQHITSPVTRKDFG

SSSRSSNHFSTTLSENVDRDQAPRMPPIRPPEKRQRVPSAYNRFIKEEIQRIKAGNPEISHREAFSTAAKNWAHFPHIHFGLK

LDGNKKGKQLDQTVAGQKSNGYY

>Thellungiella halophila YAB3

MSSMSMSSSSDPAYPPDHISSLDQLGYVHCSFCDTVLAVSVPPSSLFKTVTVRCGHCSNLLSVTVNMRALLLPPVSNLGHSFL

PPPPPCNLLEEMRNGGQNINMNMMMSHQAAAHHSNESLVMATRNGRVDPQELMPRPPPANRPPEKRQRVPSAYNRFIKEEIQR

IKAGNPDISHREAFSAAAKNWAHFPHIHFGLMPDHPPTKKANVRQQEGEDVMLGREGFYGSSAANVGVTHN

>Thellungiella halophila YAB5

MANSATATEQLCYIPCNFCNIVLAVSVPCSSLFDIVTVRCGHCTNLWSVNMVAALQSLSRPNFQATNYAMSEHGSSSRGHTKI

PSRISTRTITEQRVVNRPPEKRQRVRSAYNQFIKEEIQRIKANNPNISHREAFSTAAKNWAHFPHIHFGLMLESNKQAKIA

>Thellungiella halophila INO

MPNLTTLNHLFDLPGQICHVQCGFCTTILLVSVPFTSLSMVVTVRCGHCTSLLSVNLMKASFIPLHLLASLSHLDEAGKEDVN

VATTDGVEEEAWKANEEKENSPATLVTSSDNEDEDKDVSRVYQVVNKPPEKRQRAPSAYNCFIKEEIRRLKAQNPRMAHKEAF

SLAAKNWAHFPPVQTKRAASDQCFCEEDNNTVLPCNALEDHEESNNGFRERKAQRHSIWGKSPFE

>Thellungiella halophila CRC

MNLEEKPTMASRASSQAEHLYYVRCSICNTILAVGIPLKRMLDTVTVKCGHCGNLSFLTTSPPLQGHVSLTLQMQSFGGSHEY

KKGSSSSSSSSTSSDQPPSPRPPFVVKPPEKKQRLPSAYNRFMRDEIQRIKSANPEIPHREAFSAAAKNWAKYIPNSPTSITS

GGNNINGLGFGEKK

>Thellungiella salsuginea FIL

MSMSSPSSAVFSPEHLSPSEHLCEHLCYVQCKFCETILAVSVPYTSLFKTVTVRCGCCTNLLSVNMRPVVLPASNQLQLQLGP

HSYMSPQNLLEELRDAPSNMNMMMMNQHPNMNDIPSFMDLHQQHEMPKAPTVNRPPEKRQRVPSAYNRFIKEEIQRIKAGNPD

ISHREAFSAAAKNWAHFPHIHFGLVPDNQPVKKTNMPQQEGDDNMVMKEGFYAPAANVGVSPY

>Thellungiella salsuginea YAB2

MSIDLSSERVCYVHCNFCTTILAVSVPYASLFTLVTVRCGHCTNLLSLNIGVSLHQTSAPPIHQDLQQHKQHITSPVTRKDFG

SSSRSSNHFSTTLSENVDRDQAPRMPPIRPPEKRQRVPSAYNRFIKEEIQRIKAGNPEISHREAFSTAAKNWAHFPHIHFGLK

LDGNKKGKQLDQTVAGQKSNGYY

>Thellungiella salsuginea YAB3

MSSMSMSSSSDPAYPPDHISSLDQLGYVHCSFCDTVLAVSVPPSSLFKTVTVRCGHCSNLLSVTVNMRALLLPPVSNLGHSFL

PPPPPCNLLEEMRNGGQNINMNMMMSHQAAAHHSNESLVMATRNGRVDPQELMPRPPPANRPPEKRQRVPSAYNRFIKEEIQR

IKAGNPDISHREAFSAAAKNWAHFPHIHFGLMPDHPPTKKANVRQQEGEDVMLGREGFYGSSAANVGVTHN

>Thellungiella salsuginea YAB5

MANSATATEQLCYIPCNFCNIVLAVSVPCSSLFDIVTVRCGHCTNLWSVNMVAALQSLSRPNFQATNYAMSEHGSSSRGHTKI

PSRISTRTITEQRVVNRPPEKRQRVRSAYNQFIKEEIQRIKANNPNISHREAFSTAAKNWAHFPHIHFGLMLESNKQAKIA

>Thellungiella salsuginea INO

MPNLTTLNHLFDLPGQICHVQCGFCTTILLVSVPFTSLSMVVTVRCGHCTSLLSVNLMKASFIPLHLLASLSHLDEAGKEDVN

VATTDGVEEEAWKANEEKENSPATLVTSSDNEDEDKDVSRVYQVVNKPPEKRQRAPSAYNCFIKEEIRRLKAQNPRMAHKEAF

SLAAKNWAHFPPVQTKRAASDQCFCEEDNNTVLPCNALEDHEESNNGFRERKAQRHSIWGKSPFE

>Thellungiella salsuginea CRC

MNLEEKPTMASRASSQAEHLYYVRCSICNTILAVGIPLKRMLDTVTVKCGHCGNLSFLTTSPPLQGHVSLTLQMQSFGGSHEY

KKGSSSSSSSSTSSDQPPSPRPPFVVKPPEKKQRLPSAYNRFMRDEIQRIKSANPEIPHREAFSAAAKNWAKYIPNSPTSITS
GGNNINGLGFGEKK

>Thlaspi arvense FIL
MSMSSMSSPSSAVFSPEPISPSEQLCYVRCDFCQTILAVSVPYTSLFKTVTVRCGCCTNLLSVNMRSLVLPASNQLQLQLGPH
SYFNTQDILEELRDAPSNMNMMMMNQHPNMNDMPSFMDLHQQHEIPKAPPVNRPPEKRQRVPSAYNRFIKEEIQRIKAGNPDI
SHREAFSAAAKNWAHFPHIHFGLVPDNQPVKKTNMPQQEGEDNMVMKEGFYAPAANVGVTPY
>Thlaspi arvense YAB2
MSIDLSSERVCYVHCNFCTTILAVSVPYASLFTLVTVRCGHCTNLLSLNIGVSLHQNSPPPIHQELQQHKQHITSSVTRKDCG
SSSRSTNHFSTTLSENVDRETPRMPPIRPPEKRQRVPSAYNRFIKEEIQRIKAGNPEISHREAFSTAAKNWAHFPHIHFGLKL
DGNKKGKQLDQTVAGQKSNGYY
>Thlaspi arvense YAB3
MSSMSMSSSSAPPVYPPDHISSSEQLCYVHCSFCDTVLAVSVPPSSLFKTVTVRCGHCSNLLSVTVHMRTLLLPSVSNLGHSF
LPPPPPPNLLEEMRNGGQNINMNMMMSHQAAAAYHSNESLAMATRNGRVDPQETPRPPPANRPPEKRQRVPSAYNRFIKEEIQ
RIKAGNPDISHREAFSAAAKNWAHFPHIHFGLMPDHPPAKKANVRQQEGEDAMMGREGFYGAAANVGVTHN
>Thlaspi arvense YAB5
MASATEQLCYIPCNFCNIVLAVSVPSSSLFDIVTVRCGHCTNLWSVNMAAALQSLSRPNFQATNYAMPEYGSSSRGHTKIPSR
ISTRTITEQRVVNRPPEKRQRVPSAYNQFIKEEIQRIKANNPDISHREAFSTAAKNWAHFPHIHFGLMLESNKQAKLA
>Thlaspi arvense INO
MTRIPNMTTLNHLFDLPGQICHVQCGFCTTILLVSVPFTSLSMVVTVRCGHCTSLLSVNLLKASFIPLHLLTSLSHLDEAGKE
EVNAATTDGVEEETWKVAQEKESSPTTLVTSSDNEDEDKDVSRVYQVVNKPPEKRQRAPSAYNCFIKEEIRRLKAQNPSMAHK
EAFSLAAKNWAHFPPAQNKRAASDQCFCEEDNNTVLPCNALEDREESNNGFRERKAQRHSIWGKSPFE
>Thlaspi arvense CRC
MNLEDKPTMTSRASPQSEHLYYVRCSICNTILAVGIPLKRMLDTVTVKCGHCGNLSFLTTSPPLHGHVSLTLQMQSFGGNEYK
KGSSSSSSSSTSSDQPPSPRPPFVVKPPEKKQRLPSAYNRFMRDEIQRIKSANPEIPHREAFSAAAKNWAKYIPNSPTSITSG
GNNIHGCLGFGEKK
